# The comparative effectiveness of migraine preventive drugs: a systematic review and network meta-analysis

**DOI:** 10.1186/s10194-023-01594-1

**Published:** 2023-05-19

**Authors:** Christian Lampl, Antoinette MaassenVanDenBrink, Christina I. Deligianni, Raquel Gil-Gouveia, Tanvir Jassal, Margarita Sanchez-del-Rio, Uwe Reuter, Derya Uluduz, Jan Versijpt, Dena Zeraatkar, Simona Sacco

**Affiliations:** 1 Department of Neurology, Konventhospital Barmherzige Brüder Linz, Linz, Austria; 2 Headache Medical Center Linz, Linz, Austria; 3 Department of Internal Medicine, Erasmus MC Medical Center, Rotterdam, The Netherlands; 4 Department of Neurology, Athens Naval Hospital, Athens, Greece; 5 Neurology Department, Hospital da Luz Headache Center, Hospital da Luz Lisboa, Lisbon, Portugal; 6 Center for Interdisciplinary Research in Health, Universidade Católica Portuguesa, Lisbon, Portugal; 7 Department of Anesthesia and Department of Health Research Methods, Evidence and Impact, McMaster University, Hamilton, Canada; 8 Department of Neurology, Clinica Universidad de Navarra, Madrid, Spain; 9 Department of Neurology, Charité Universitätsmedizin Berlin, Berlin, Germany; 10 Department of Neurology Istanbul Cerrahpasa Medical Faculty, Istanbul, Turkey; 11 Department of Neurology, Vrije Universiteit Brussel (VUB), Universitair Ziekenhuis Brussel (UZ Brussel), Brussels, Belgium; 12 Department of Biotechnological and Applied Clinical Sciences, University of L´Aquila, L’Aquila, Italy

**Keywords:** Migraine, CGRP monoclonal antibodies, Systematic review, Network meta-analysis

## Abstract

**Objective:**

While there are several trials that support the efficacy of various drugs for migraine prophylaxis against placebo, there is limited evidence addressing the comparative safety and efficacy of these drugs. We conducted a systematic review and network meta-analysis to facilitate comparison between drugs for migraine prophylaxis.

**Methods:**

We searched MEDLINE, EMBASE, CENTRAL, and clinicaltrials.gov from inception to August 13, 2022, for randomized trials of pharmacological treatments for migraine prophylaxis in adults. Reviewers worked independently and in duplicate to screen references, extract data, and assess risk of bias. We performed a frequentist random-effects network meta-analysis and rated the certainty (quality) of evidence as either high, moderate, low, or very low using the GRADE approach.

**Results:**

We identified 74 eligible trials, reporting on 32,990 patients. We found high certainty evidence that monoclonal antibodies acting on the calcitonin gene related peptide or its receptor (CGRP(r)mAbs), gepants, and topiramate increase the proportion of patients who experience a 50% or more reduction in monthly migraine days, compared to placebo. We found moderate certainty evidence that beta-blockers, valproate, and amitriptyline increase the proportion of patients who experience a 50% or more reduction in monthly migraine days, and low certainty evidence that gabapentin may not be different from placebo. We found high certainty evidence that, compared to placebo, valproate and amitriptyline lead to substantial adverse events leading to discontinuation, moderate certainty evidence that topiramate, beta-blockers, and gabapentin increase adverse events leading to discontinuation, and moderate to high certainty evidence that (CGRP(r)mAbs) and gepants do not increase adverse events.

**Conclusions:**

(CGRP(r)mAbs) have the best safety and efficacy profile of all drugs for migraine prophylaxis, followed closely by gepants.

**Supplementary Information:**

The online version contains supplementary material available at 10.1186/s10194-023-01594-1.

## Introduction

Migraine is a debilitating disorder that significantly burdens affected individuals [1–3], ictal as well as in the interictal phase3, indicating migraine as the costliest neurological disorder [4]. Several classes of medication are commonly used for migraine prophylaxis, including antidepressants, anticonvulsants, antihypertensives, gepants, and calcitonin gene-related peptide (receptor) monoclonal antibodies (CGRP(r)mAbs. Previous trials and systematic reviews have demonstrated the efficacy of these drugs. Newer drugs, like CGRP(r)mAbs and gepants, although proven effective and well tolerated, are mostly available at a much higher cost restricting access to their use [5]. While there is a body of evidence that investigates the efficacy and safety of migraine preventive drugs, there is limited evidence on their comparative efficacy with each other [6–9], due to which guidelines have been unable to generate hierarchies to guide selection among all options [10]. Only one trial to date has compared CGRP(r)mAbs with topiramate [11].

We present a systematic review and network meta-analysis to facilitate comparison between these drugs. A network meta-analysis provides information on the comparative effectiveness of three or more drugs across a network of studies, including drugs that have not been directly compared in clinical trials [12].

## Methods

We submitted a protocol to the European Headache Federation in September 2022 and registered our protocol on Open Science Framework (https://osf.io/apdhf). We present our methods and results following the Preferred Reporting Items for Systematic Reviews and Meta-Analyses extension for network meta-analyses (PRISMA-NMA) [13, 14].

### Search strategy

In consultation with an experienced research librarian, we searched MEDLINE, EMBASE, Cochrane CENTRAL, and ClinicalTrials.gov from inception to August 13, 2022, for randomized trials of pharmacologic treatments for migraine prophylaxis, without language restrictions. Our search combined terms related to randomized trials, migraine, and drugs for migraine prophylaxis using Boolean operators (Supplement 1). We supplemented our search by retrieving references of similar systematic reviews and meta-analyses [6–9, 15–17]. Following training and calibration exercises to ensure sufficient agreement, pairs of reviewers, working independently and in duplicate, reviewed titles and abstracts of search records and subsequently the full texts of records deemed potentially eligible at the title and abstract screening stage. Reviewers resolved discrepancies by discussion, or, when necessary, by adjudication with a third reviewer.

### Screening and study eligibility

We included parallel group, randomized controlled trials with episodic or chronic migraine in adults, according to any diagnostic criteria, to pharmacologic interventions for migraine prophylaxis or placebo. Trials addressing antidepressants, antiepileptics, antihypertensives, CGRP(r)mAbs, calcium channel blockers and gepants were enclosed. We considered also including botulinum toxin but found significant clinical and statistical heterogeneity, precluding inclusion in the network. There is convincing evidence, for example, that the effect of botulinum toxin is different based on chronic versus episodic migraine [18, 19]. Other sources of heterogeneity included techniques for injection and type of neurotoxin. We considered producing separate networks for chronic and episodic migraine. However, this would eliminate many trials that do not report results stratified by type of migraine.

We excluded trials that investigated abortive rather than prophylactic interventions and trials conducted in children or adolescents’ cluster. For feasibility, we excluded trials that had fewer than 100 participants randomized. Given existing large trials, smaller trials were less likely to meaningfully contribute to the analysis. While this decision limited the number of eligible trials, it is unlikely to have biased the results. Smaller trials are more likely to be single-centre studies with potentially unrepresentative samples of participants and are at higher risk of publication bias [20].

### Data extraction

We extracted data on trial characteristics (e.g., country), patient characteristics (e.g., episodic vs. chronic migraine), diagnostic criteria, intervention characteristics (e.g., dose and duration), and outcomes of interest at the longest reported follow-up time at which patients were still using the interventions being investigated. Our outcomes of interest were informed by the Core Outcome Set for preventive intervention trials in chronic and episodic migraine (COSMIG) and include proportion of patients who experience a 50% or more reduction in migraine days per month, number of migraine days per month, and adverse events leading to discontinuation [21]. We prioritized extracting monthly migraine days when reported but also extracted monthly headache days or monthly migraine attacks when monthly migraine days was not reported. We also prioritized extracting data from intent-to-treat analyses, when reported.

### Risk of bias assessments

To assess any risk of bias a modified Cochrane RoB 2.0 tool was used [22, 23]. For each trial, we rated each outcome as either ‘low risk of bias’, ‘some concerns –probably low risk of bias’, ‘some concerns –probably high risk of bias’, and ‘high risk of bias’ across the following domains: bias arising from the randomization process, bias due to departures from the intended intervention, bias due to missing outcome data, bias in measurement of the outcome, bias in selection of the reported results. Supplement 2 presents additional details about risk of bias assessments.

### Data synthesis and analysis

For all outcomes, we performed frequentist random-effects network meta-analysis using the restricted maximum likelihood (REML) estimator. Our choice of frequentist over Bayesian network meta-analysis was motivated by feasibility and simplicity of the model and evidence indicating that the two models generate similar results in most situations [12, 24]. Our choice of the random-effects model over the fixed effect model was informed by the potential differences in effect estimates across trials due to differences in study design, patient populations, methods for administering the intervention, and duration of follow-up [12]. We estimated relative risks (RRs) for 50% or more reduction in monthly migraine days, mean differences (MDs) for monthly migraine days, and risk differences (RDs) for adverse events leading to discontinuation.

For our primary analysis, we classified drugs into the following nodes, regardless of dose: amitriptyline, beta-blockers, calcium channel blockers, carisbamate, gabapentin, gepants, oxcarbazepine, pregabalin, topiramate and valproate. We also included each of the CGRP(r)mAbs as separate nodes, to facilitate comparisons between them and because we anticipated that their effects may be different due to differences in their biological targets, pharmacodynamics, and pharmacokinets. We grouped beta-blockers, calcium channel blockers, gepants, and gabapentin/pregabalin because we anticipated similar efficacy and safety, thereby maximizing the statistical power of our analysis.

We also performed three secondary analyses for 50% or more reduction in monthly migraine days and adverse events leading to discontinuation. The first secondary analysis included all CGRP(r)mAbs. in the same node, the second was restricted to trials that tested recommended therapeutic doses of the drugs (Supplement 3), and the third included each of the gepants as separate node. To facilitate interpretation, we report dichotomous outcomes as number of events per 1,000 patients, calculated using the median baseline in the placebo arms across trials, by multiplying the baseline risk by the estimated RR. We summarize heterogeneity using the I^2^ statistic and interpret an I^2^ value of 0% to 40% as not important, 30% to 60% as moderate heterogeneity, and 50% to 90% as substantial heterogeneity, and 75% to 100% may represent considerable heterogeneity [12].

We assessed for local incoherence—defined as differences in estimates between direct and indirect comparisons—by node-splitting [25]. For comparisons with 10 or more studies, we planned to test for publication bias by visually inspecting funnel plots and Eggers test. None of the comparisons, however, included 10 or more studies [26]. Network meta-analyses can also provide rankings of treatments that are most likely to be superior. We avoid these ranking approaches, however, since they fail to account for the precision of ranking estimates or the certainty of evidence [27].

We anticipated that trials at high risk of bias may overestimate the beneficial effects of treatments and that trials that recruit patients with more severe migraine or patients who had previously used prophylactic treatments may be less likely to report beneficial effects. For 50% or more reduction in monthly migraine days and adverse events leading to discontinuation, we performed pairwise meta-regressions comparing results of trials rated at low versus high risk of bias and trials below versus above the median number of monthly migraine days or proportion of patients that had previously used prophylactic therapy across trials. Telcagepant displayed efficacy in clinical trials but was discontinued due to safety concerns. Therefore, we performed a sensitivity analysis excluding telcagepant. We, however, retained telcagepant in the primary analysis to maximize power, since we anticipated similar efficacy compared to other gepants [28]. We assessed the credibility of subgroup effects using the ICEMAN tool [29].

We performed all analyses using the *meta* and *netmeta* packages in R (Vienna, Austria; Version 4·1·2) and produced network plots using the networkplot command in Stata version 15·1 [30, 31]. We defined statistical significance at a 2-sided α level of less than 0.05. The data and code to reproduce the results presented in this manuscript, as well as additional model diagnostics, and leverage plots are stored on Open Science Framework (https://osf.io/2afk8/).

### Assessment of the certainty (quality) of evidence

We assessed the certainty of evidence using the GRADE approach for network meta-analysis [25, 32, 33]. For each outcome, we rated certainty of each comparison as either high, moderate, low, or very low based on: risk of bias (study limitations), inconsistency (differences between the results of trials), indirectness (differences between the questions investigated in trials and the question of interest), publication bias (propensity for statistically significant or interesting results to be published or published faster or published in journals with higher visibility), intransitivity (differences in trial characteristics across comparisons), incoherence (difference between direct and indirect effects), and imprecision (random error). High certainty evidence indicates situations in which we have high certainty that the true effect lies close to estimated effect and low or very low certainty evidence indicates situations in which the true effect may be substantially different from the estimated effect. We made judgements regarding imprecision using the minimally contextualized approach [34], which considers only whether confidence intervals include the null effect and thus does not consider whether plausible effects, captured by confidence intervals, include both important and trivial effects. To evaluate the certainty of no effect, we used minimally important differences, sourced by consensus from the authors [34]. Results were reported by using GRADE simple language summaries (i.e., describing high certainty evidence with declarative statements, moderate certainty evidence with ‘probably’, low certainty evidence with ‘may’ and very low indicated by ‘very uncertain’) [35].

## Results

### Search results

**Fig. 1:**
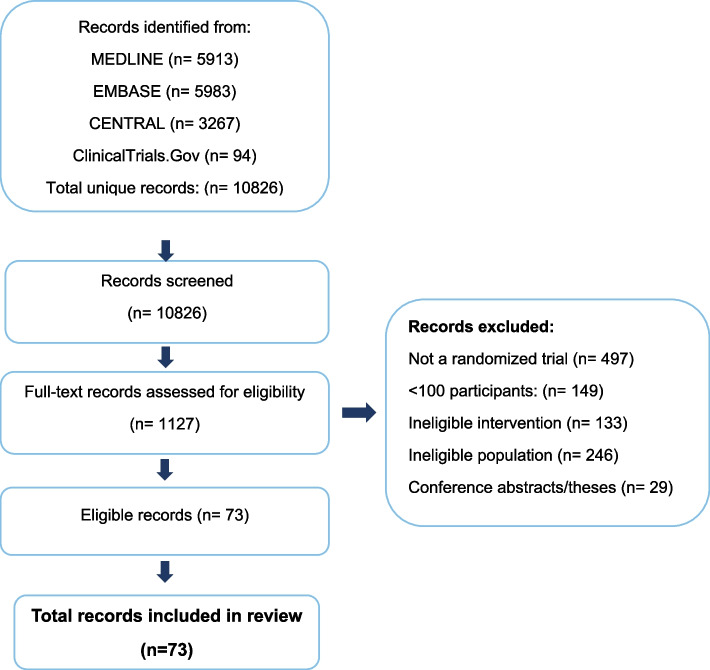
Selection of trials

Our search yielded 10,826 unique references. We identified 73 eligible publications reporting on 74 unique trials with 32,990 participants [11, 36–107]. All trials were published in peer-reviewed journals in English. Figure 1 provides additional details regarding study selection.

### Trial and patient characteristics

Table 1 and Supplement 4 present trial and participant characteristics. Seven trials (802) reported on amitriptyline, 13 (1,361 patients) reported on beta-blockers (propranolol, bisoprolol, and metoprolol), 8 (1046 patients) on calcium channel blockers (flunarizine, nimodipine, cinnarizine), 1 (243 patients) on carisbamate, 5 (2629 patients) on eptinezumab, 9 (2830 patients) on erenumab, 7 (2883 participants) on fremanezumab, 3 (566 patients) on gabapentin or pregabalin, 7 (2,112 patients) on galcanezumab, 4 (2055 patients) on gepants (atogepant, rimegepant, telcagepant), 1 (85 patients) on oxcarbazepine, 13 (2,698 patients) on topiramate, and 8 (793 patients) on valproate. Notably, no studies on angiotensin II receptor type 1 antagonists met eligibility criteria, and as described above we excluded botulinum toxin due to clinical and statistical heterogeneity. Trials typically recruited patients with migraine according to the International Classification of Headache Disorders criteria [108]. Few trials addressed chronic migraine. Most trials started with a run-in period during which patients recorded their symptoms in headache diaries to ensure eligibility before randomization. Most patients were female. Most trials were funded by pharmaceutical companies.

**Table 1:** Trial characteristics

**Registered**	**44 (59%)**
**Funding**	
** Industry**	**54 (73%)**
Government	4 (5%)
Institution	2 (3%)
Not-for-profit	0 (0%)
None	1 (1%)
Not reported	13 (17%)
** Mean age**	41
** Male (%)**	16%
** Migraine with aura (%)**	18 (45%)
** Prior prophylaxis (%)**	28 (49%)
** Mean migraine days/month**	11
** Interventions**	
** Amitriptyline**	7 (10%)
Beta-blockers	13 (18%)
Calcium Channel Blockers	8 (11%)
Carismabate	2 (3%)
Eptinezumab	5 (7%)
Erenumab	9 (13%)
Fremanezumab	7 (10%)
Gabapentin	2 (3%)
Galcanezumab	7 (10%)
Gepants	4 (6%)
Oxcarbazepine	1 (1%)
Pregabalin	1 (1%)
Topiramate	13 (18%)
Valproate	8 (11%)

### Risk of bias

**Fig. 2:**
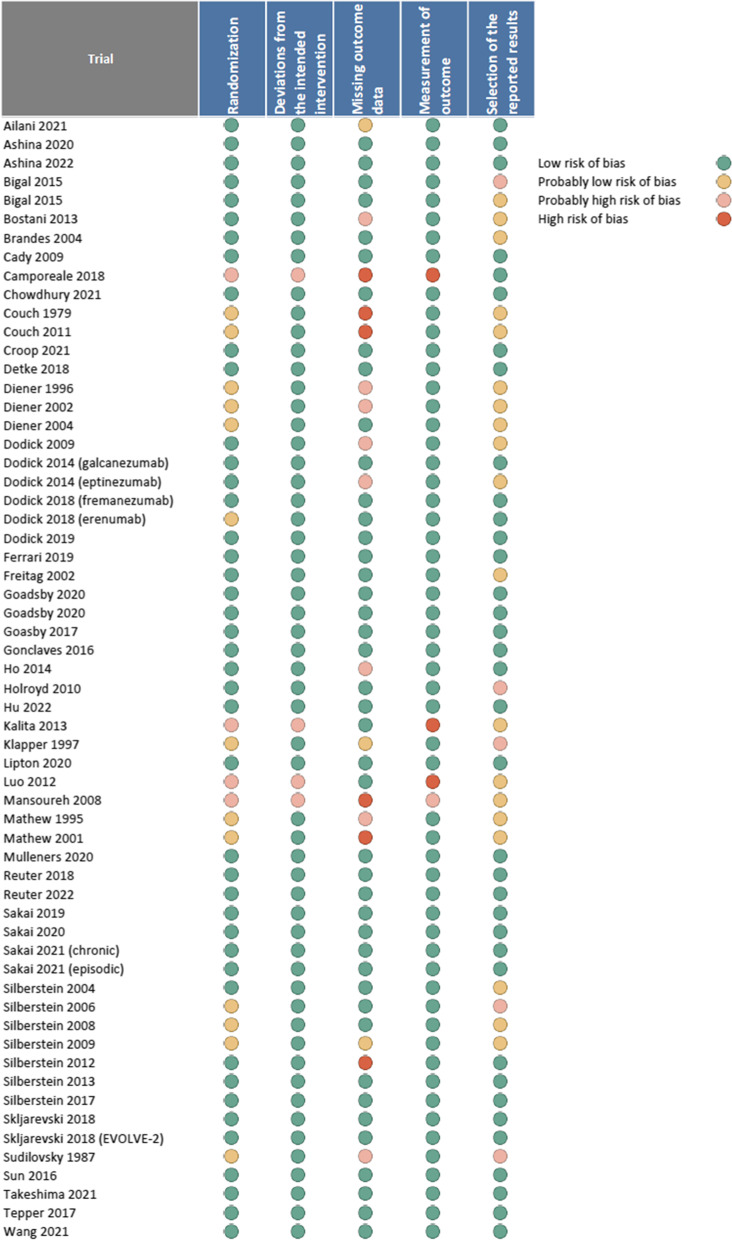
Risk of bias judgements for 50% or more reduction in monthly migraine days

Among 60 trials that reported on 50% or more reduction in monthly migraine days, we judged 20 (33.3%) to be at high risk of bias [38, 40, 43, 45, 46, 49, 50, 54, 56, 69, 70, 72, 73, 77, 78, 80, 81, 91, 94, 99]. Among 69 trials that reported on adverse events leading to discontinuation, we judged 20 (29%) to be at high risk of bias [43, 45, 49, 50, 54, 63, 69, 72, 74, 76–80, 82, 89, 98, 99, 107]. Among 62 trials that reported on monthly migraine days, we judged 23 (37%) to be at high risk of bias [38–40, 43, 50, 54, 56, 63, 67–69, 74, 76–78, 80, 81, 89, 91, 93, 94, 98]. We judged the remaining trials to be at low risk of bias. Missing outcome data and failure to blind or conceal allocation were common reasons due to which trials were rated at high risk of bias. Figure 2 presents risk of bias assessments for 50% reduction in monthly migraine days and Supplements 5 and 6 presents risk of bias assessments for monthly migraine days and adverse events leading to discontinuation.

### 50% or more reduction in monthly migraine days

**Fig. 3:**
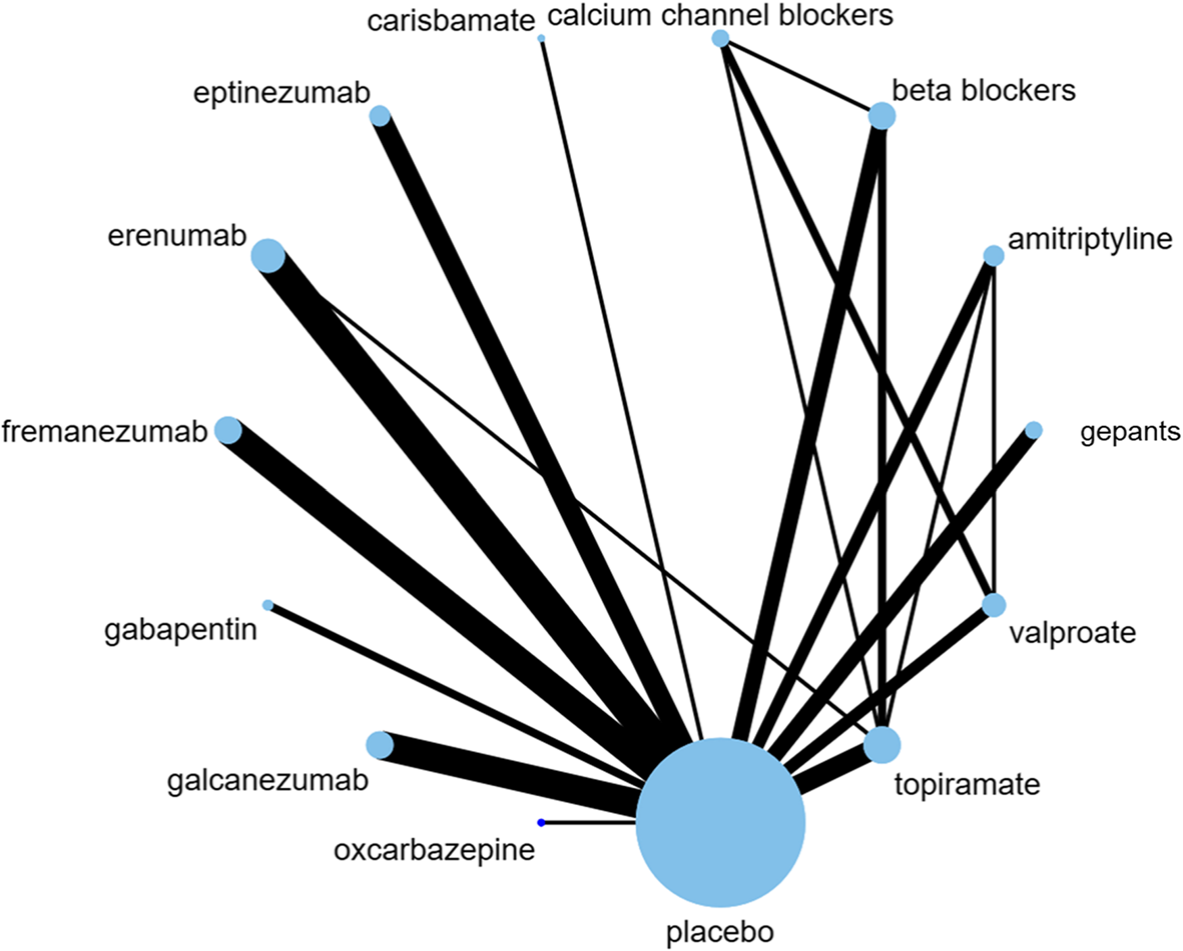
Network geometry for 50% or more reduction in monthly migraine days. Each node represents a drug that has been tested in trials. The edges represent direct comparisons of the drugs in trials. The size of the nodes is proportional to the number of patients that have received that drug, and the thickness of the edges is proportional to the number of trials

**Fig. 4:**
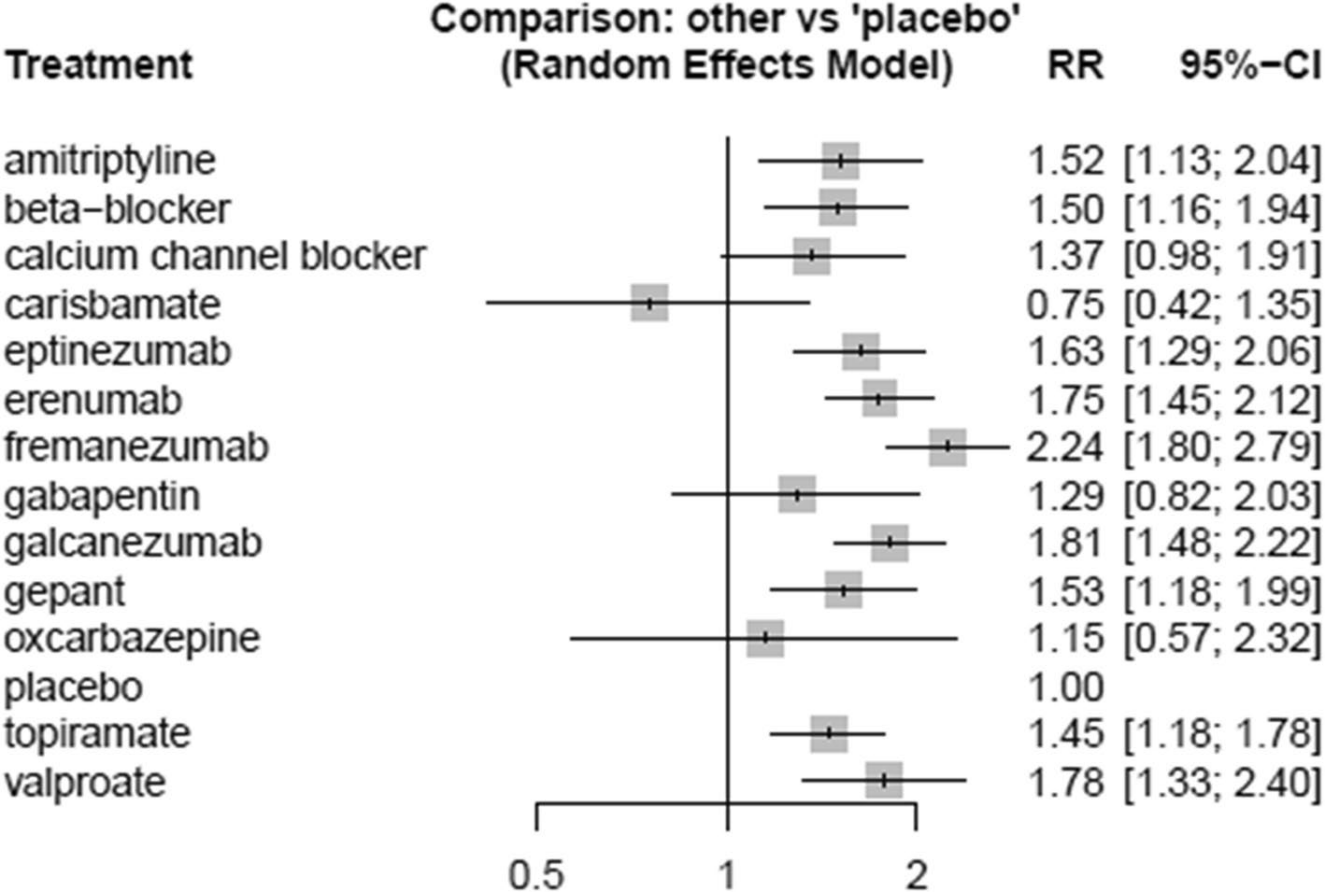
Forest plot displaying results for 50% or more reduction in monthly migraine days

Fifty-seven trials with 26,378 patients reported on 50% or more reduction in monthly migraine days and could be incorporated into the network meta-analysis [11, 36–42, 44–51, 54–61, 64, 65, 67, 69–73, 75, 77, 78, 80, 81, 83–88, 90–97, 100–102, 104–106]. Figure 3 presents the geometry of the network. Table 2 and Fig. 4 present the results of the network meta-analysis for comparisons against placebo and Supplement 7 presents results and GRADE ratings for all other comparisons. We found high certainty evidence that fremanezumab, eptinezumab, erenumab, galcanezumab, gepants, and topiramate increase the proportion of patients who experience a 50% or more reduction in monthly migraine days compared to placebo. We found moderate certainty evidence that beta-blockers, valproate, and amitriptyline probably increase the proportion of patients who experience a 50% or more reduction in monthly migraine days and that carisbamate and oxcarbazepine are probably not different than placebo. Finally, we found low certainty evidence that gabapentin may increase the proportion of patients who experience a 50% or more reduction in monthly migraine days and very low certainty evidence for calcium channel blockers.

**Table 2:**
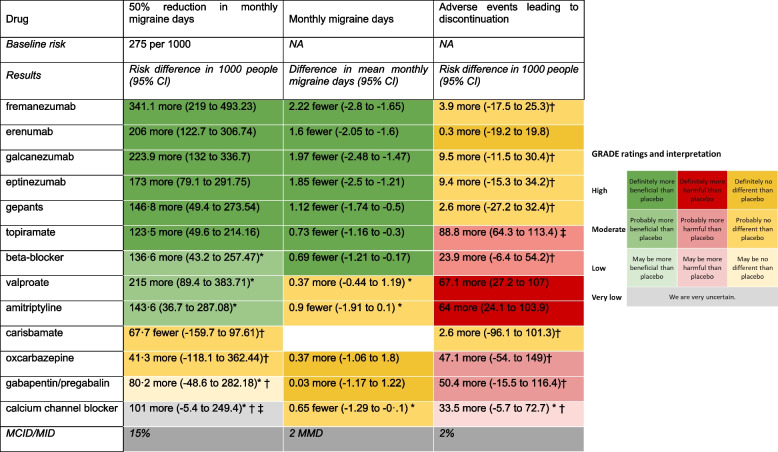
Results of the network meta-analysis

We present dichotomous outcomes (50% reduction in monthly migraine days and adverse events leading to discontinuation) number of events per 1,000 patients, compared to placebo. For example, among 1,000 patients using fremanezumab for migraine, 341 more patients will experience a 50% or more reduction in monthly migraine days, compared to 1,000 patients using placebo. To calculate absolute effects for 50% or more reduction in monthly migraine days, we estimated a baseline risk (i.e., the risk of experiencing a 50% or more reduction in monthly migraine days with placebo) by calculating the median risk across placebo arms across all trials. We subsequently used the baseline risk and the relative risk to calculate a risk difference. We present monthly migraine days as mean difference in migraine days and associated confidence intervals, compared to placebo. For example, fremanezumab results in an average of 2·22 fewer monthly migraine days, compared to placebo. The panel on the right presents the direction of effects, GRADE ratings, and their interpretation. High certainty evidence indicates situations in which we have high certainty that the true effect lies close to estimated effect and low or very low certainty evidence indicates situations in which the true effect may be substantially different from the estimated effect. For example, results in dark green suggest high certainty evidence that a drug is better than placebo whereas results in dark red suggest high certainty evidence that the drug is more harmful than placebo

* downgraded due to risk of bias

† downgraded due to imprecision

‡ downgraded due to inconsistency

*MCID* minimal clinically important difference; *MID *The minimal important difference

We classified drugs into the following nodes, regardless of dose: beta-blockers, calcium channel blockers, gepants, gabapentin/pregabalin, topiramate, valproate, amitriptyline, carisbamate, and oxcarbazepine. We also included each of the CGRP(r)mAbs as separate nodes, to facilitate comparisons between them. We grouped beta-blockers, calcium channel blockers, gepants, and gabapentin/pregabalin because we anticipated similar efficacy and safety

Fremanezumab appeared the most beneficial, with high certainty evidence that it increases the proportion of patients who experience a 50% or more reduction in monthly migraine days compared to gepants, topiramate, and carisbamate. Fremanezumab shows moderate certainty of superiority compared to amitriptyline, beta-blockers, calcium channel blockers, oxcarbezapine, galcanezumab, eptinezumab, erenumab, and valproate and low certainty evidence compared to gabapentin.

Supplements 8 and 9 present pairwise meta-analyses and node split plots, respectively.

We performed four additional secondary analyses. In the first secondary analysis, we grouped all CGRP(r)mAbs in one node assuming that they all produce similar effects. In the second, we restricted trials to those that investigated recommended therapeutic doses of drugs. In the third, we classified each gepant as a separate node, assuming that their effects may be different from each other. Finally, we performed a sensitivity analysis excluding telcagepant. These secondary analyses produced results consistent with the primary analysis. In all analyses, CGRP(r)mAbs and gepants appeared the most effective at increasing the proportion of patients who experience a 50% or more reduction in monthly migraine days (Supplement 10 to 13). We did not find the effects of gepants to be different from one another, though the effect estimates were more imprecise when each gepant was classified into a separate node. The effect of telcagepant was also consistent with other gepants.

We did not find convincing evidence that the effects of drugs vary based risk of bias, baseline monthly migraine days, or the proportion of patients who had previously used prophylactic drugs (Supplement 14).

### Monthly migraine/headache days

Sixty-two trials, including 29,156 patients, reported on monthly migraine or monthly headache days [11, 36–41, 43, 44, 47, 48, 50–52, 54–61, 63–71, 74–78, 80, 81, 83–98, 100–106]. Supplement 15 presents the network geometry and Supplement 16 presents results and GRADE ratings all comparisons. We found high certainty evidence that, compared to placebo, fremanezumab, erenumab, galcanezumab, eptinezumab, gepants, topiramate, and beta-blockers reduce monthly migraine days, and that oxcarbazepine and gabapentin are not different from placebo. We also found moderate certainty evidence that valproate, amitriptyline, and calcium channel blockers are probably not different from placebo. Supplements 17 and 18 present pairwise meta-analyses and node split plots, respectively.

### Adverse events leading to discontinuation

Sixty-six trials, including 29,327 patients, reported adverse events leading to discontinuation [11, 36–42, 44–52, 54–61, 63–65, 69–93, 95–98, 100–107]. Supplement 19 presents the network geometry and Supplement 20 presents results and GRADE ratings all comparisons. We found high certainty evidence that valproate and amitriptyline result in more adverse events leading to discontinuation, compared to placebo, and that erenumab is not different than placebo. We found moderate certainty evidence that topiramate, beta-blockers, oxcarbazepine, and gabapentin probably result in more adverse events and that frenanezumab, galcanezumab, eptinezumab, gepants, and carisbamate are probably not different from the trials on these drugs placebo. We found low certainty evidence that calcium channel blockers may increase adverse events compared with placebo. Supplements 21 and 22 present the pairwise meta-analyses and node split plots. We performed sensitivity analyses in which we grouped all CGRP(r)mAbs in one node, restricted trials to those that investigated recommended therapeutic doses of drugs and grouped each gepant in a separate node (Supplements 23 to 25). These sensitivity analyses produced results consistent with the primary analysis with CGRP(r)mAbs and gepants being associated with the fewest adverse events. We also did not find gepants to result in different degrees of adverse events, though the effect estimates were more imprecise when each gepant was classified into a separate node (Supplement 26). We also did not find convincing evidence that adverse events leading to discontinuation varies based on risk of bias, baseline monthly migraine days, or the proportion of patients who had previously used prophylactic drugs (Supplement 27).

## Discussion

### Main findings

We present a systematic review and network meta-analysis, including 74 trials and 32,990 patients addressing the efficacy and safety of drugs used for migraine prophylaxis, including head-to-head comparisons between drugs that have not yet been compared in clinical trials. We show that CGRP(r)mAbs have the highest efficacy and the lowest incidence of adverse events compared to placebo, closely followed by gepants. We also show that commonly used drugs, like amitriptyline, beta-blockers, and topiramate, appear not only be less effective than CGRP(r)mAbs) and gepants, but they are associated with substantially higher risk of adverse events—an important issue since more than half of patients discontinue prophylactic migraine drugs within 6 months, attributed to poor efficacy and tolerability [109].

### Strengths and limitations

The strengths of our review include a comprehensive search strategy, duplicate screening and data extraction, and rigorous assessment of the certainty of evidence using the latest GRADE guidance [33, 34]. We also focus on patient-important outcomes, informed by an established core outcome set [21]. Despite our rigorous literature search, it is possible that we missed eligible trials. We mitigated this limitation by also reviewing the references of similar systematic reviews and soliciting experts about eligible trials that may not have come up in our search [6–9, 15–17]. We anticipate that evidence users, such as clinicians, may be concerned about heterogeneity and the appropriateness of pooling results across trials. It is reassuring, however, that we did not identify substantial heterogeneity. In fact, we found relative consistency in the effects reported across trials investigating the same drugs, despite differences in eligibility criteria. We assessed the certainty of evidence using the GRADE approach [110]. While the GRADE approach presents a comprehensive framework for considering all factors that may bear on the certainty of evidence, its application is ultimately subjective, and others may come to different conclusions about the certainty of evidence.

Our systematic review did not assess function, disability, or quality of life outcomes—primarily due to disparate measures used in trials, particularly older versus newer trials. We anticipate, however, these outcomes to be strongly correlated with migraine days and adverse events [6, 111].

Although we planned to assess migraine pain/intensity, as specified in our protocol, this outcome was only reported in four trials, precluding analysis. We were also unable to perform subgroup analyses based on medication overuse headache, due to lack of reporting in trials, and hence our results may not be applicable to patients with medication overuse headache. Our review also does not include certain drugs that were also investigated for migraine prophylaxis e.g. an angiotensin II type 1 receptor antagonist [112]. since these trials on these drugs did not meet our eligibility criteria due to the limited sample size. Further, we did not include botulinum toxin in the network, due to heterogeneity and evidence that it has different effects in episodic and chronic migraine [18, 19].

Trials often used run-in periods to assess compliance with headache diaries and excluded patients with suboptimal adherence or completion of headache diaries. Thus, patients included in trials may have been more compliant, which may have translated to their adherence to study drugs. The effects of drugs may be more modest in patients with suboptimal compliance.

To facilitate interpretation, we converted the relative risk of experiencing a 50% or more reduction in monthly migraine days to the number of patients with the outcome among 1,000 patients, using the median risk of experiencing a 50% or more reduction in monthly migraine days across trials in the placebo arm. We acknowledge, however, that injectable placebos may produce stronger placebo responses compared to oral placebos. The relative effects presented will not be affected by this issue, since trials investigating oral drugs use oral placebos and trials investigating injectable drugs use injectable placebos. Evidence users who are concerned about the applicability of the placebo response in their context may calculate absolute effects using different estimates of response for placebo (i.e., absolute risk = RR x risk in placebo group).

We categorized different doses of the same intervention in the same node. Although this maximized the number of patients in each node, the effects of drugs may vary based on dose. To address this limitation, we performed a sensitivity analysis where we restricted trials to those that investigated therapeutic doses of interventions, which produced results consistent with the main analysis.

Our results are limited by the duration of follow-up in trials. Trials reported outcomes between 12 and 52 weeks and the effects of these drugs beyond 52 weeks from randomized trials is unclear.

Old trials generally failed to distinguish between episodic and chronic migraine while newer trials, which typically investigated CGRP(r)mAbs and gepants, distinguished between episodic and chronic migraine. It is possible that the effects of drugs may be different based on episodic or chronic migraine or whether patients had previously been treated by other prophylactics. We performed subgroup analyses investigating the effects of drugs based on baseline monthly migraine days and the proportion who were treated by other prophylactics, and we did not find evidence that the effects of drugs are different based on these factors.

There was heterogeneity in how trials defined a 50% reduction in monthly migraine days, with some trials requiring a reduction in monthly migraine days in the last four weeks of the trial compared to the baseline and others requiring a sustained response over several months. Select trials also reported on reduction in migraine frequency or attacks. We anticipate, however, the relative effect between trial arms to be similar, regardless of the definition of 50% reduction in monthly migraine days or migraine frequency.

While our review reports on adverse events that led to discontinuation, we did not synthesize data on serious and life-threatening adverse events.

## Implications

Our results suggest that CGRP(r)mAbs and gepants are the most effective and better tolerated drugs for migraine prophylaxis. However, different international guidelines and national reimbursement policies only support these drugs for patients who have not responded to other prophylactic drugs. Among the oral prophylactics, high dropout rates were reported especially for amitriptyline, topiramate, or valproate [113]. These characteristics lead most individuals with migraine to express a clear preference for CGRP(r)mAbs as a first-line option [114]. Our results scientifically support this patient’s preference. It is worth mentioning that oral drugs may be preferrable as first migraine preventive options in patients with different co-morbidities or in countries with lack of availability of CGRP(r)mAbs or gepants. Current guidelines on optimal migraine prophylaxis also do not provide guidance on which drugs are most effective or a hierarchy to inform clinicians and patients in selecting drugs, due to the lack of head-to-head comparisons [10, 115]. Our systematic review and network meta-analysis addresses this unmet need and may be relevant in drafting future guidelines.

## Conclusion

CGRP(r)mAbs are the most effective and tolerated treatment for migraine prophylaxis, followed closely by gepants. Commonly used older classes of drugs appear to not only be less effective than CGRP(r)mAbs and gepants, but they are also associated with substantially higher risk of adverse events.

## Supplementary Information

**Additional file 1:** **Supplement 1.** Search strategy. **Supplement 2.** Risk of bias criteria. **Supplement 3.** Sensitivity analysis restricted to recommended therapeutic doses of drugs. **Supplement 4.** Table of trial characteristics. **Supplement 5.** Risk of bias judgements for mean monthly migraine days. **Supplement 6.** Risk of bias judgements for adverse events leading to discontinuation. **Supplement 7.** Comparisons and GRADE ratings for network meta-analysis of 50% or more reduction in monthly migraine days. **Supplement 8.** Pairwise meta-analyses for 50% or more reduction in monthly migraine days. **Supplement 9.** Node split plots for 50% or more reduction in monthly migraine days. **Supplement 10.** Secondary analysis for 50% or more reduction in monthly migraine days. **Supplement 11.** Secondary analysis for 50% or more reduction in monthly migraine days. **Supplement 12.** Secondary analysis for 50% or more reduction in monthly migraine days. **Supplement 13.** Secondary analysis comparing the effects of telcagepant with other gepants. **Supplement 14.** Subgroup analyses for 50% or more reduction in monthly migraine days. **Supplement 15.** Network diagram for monthly migraine days. **Supplement 16.** Comparisons and GRADE ratings for network meta-analysis of monthly migraine days. **Supplement 17.** Pairwise meta-analyses for monthly migraine days. **Supplement 18.** Node split plots for monthly migraine days. **Supplement 19.** Network diagram for adverse events leading to discontinuation. **Supplement 20.** Comparisons and GRADE ratings for network meta-analysis of adverse events leading to discontinuation. **Supplement 21.** Pairwise meta-analyses for adverse events leading to discontinuation. **Supplement 22.** Node split plots for adverse events leading to discontinuation. **Supplement 23.** Secondary analysis for adverse events leading to discontinuation. **Supplement 24.** Secondary analysis for adverse events leading to discontinuation. **Supplement 25.** Secondary analysis for adverse events leading to discontinuation. **Supplement 26.** Secondary analysis comparing the effects of telcagepant with other gepnts. **Supplement 27.** Subgroup analyses for adverse events leading to discontinuation.

## Abbreviations

CGRP(r)mAbs: Calcitonin gene-related peptide (receptor) monoclonal antibodies
PRISMA: Preferred Reporting Items for Systematic Reviews
COSMIG: Outcome set for preventive intervention trials in chronic and episodic migraine
REML: Restricted maximum likelihood
RR: Relative Risk
MDs: Mean differences
RDs: Risk differences
*MCID*: Minimal clinically important difference
MID: The minimal important difference
