# Supplementary material for: The comparative effectiveness of migraine preventive drugs: a systematic review and network meta-analysis
Source: J Headache Pain. 2023 May 19;24(1):56. doi: 10.1186/s10194-023-01594-1 (PMC10197489; doi:10.1186/s10194-023-01594-1)
Supplement: Supplementary file 1 — Additional file 1: Supplement 1. Search strategy. Supplement 2. Risk of bias criteria. Supplement 3. Sensitivity analysis restricted to recommended therapeutic doses of drugs. Supplement 4. Table of trial characteristics. Supplement 5. Risk of bias judgements for mean monthly migraine days. Supplement 6. Risk of bias judgements for adverse events leading to discontinuation. Supplement 7. Comparisons and GRADE ratings for network meta-analysis of 50% or more reduction in monthly migraine days. Supplement 8. Pairwise meta-analyses for 50% or more reduction in monthly migraine days. Supplement 9. Node split plots for 50% or more reduction in monthly migraine days. Supplement 10. Secondary analysis for 50% or more reduction in monthly migraine days. Supplement 11. Secondary analysis for 50% or more reduction in monthly migraine days. Supplement 12. Secondary analysis for 50% or more reduction in monthly migraine days. Supplement 13. Secondary analysis comparing the effects of telcagepant with other gepants. Supplement 14. Subgroup analyses for 50% or more reduction in monthly migraine days. Supplement 15. Network diagram for monthly migraine days. Supplement 16. Comparisons and GRADE ratings for network meta-analysis of monthly migraine days. Supplement 17. Pairwise meta-analyses for monthly migraine days. Supplement 18. Node split plots for monthly migraine days. Supplement 19. Network diagram for adverse events leading to discontinuation. Supplement 20. Comparisons and GRADE ratings for network meta-analysis of adverse events leading to discontinuation. Supplement 21. Pairwise meta-analyses for adverse events leading to discontinuation. Supplement 22. Node split plots for adverse events leading to discontinuation. Supplement 23. Secondary analysis for adverse events leading to discontinuation. Supplement 24. Secondary analysis for adverse events leading to discontinuation. Supplement 25. Secondary analysis for adverse events leading to discontinuation. Supplement 26. [file 10194_2023_1594_MOESM1_ESM.docx]

# The comparative effectiveness of drugs for migraine prevention: a systematic review and network meta-analysis

# Table of Contents

[The comparative effectiveness of drugs for migraine prevention: a systematic review and network meta-analysis 1](#_Toc124361903)

[Table of Contents 2](#_Toc124361904)

[Supplement 1 – Search strategy 3](#_Toc124361905)

[Supplement 2 – Risk of bias criteria 16](#_Toc124361906)

[Supplement 3 – Sensitivity analysis restricted to recommended therapeutic doses of drugs 19](#_Toc124361907)

[Supplement 4 – Table of trial characteristics 20](#_Toc124361908)

[Supplement 5 – Comparisons and GRADE ratings for network meta-analysis of 50% or more reduction in monthly migraine days 31](#_Toc124361909)

[Supplement 6 – Pairwise meta-analyses for 50% or more reduction in monthly migraine days 33](#_Toc124361910)

[Supplement 7 – Node split plots for 50% or more reduction in monthly migraine days 34](#_Toc124361911)

[Supplement 8 – Secondary analysis for 50% or more reduction in monthly migraine days (all monoclonal antibodies are grouped in one node) 36](#_Toc124361912)

[Supplement 9 – Secondary analysis for 50% or more reduction in monthly migraine days (restricted trials to those that investigated recommended therapeutic doses of drugs) 37](#_Toc124361913)

[Supplement 10 – Subgroup analyses for 50% or more reduction in monthly migraine days 38](#_Toc124361914)

[Supplement 11 – Network diagram for monthly migraine days 39](#_Toc124361915)

[Supplement 12 – Comparisons and GRADE ratings for network meta-analysis of monthly migraine days 40](#_Toc124361916)

[Supplement 13 – Pairwise meta-analyses for monthly migraine days 41](#_Toc124361917)

[Supplement 14 – Node split plots for monthly migraine days 42](#_Toc124361918)

[Supplement 15 – Network diagram for adverse events leading to discontinuation 43](#_Toc124361919)

[Supplement 16 – Comparisons and GRADE ratings for network meta-analysis of adverse events leading to discontinuation 44](#_Toc124361920)

[Supplement 17 – Pairwise meta-analyses for adverse events leading to discontinuation 45](#_Toc124361921)

[Supplement 18 – Node split plots for adverse events leading to discontinuation 46](#_Toc124361922)

[Supplement 19 – Secondary analysis for adverse events leading to discontinuation (all monoclonal antibodies are grouped in one node) 47](#_Toc124361923)

[Supplement 20 – Secondary analysis for adverse events leading to discontinuation (restricted trials to those that investigated recommended therapeutic doses of drugs) 48](#_Toc124361924)

# Supplement 1 – Search strategy

**Search Strategies**

**PRISMA Initial Results:** total number of results before duplicates removed

| **Database [Platform]** Searches run August 13, 2022. *No date, language limits used.* | **Results** |
| --- | --- |
| OVID Medline Epub Ahead of Print, In-Process & Other Non-Indexed Citations, Ovid MEDLINE(R) Daily and Ovid MEDLINE(R) 1946 to Present [Ovid] | 5,913 |
| Embase 1974 to 2022 August 12 [Ovid] | 5,983 |
| EBM Reviews - Cochrane Central Register of Controlled Trials July 2022 [Ovid] | 3,267 |
| **TOTAL** | **15,163** |

**ClinicalTrials.gov Results** Searched August 13, 2022.

| Studies With Results \| Interventional Studies \| Migraine Disorders \| various treatment terms [see strategies below in document] | 94 |
| --- | --- |
| **TOTAL** | **94** |

**ClinicalTrials.gov Search Strategies**

1 Study found for: CGRP monoclonal antibodies | Studies With Results | Interventional Studies | Migraine Disorders

8 Studies found for: adrenergic beta-antagonists | Studies With Results | Interventional Studies | Migraine Disorders

1 Study found for: Calcium Channel Blocker | Studies With Results | Interventional Studies | Migraine Disorders

22 Studies found for: Anticonvulsants | Studies With Results | Interventional Studies | Migraine Disorders

39 Studies found for: Anti-Inflammatory Agents, Non-Steroidal | Studies With Results | Interventional Studies | Migraine Disorders

1 Study found for: angiotensin receptor antagonists | Studies With Results | Interventional Studies | Migraine Disorders

5 Studies found for: antidepressive agents | Studies With Results | Interventional Studies | Migraine Disorders

11 Studies found for: Botulinum Toxins, Type A | Studies With Results | Interventional Studies | Migraine Disorders

No Studies found for: coenzyme Q10 | Studies With Results | Interventional Studies | Migraine Disorders

3 Studies found for: Magnesium | Studies With Results | Interventional Studies | Migraine Disorders

3 Studies found for: Melatonin | Studies With Results | Interventional Studies | Migraine Disorders

**MEDLINE(R)**1996 to August 12, 2022
Search Strategy:

| **#** | **Searches** | **Results** |
| --- | --- | --- |
| 1 | exp Migraine Disorders/ | 22110 |
| 2 | (migraine or migraines or sick headache or sick headaches or hemicrania or migrainous headache or status hemicranicus).tw,kf. | 28623 |
| 3 | or/1-2 | 30802 |
| 4 | ("cgrp monoclonal antibodies" or "calcitonin gene-related peptide antibodies (cgrp) monoclonal antibodies (mabs)" or "cgrp mabs").tw,kf. | 127 |
| 5 | calcitonin gene related peptide.tw,kf. | 8169 |
| 6 | Calcitonin Gene-Related Peptide Receptor Antagonists/ | 848 |
| 7 | exp Antibodies, Monoclonal/ | 191819 |
| 8 | 6 and 7 | 274 |
| 9 | adrenergic beta-antagonists/ | 24650 |
| 10 | ("adrenergic beta antagonist*" or "adrenergic beta blocker*" or "adrenergic beta receptor blockader*" or "beta adrenergic antagonist*" or "beta adrenergic blocker*" or "beta adrenergic blocking agent*" or "beta adrenergic blocking drug" or "beta adrenergic receptor antagonist*" or "beta adrenergic receptor blockader*" or "beta adrenergic receptor blocker*" or "beta adrenoceptor antagonist*" or "beta adrenoceptor blocker*" or "beta adrenoceptor blocking agent*" or "beta adrenoceptor blocking drug*" or "beta adrenolytic" or "beta antagonist*" or "beta antiadrenergic agent*" or "beta blocker" or "beta blocking adrenergic agent*" or "beta blocking agent*" or "beta blocking drug*" or "beta receptor adrenergic blocking agent*" or "beta receptor blocker*" or "beta receptor blocking agent*" or "beta sympathicolytic agent*" or "beta sympathicolytics" or "beta sympatholytic agent*" or "betasympatholytic agent*").tw,kf. | 14206 |
| 11 | alprenolol/ or brimonidine tartrate, timolol maleate drug combination/ or bunolol/ or bupranolol/ or carteolol/ or carvedilol/ or dihydroalprenolol/ or iodocyanopindolol/ or labetalol/ or levobunolol/ or metipranolol/ or nadolol/ or oxprenolol/ or penbutolol/ or pindolol/ or propranolol/ or sotalol/ or timolol/ | 14502 |
| 12 | (alprenolol or bucindolol or bunolol or bupranolol or carazolol or carpindolol or carteolol or carvedilol or cloranolol or dexpropranolol or dichlorisoprenaline or dihydroalprenolol or dilevalol or iodocyanopindolol or isamoltane or labetalol or levobunolol or metipranolol or nadolol or nifenalol or oxprenolol or penbutolol or pindolol or propranolol or sotalol or spirendolol or tertatolol or tilisolol or timolol).tw,kf. | 19291 |
| 13 | adrenergic beta-1 receptor antagonists/ | 1281 |
| 14 | acebutolol/ or atenolol/ or betaxolol/ or bisoprolol/ or celiprolol/ or metoprolol/ or practolol/ | 6735 |
| 15 | (acebutolol or atenolol or bendacalol or betaxolol or bevantolol or bisoprolol or celiprolol or cetamolol or cyanoiodopindolol or cyanopindolol or dramedilol or epanolol or esmolol or flusoxolol or landiolol or metoprolol or nebivolol or practolol or propranolol or ritodrine or salcardolol or sandoz 204545 or sotalol or talinolol or vortioxetine).tw,kf. | 22643 |
| 16 | Calcium Channel Blockers/ | 25268 |
| 17 | ("calcium blocker" or "calcium blocking agent*" or "calcium channel antagonist*" or "calcium channel blocker*" or "calcium channel blocking drug*" or "calcium entry blocker" or "calcium entry blocking agent*" or "calcium inhibitor" or "exogenous calcium antagonist*" or "exogenous calcium blockader*").tw,kf. | 12479 |
| 18 | Anticonvulsants/ | 40862 |
| 19 | ("anti convulsant agent*" or "anti convulsive agent*" or "anti convulsive drug*" or "anti epileptic*" or "anticonvulsant*" or "anticonvulsive agent*" or "anticonvulsive drug*" or "anticonvulsivum" or "antiepileptic" or "antiepileptics" or "antiepileptiform drug*").tw,kf. | 34720 |
| 20 | acetazolamide/ or bromides/ or cannabidiol/ or carbamazepine/ or chlormethiazole/ or clobazam/ or clonazepam/ or clorazepate dipotassium/ or diazepam/ or dimethadione/ or estazolam/ or ethosuximide/ or felbamate/ or flunarizine/ or gabapentin/ or lacosamide/ or lamotrigine/ or levetiracetam/ or lorazepam/ or magnesium sulfate/ or medazepam/ or mephenytoin/ or mephobarbital/ or meprobamate/ or nitrazepam/ or oxcarbazepine/ or paraldehyde/ or phenobarbital/ or phenytoin/ or pregabalin/ or primidone/ or riluzole/ or thiopental/ or tiagabine/ or tiletamine/ or topiramate/ or trimethadione/ or valproic acid/ or vigabatrin/ or zonisamide/ | 57455 |
| 21 | (acetazolamide or albutoin or alprazolam or ascorbate magnesium or belnacasan or brexanolone or brivaracetam or bromides or cannabidiol or cannabidivarin or carabersat or carbamazepine or carisbamate or cenobamate or chlormethiazole or clobazam or clomethiazole or clonazepam or clorazepate dipotassium or darigabat or dextromethorphan or dezinamide or diazepam or diclofenamide or dimethadione or elpetrigine or estazolam or eterobarb or ethosuximide or ethotoin or etiracetam or felbamate or fenfluramine or flunarizine or gabapentin or ganaxolone or guaifenesin or "ica 105665" or imepitoin or imidazenil or lacosamide or lamotrigine or lanicemine or levetiracetam or licarbazepine or lorazepam or loreclezole or losigamone or magnesium sulfate or medazepam or mephenytoin or mephobarbital or meprobamate or mesuximide or metharbital or midazolam or nitrazepam or oxcarbazepine or padsevonil or paraldehyde or paramethadione or pentoxyverine or perampanel or phenobarbital or phenytoin or pregabalin or primidone or progabide or remacemide or retigabine or riluzole or ropizine or rufinamide or safinamide or seletracetam or sodium bromide or stiripentol or sultiame or talampanel or thiopental or tiagabine or tiletamine or topiramate or trimethadione or valproic acid or valpromide or valrocemide or vigabatrin or vixotrigine or zaleplon or zonisamide).tw,kf. | 82787 |
| 22 | exp Anti-Inflammatory Agents, Non-Steroidal/ | 138244 |
| 23 | ("anti inflammatory analgesic*" or "aspirin like agent*" or "non steroid antiinflammatory agent*" or "non steroid antiinflammatory drug*" or "non steroidal anti inflammatory agent*" or "non steroidal anti inflammatory drug*" or "non steroidal antiinflammatory agent*" or "non steroidal antiinflammatory drug*" or "nonsteroid antiinflammatory agent*" or "nonsteroid antiinflammatory drug" or "nonsteroid antirheumatic agent" or "nonsteroidal anti inflammatory agent*" or "nonsteroidal anti inflammatory drug*" or "nonsteroidal antiinflammatory agent*" or "nonsteroidal antiinflammatory drug*" or "nsaid" or "nsaids").tw,kf. | 36916 |
| 24 | Acetaminophen/ or Antipyrine/ or Aspirin/ or Celecoxib/ or Clonixin/ or Curcumin/ or Diclofenac/ or dipyrone/ or Ibuprofen/ or Indomethacin/ or Ketoprofen/ or Ketorolac/ or Ketorolac Tromethamine/ or Mesalamine/ or Naproxen/ or salicylates/ or Sulfasalazine/ or Tolmetin/ | 96692 |
| 25 | (acalabrutinib or aceclofenac or acemetacin or acetaminophen or acetaminosalol or acetylsalicylic acid or actarit or adalimumab or alemtuzumab or antipyrine or apremilast or ascriptin or aspirin or azathioprine or azelaic acid or balsalazide or belimumab or brimonidine or celecoxib or clonixin lysine or clonixin or curcumin or dexibuprofen or dexketoprofen or diclofenac or dipyrone or etodolac or etoricoxib or fenoprofen or flurbiprofen or ibuprofen or icosapentaenoic acid or indometacin or indomethacin or ketoprofen or ketorolac or leflunomide or lornoxicam or loxoprofen or lumiracoxib or meclofenamic acid or mefenamic acid or meloxicam or mesalamine or mesalazine or methotrexate or nabumetone or naproxen or natalizumab or nimesulide or parecoxib or phenylbutazone or piroxicam or pirprofen or rasagiline or rituximab or rofecoxib or ruxolitinib or salazosulfapyridine or salicylates or salicylic acid or satralizumab or sulfasalazine or sulindac or tenoxicam or teriflunomide or tofacitinib or tolmetin or valdecoxib).tw,kf. | 203782 |
| 26 | exp Angiotensin Receptor Antagonists/ | 24152 |
| 27 | ("angiotensin ii receptor antagonist*" or "angiotensin ii receptor blocker*" or "angiotensin ii receptor blocking agent*" or "angiotensin receptor antagonist" or "angiotensin receptor blocker*" or "angiotensin receptor blocking agent*").tw,kf. | 12160 |
| 28 | irbesartan/ or olmesartan medoxomil/ or telmisartan/ or valsartan/ | 6612 |
| 29 | ("angiotensin 1 receptor antagonist" or "angiotensin 2 receptor antagonist" or azilsartan or candesartan or eprosartan or fimasartan or irbesartan or losartan or olmesartan or sartan derivative or tasosartan or telmisartan or trv027 or valsartan).tw,kf. | 17916 |
| 30 | exp Antidepressive Agents/ | 94901 |
| 31 | ("anti depressant agent*" or "antidepressant*" or "antidepression drug*" or "antidepressive agent*" or "antidepressive drug*" or "neurothymoleptic agent*" or "psychoenergizer" or "thymoanaleptic" or "thymoanaleptics" or thymoleptic or thymoleptics or "thymolytic agent").tw,kf. | 52365 |
| 32 | Aripiprazole/ or Duloxetine Hydrochloride/ or Lithium Carbonate/ or Lithium Compounds/ or Mirtazapine/ or Moclobemide/ or Phenelzine/ or Pizotyline/ or Quetiapine Fumarate/ or Sertraline/ or Tranylcypromine/ | 16745 |
| 33 | Bupropion/ or Citalopram/ or Fluoxetine/ or Mianserin/ or Paroxetine/ or Sulpiride/ or Trazodone/ or Tryptophan/ or Venlafaxine Hydrochloride/ | 38437 |
| 34 | Amitriptyline/ or Clomipramine/ or Desipramine/ or Dothiepin/ or Doxepin/ or Imipramine/ or Nortriptyline/ or Opipramol/ | 7959 |
| 35 | (agomelatine or amitriptyline or aprepitant or aripiprazole or asenapine or "botulinum toxin a" or bupropion or citalopram or clomipramine or desipramine or dothiepin or doxepin or duloxetine hydrochloride or esketamine or fluoxetine or gepirone or imipramine or indalpine or ipsapirone or lithium acetate or lithium carbonate or lithium chloride or lithium compounds or lithium salt or mianserin or mifepristone or minaprine or mirtazapine or moclobemide or monoamine oxidase inhibitor* or noradrenalin uptake inhibitor* or nortriptyline or opipramol or paroxetine or phenelzine or pizotyline or quetiapine fumarate or serotonin uptake inhibitor* or sertraline or sulpiride or tetracyclic antidepressant* or tranylcypromine or trazodone or tricyclic antidepressant* or tryptophan or venlafaxine hydrochloride).tw,kf. | 84436 |
| 36 | Botulinum Toxins, Type A/ | 10631 |
| 37 | ("abobotulinum toxin a" or "abobotulinumtoxin a" or "abobotulinumtoxina" or "agn 151607" or "agn151607" or "alluzience" or "ant 1207" or "ant 1401" or "ant 1403" or "ant1207" or "ant1401" or "ant1403" or "azzalure" or "bocouture" or "boe−tox" or "bont a" or "bont serotype a" or "botox" or "botulin a" or "botulin toxin a" or "botulinium a toxin" or "botulinum a exotoxin" or "botulinum a toxin" or "botulinum neurotoxin a" or "botulinum toxin a" or "btxa" or "clostridium botulinum a toxin" or "clostridium botulinum endotoxin" or "clostridium botulinum neurotoxin a" or "clostridium botulinum toxin type a" or "clostridium botulinum type a neurotoxin" or "cnt 52120" or "cnt52120" or "daxibotulinum toxin a" or "daxibotulinumtoxin a" or "daxibotulinumtoxina" or "dwp 450" or "dwp450" or "dyslor" or "dysport" or "evabotulinum toxin a" or "evabotulinumtoxin a" or "evabotulinumtoxina" or "evosyal" or "gemibotulinum toxin a" or "gemibotulinumtoxin a" or "gemibotulinumtoxina" or "gsk 1358820" or "gsk1358820" or "incobotulinum toxin a" or "incobotulinumtoxin a" or "incobotulinumtoxina" or "ipn 59011" or "ipn59011" or "jeuveau" or "letibotulinum toxin a" or "letibotulinumtoxin a" or "letibotulinumtoxina" or "meditoxin" or "mt 10109" or "mt10109" or "nabota" or "neuronox" or "nivobotulinum toxin a" or "nivobotulinumtoxin a" or "nivobotulinumtoxina" or "nt 201" or "nt201" or "nuceiva" or "oculinum" or "onabotulinum toxin a" or "onabotulinumtoxin a" or "onabotulinumtoxina" or "onaclostox" or "pm 12759" or "pm12759" or "prabotulinum toxin a" or "prabotulinumtoxin a" or "prabotulinumtoxina" or "prosigne" or "purtox" or "qm 1114" or "qm1114" or "relabotulinum toxin a" or "relabotulinumtoxin a" or "relabotulinumtoxina" or "reloxin" or "rtt 150" or "rtt150" or "vistabel" or "vistabex" or "xeomeen" or "xeomin").tw,kf. | 7610 |
| 38 | (caomet or "coenzyme 910" or "coenzyme q 10" or "coenzyme Q10" or decorenone or mitocor or neuquinone or "quinone q 10" or ubidecarenone or ubimaior or "ubiquinone (10)" or "ubiquinone 10" or "ubiquinone 50" or ubiten).tw,kf. | 4064 |
| 39 | Magnesium/ | 24624 |
| 40 | (magnesium or romag).tw,kf. | 32593 |
| 41 | Melatonin/ | 17282 |
| 42 | ("apl 510" or "apl510" or ceyesto or circadin or "jan 13004" or "jan13004" or "ki 1001" or "ki1001" or melatonin or melatonina or melovine or orlogin or slenyto or "sp 13004" or "sp13004" or waferest).tw,kf. | 20405 |
| 43 | or/4-5,8-42 | 696356 |
| 44 | 3 and 43 [migraines AND prophylaxis] | 6793 |
| 45 | randomized controlled trial.pt. | 480050 |
| 46 | controlled clinical trial.pt. | 49673 |
| 47 | randomized.ab. | 460234 |
| 48 | placebo.ab. | 169835 |
| 49 | drug therapy.fs. | 1880993 |
| 50 | randomly.ab. | 294766 |
| 51 | trial.ab. | 482925 |
| 52 | groups.ab. | 1714791 |
| 53 | 45 or 46 or 47 or 48 or 49 or 50 or 51 or 52 | 3949647 |
| 54 | animals/ not humans.sh. | 2817192 |
| 55 | 53 not 54 | 3422601 |
| 56 | 44 and 55 [migraines AND prophylaxis AND RCTs] | 4566 |
| 57 | remove duplicates from 56 | 4540 |

 OVID Medline Epub Ahead of Print, In-Process & Other Non-Indexed Citations, Ovid MEDLINE(R) Daily and Ovid MEDLINE(R) 1946 to Present
Search Strategy:

| **#** | **Searches** | **Results** |
| --- | --- | --- |
| 1 | exp Migraine Disorders/ | 30669 |
| 2 | (migraine or migraines or sick headache or sick headaches or hemicrania or migrainous headache or status hemicranicus).tw,kf. | 40663 |
| 3 | or/1-2 | 44880 |
| 4 | ("cgrp monoclonal antibodies" or "calcitonin gene-related peptide antibodies (cgrp) monoclonal antibodies (mabs)" or "cgrp mabs").tw,kf. | 167 |
| 5 | calcitonin gene related peptide.tw,kf. | 12349 |
| 6 | Calcitonin Gene-Related Peptide Receptor Antagonists/ | 869 |
| 7 | exp Antibodies, Monoclonal/ | 267646 |
| 8 | 6 and 7 | 274 |
| 9 | adrenergic beta-antagonists/ | 41510 |
| 10 | ("adrenergic beta antagonist*" or "adrenergic beta blocker*" or "adrenergic beta receptor blockader*" or "beta adrenergic antagonist*" or "beta adrenergic blocker*" or "beta adrenergic blocking agent*" or "beta adrenergic blocking drug" or "beta adrenergic receptor antagonist*" or "beta adrenergic receptor blockader*" or "beta adrenergic receptor blocker*" or "beta adrenoceptor antagonist*" or "beta adrenoceptor blocker*" or "beta adrenoceptor blocking agent*" or "beta adrenoceptor blocking drug*" or "beta adrenolytic" or "beta antagonist*" or "beta antiadrenergic agent*" or "beta blocker" or "beta blocking adrenergic agent*" or "beta blocking agent*" or "beta blocking drug*" or "beta receptor adrenergic blocking agent*" or "beta receptor blocker*" or "beta receptor blocking agent*" or "beta sympathicolytic agent*" or "beta sympathicolytics" or "beta sympatholytic agent*" or "betasympatholytic agent*").tw,kf. | 28363 |
| 11 | alprenolol/ or brimonidine tartrate, timolol maleate drug combination/ or bunolol/ or bupranolol/ or carteolol/ or carvedilol/ or dihydroalprenolol/ or iodocyanopindolol/ or labetalol/ or levobunolol/ or metipranolol/ or nadolol/ or oxprenolol/ or penbutolol/ or pindolol/ or propranolol/ or sotalol/ or timolol/ | 48975 |
| 12 | (alprenolol or bucindolol or bunolol or bupranolol or carazolol or carpindolol or carteolol or carvedilol or cloranolol or dexpropranolol or dichlorisoprenaline or dihydroalprenolol or dilevalol or iodocyanopindolol or isamoltane or labetalol or levobunolol or metipranolol or nadolol or nifenalol or oxprenolol or penbutolol or pindolol or propranolol or sotalol or spirendolol or tertatolol or tilisolol or timolol).tw,kf. | 51527 |
| 13 | adrenergic beta-1 receptor antagonists/ | 1328 |
| 14 | acebutolol/ or atenolol/ or betaxolol/ or bisoprolol/ or celiprolol/ or metoprolol/ or practolol/ | 14374 |
| 15 | (acebutolol or atenolol or bendacalol or betaxolol or bevantolol or bisoprolol or celiprolol or cetamolol or cyanoiodopindolol or cyanopindolol or dramedilol or epanolol or esmolol or flusoxolol or landiolol or metoprolol or nebivolol or practolol or propranolol or ritodrine or salcardolol or sandoz 204545 or sotalol or talinolol or vortioxetine).tw,kf. | 54958 |
| 16 | Calcium Channel Blockers/ | 37478 |
| 17 | ("calcium blocker" or "calcium blocking agent*" or "calcium channel antagonist*" or "calcium channel blocker*" or "calcium channel blocking drug*" or "calcium entry blocker" or "calcium entry blocking agent*" or "calcium inhibitor" or "exogenous calcium antagonist*" or "exogenous calcium blockader*").tw,kf. | 20506 |
| 18 | Anticonvulsants/ | 54979 |
| 19 | ("anti convulsant agent*" or "anti convulsive agent*" or "anti convulsive drug*" or "anti epileptic*" or "anticonvulsant*" or "anticonvulsive agent*" or "anticonvulsive drug*" or "anticonvulsivum" or "antiepileptic" or "antiepileptics" or "antiepileptiform drug*").tw,kf. | 52702 |
| 20 | acetazolamide/ or bromides/ or cannabidiol/ or carbamazepine/ or chlormethiazole/ or clobazam/ or clonazepam/ or clorazepate dipotassium/ or diazepam/ or dimethadione/ or estazolam/ or ethosuximide/ or felbamate/ or flunarizine/ or gabapentin/ or lacosamide/ or lamotrigine/ or levetiracetam/ or lorazepam/ or magnesium sulfate/ or medazepam/ or mephenytoin/ or mephobarbital/ or meprobamate/ or nitrazepam/ or oxcarbazepine/ or paraldehyde/ or phenobarbital/ or phenytoin/ or pregabalin/ or primidone/ or riluzole/ or thiopental/ or tiagabine/ or tiletamine/ or topiramate/ or trimethadione/ or valproic acid/ or vigabatrin/ or zonisamide/ | 121063 |
| 21 | (acetazolamide or albutoin or alprazolam or ascorbate magnesium or belnacasan or brexanolone or brivaracetam or bromides or cannabidiol or cannabidivarin or carabersat or carbamazepine or carisbamate or cenobamate or chlormethiazole or clobazam or clomethiazole or clonazepam or clorazepate dipotassium or darigabat or dextromethorphan or dezinamide or diazepam or diclofenamide or dimethadione or elpetrigine or estazolam or eterobarb or ethosuximide or ethotoin or etiracetam or felbamate or fenfluramine or flunarizine or gabapentin or ganaxolone or guaifenesin or "ica 105665" or imepitoin or imidazenil or lacosamide or lamotrigine or lanicemine or levetiracetam or licarbazepine or lorazepam or loreclezole or losigamone or magnesium sulfate or medazepam or mephenytoin or mephobarbital or meprobamate or mesuximide or metharbital or midazolam or nitrazepam or oxcarbazepine or padsevonil or paraldehyde or paramethadione or pentoxyverine or perampanel or phenobarbital or phenytoin or pregabalin or primidone or progabide or remacemide or retigabine or riluzole or ropizine or rufinamide or safinamide or seletracetam or sodium bromide or stiripentol or sultiame or talampanel or thiopental or tiagabine or tiletamine or topiramate or trimethadione or valproic acid or valpromide or valrocemide or vigabatrin or vixotrigine or zaleplon or zonisamide).tw,kf. | 148034 |
| 22 | exp Anti-Inflammatory Agents, Non-Steroidal/ | 211076 |
| 23 | ("anti inflammatory analgesic*" or "aspirin like agent*" or "non steroid antiinflammatory agent*" or "non steroid antiinflammatory drug*" or "non steroidal anti inflammatory agent*" or "non steroidal anti inflammatory drug*" or "non steroidal antiinflammatory agent*" or "non steroidal antiinflammatory drug*" or "nonsteroid antiinflammatory agent*" or "nonsteroid antiinflammatory drug" or "nonsteroid antirheumatic agent" or "nonsteroidal anti inflammatory agent*" or "nonsteroidal anti inflammatory drug*" or "nonsteroidal antiinflammatory agent*" or "nonsteroidal antiinflammatory drug*" or "nsaid" or "nsaids").tw,kf. | 51073 |
| 24 | Acetaminophen/ or Antipyrine/ or Aspirin/ or Celecoxib/ or Clonixin/ or Curcumin/ or Diclofenac/ or dipyrone/ or Ibuprofen/ or Indomethacin/ or Ketoprofen/ or Ketorolac/ or Ketorolac Tromethamine/ or Mesalamine/ or Naproxen/ or salicylates/ or Sulfasalazine/ or Tolmetin/ | 156025 |
| 25 | (acalabrutinib or aceclofenac or acemetacin or acetaminophen or acetaminosalol or acetylsalicylic acid or actarit or adalimumab or alemtuzumab or antipyrine or apremilast or ascriptin or aspirin or azathioprine or azelaic acid or balsalazide or belimumab or brimonidine or celecoxib or clonixin lysine or clonixin or curcumin or dexibuprofen or dexketoprofen or diclofenac or dipyrone or etodolac or etoricoxib or fenoprofen or flurbiprofen or ibuprofen or icosapentaenoic acid or indometacin or indomethacin or ketoprofen or ketorolac or leflunomide or lornoxicam or loxoprofen or lumiracoxib or meclofenamic acid or mefenamic acid or meloxicam or mesalamine or mesalazine or methotrexate or nabumetone or naproxen or natalizumab or nimesulide or parecoxib or phenylbutazone or piroxicam or pirprofen or rasagiline or rituximab or rofecoxib or ruxolitinib or salazosulfapyridine or salicylates or salicylic acid or satralizumab or sulfasalazine or sulindac or tenoxicam or teriflunomide or tofacitinib or tolmetin or valdecoxib).tw,kf. | 307676 |
| 26 | exp Angiotensin Receptor Antagonists/ | 26926 |
| 27 | ("angiotensin ii receptor antagonist*" or "angiotensin ii receptor blocker*" or "angiotensin ii receptor blocking agent*" or "angiotensin receptor antagonist" or "angiotensin receptor blocker*" or "angiotensin receptor blocking agent*").tw,kf. | 14264 |
| 28 | irbesartan/ or olmesartan medoxomil/ or telmisartan/ or valsartan/ | 6658 |
| 29 | ("angiotensin 1 receptor antagonist" or "angiotensin 2 receptor antagonist" or azilsartan or candesartan or eprosartan or fimasartan or irbesartan or losartan or olmesartan or sartan derivative or tasosartan or telmisartan or trv027 or valsartan).tw,kf. | 20660 |
| 30 | exp Antidepressive Agents/ | 157396 |
| 31 | ("anti depressant agent*" or "antidepressant*" or "antidepression drug*" or "antidepressive agent*" or "antidepressive drug*" or "neurothymoleptic agent*" or "psychoenergizer" or "thymoanaleptic" or "thymoanaleptics" or thymoleptic or thymoleptics or "thymolytic agent").tw,kf. | 73764 |
| 32 | Aripiprazole/ or Duloxetine Hydrochloride/ or Lithium Carbonate/ or Lithium Compounds/ or Mirtazapine/ or Moclobemide/ or Phenelzine/ or Pizotyline/ or Quetiapine Fumarate/ or Sertraline/ or Tranylcypromine/ | 21662 |
| 33 | Bupropion/ or Citalopram/ or Fluoxetine/ or Mianserin/ or Paroxetine/ or Sulpiride/ or Trazodone/ or Tryptophan/ or Venlafaxine Hydrochloride/ | 61997 |
| 34 | Amitriptyline/ or Clomipramine/ or Desipramine/ or Dothiepin/ or Doxepin/ or Imipramine/ or Nortriptyline/ or Opipramol/ | 24431 |
| 35 | (agomelatine or amitriptyline or aprepitant or aripiprazole or asenapine or "botulinum toxin a" or bupropion or citalopram or clomipramine or desipramine or dothiepin or doxepin or duloxetine hydrochloride or esketamine or fluoxetine or gepirone or imipramine or indalpine or ipsapirone or lithium acetate or lithium carbonate or lithium chloride or lithium compounds or lithium salt or mianserin or mifepristone or minaprine or mirtazapine or moclobemide or monoamine oxidase inhibitor* or noradrenalin uptake inhibitor* or nortriptyline or opipramol or paroxetine or phenelzine or pizotyline or quetiapine fumarate or serotonin uptake inhibitor* or sertraline or sulpiride or tetracyclic antidepressant* or tranylcypromine or trazodone or tricyclic antidepressant* or tryptophan or venlafaxine hydrochloride).tw,kf. | 143344 |
| 36 | Botulinum Toxins, Type A/ | 10662 |
| 37 | ("abobotulinum toxin a" or "abobotulinumtoxin a" or "abobotulinumtoxina" or "agn 151607" or "agn151607" or "alluzience" or "ant 1207" or "ant 1401" or "ant 1403" or "ant1207" or "ant1401" or "ant1403" or "azzalure" or "bocouture" or "boe−tox" or "bont a" or "bont serotype a" or "botox" or "botulin a" or "botulin toxin a" or "botulinium a toxin" or "botulinum a exotoxin" or "botulinum a toxin" or "botulinum neurotoxin a" or "botulinum toxin a" or "btxa" or "clostridium botulinum a toxin" or "clostridium botulinum endotoxin" or "clostridium botulinum neurotoxin a" or "clostridium botulinum toxin type a" or "clostridium botulinum type a neurotoxin" or "cnt 52120" or "cnt52120" or "daxibotulinum toxin a" or "daxibotulinumtoxin a" or "daxibotulinumtoxina" or "dwp 450" or "dwp450" or "dyslor" or "dysport" or "evabotulinum toxin a" or "evabotulinumtoxin a" or "evabotulinumtoxina" or "evosyal" or "gemibotulinum toxin a" or "gemibotulinumtoxin a" or "gemibotulinumtoxina" or "gsk 1358820" or "gsk1358820" or "incobotulinum toxin a" or "incobotulinumtoxin a" or "incobotulinumtoxina" or "ipn 59011" or "ipn59011" or "jeuveau" or "letibotulinum toxin a" or "letibotulinumtoxin a" or "letibotulinumtoxina" or "meditoxin" or "mt 10109" or "mt10109" or "nabota" or "neuronox" or "nivobotulinum toxin a" or "nivobotulinumtoxin a" or "nivobotulinumtoxina" or "nt 201" or "nt201" or "nuceiva" or "oculinum" or "onabotulinum toxin a" or "onabotulinumtoxin a" or "onabotulinumtoxina" or "onaclostox" or "pm 12759" or "pm12759" or "prabotulinum toxin a" or "prabotulinumtoxin a" or "prabotulinumtoxina" or "prosigne" or "purtox" or "qm 1114" or "qm1114" or "relabotulinum toxin a" or "relabotulinumtoxin a" or "relabotulinumtoxina" or "reloxin" or "rtt 150" or "rtt150" or "vistabel" or "vistabex" or "xeomeen" or "xeomin").tw,kf. | 9189 |
| 38 | (caomet or "coenzyme 910" or "coenzyme q 10" or "coenzyme Q10" or decorenone or mitocor or neuquinone or "quinone q 10" or ubidecarenone or ubimaior or "ubiquinone (10)" or "ubiquinone 10" or "ubiquinone 50" or ubiten).tw,kf. | 5376 |
| 39 | Magnesium/ | 69283 |
| 40 | (magnesium or romag).tw,kf. | 66468 |
| 41 | Melatonin/ | 22186 |
| 42 | ("apl 510" or "apl510" or ceyesto or circadin or "jan 13004" or "jan13004" or "ki 1001" or "ki1001" or melatonin or melatonina or melovine or orlogin or slenyto or "sp 13004" or "sp13004" or waferest).tw,kf. | 28086 |
| 43 | or/4-5,8-42 | 1183314 |
| 44 | 3 and 43 [migraines AND prophylaxis] | 9301 |
| 45 | randomized controlled trial.pt. | 574929 |
| 46 | controlled clinical trial.pt. | 94985 |
| 47 | randomized.ab. | 571967 |
| 48 | placebo.ab. | 230792 |
| 49 | drug therapy.fs. | 2519979 |
| 50 | randomly.ab. | 389035 |
| 51 | trial.ab. | 612187 |
| 52 | groups.ab. | 2393254 |
| 53 | 45 or 46 or 47 or 48 or 49 or 50 or 51 or 52 | 5433260 |
| 54 | animals/ not humans.sh. | 5002581 |
| 55 | 53 not 54 | 4733125 |
| 56 | 44 and 55 [migraines AND prophylaxis AND RCTs] | 5941 |
| 57 | remove duplicates from 56 | 5913 |

**EBM Reviews - Cochrane Central Register of Controlled Trials**July 2022
Search Strategy:

| **#** | **Searches** | **Results** |
| --- | --- | --- |
| 1 | exp Migraine Disorders/ | 3020 |
| 2 | (migraine or migraines or sick headache or sick headaches or hemicrania or migrainous headache or status hemicranicus).tw,kw. | 9024 |
| 3 | 1 or 2 | 9137 |
| 4 | ("cgrp monoclonal antibodies" or "calcitonin gene-related peptide antibodies (cgrp) monoclonal antibodies (mabs)" or "cgrp mabs").tw,kw. | 13 |
| 5 | calcitonin gene related peptide.tw,kw. | 1062 |
| 6 | Calcitonin Gene-Related Peptide Receptor Antagonists/ | 81 |
| 7 | exp Antibodies, Monoclonal/ | 15809 |
| 8 | 6 and 7 | 42 |
| 9 | adrenergic beta-antagonists/ | 4417 |
| 10 | ("adrenergic beta antagonist*" or "adrenergic beta blocker*" or "adrenergic beta receptor blockader*" or "beta adrenergic antagonist*" or "beta adrenergic blocker*" or "beta adrenergic blocking agent*" or "beta adrenergic blocking drug" or "beta adrenergic receptor antagonist*" or "beta adrenergic receptor blockader*" or "beta adrenergic receptor blocker*" or "beta adrenoceptor antagonist*" or "beta adrenoceptor blocker*" or "beta adrenoceptor blocking agent*" or "beta adrenoceptor blocking drug*" or "beta adrenolytic" or "beta antagonist*" or "beta antiadrenergic agent*" or "beta blocker" or "beta blocking adrenergic agent*" or "beta blocking agent*" or "beta blocking drug*" or "beta receptor adrenergic blocking agent*" or "beta receptor blocker*" or "beta receptor blocking agent*" or "beta sympathicolytic agent*" or "beta sympathicolytics" or "beta sympatholytic agent*" or "betasympatholytic agent*").tw,kw. | 6202 |
| 11 | alprenolol/ or "brimonidine tartrate, timolol maleate drug combination"/ or bunolol/ or bupranolol/ or carteolol/ or carvedilol/ or dihydroalprenolol/ or iodocyanopindolol/ or labetalol/ or levobunolol/ or metipranolol/ or nadolol/ or oxprenolol/ or penbutolol/ or pindolol/ or propranolol/ or sotalol/ or timolol/ | 6240 |
| 12 | (alprenolol or bucindolol or bunolol or bupranolol or carazolol or carpindolol or carteolol or carvedilol or cloranolol or dexpropranolol or dichlorisoprenaline or dihydroalprenolol or dilevalol or iodocyanopindolol or isamoltane or labetalol or levobunolol or metipranolol or nadolol or nifenalol or oxprenolol or penbutolol or pindolol or propranolol or sotalol or spirendolol or tertatolol or tilisolol or timolol).tw,kw. | 10888 |
| 13 | adrenergic beta-1 receptor antagonists/ | 215 |
| 14 | acebutolol/ or atenolol/ or betaxolol/ or bisoprolol/ or celiprolol/ or metoprolol/ or practolol/ | 4201 |
| 15 | (acebutolol or atenolol or betaxolol or bevantolol or bisoprolol or celiprolol or cetamolol or cyanoiodopindolol or cyanopindolol or esmolol or landiolol or metoprolol or nebivolol or practolol or propranolol or ritodrine or sotalol or talinolol or vortioxetine).tw,kw. | 13586 |
| 16 | Calcium Channel Blockers/ | 2876 |
| 17 | ("calcium blocker" or "calcium blocking agent*" or "calcium channel antagonist*" or "calcium channel blocker*" or "calcium channel blocking drug*" or "calcium entry blocker" or "calcium entry blocking agent*" or "calcium inhibitor" or "exogenous calcium antagonist*" or "exogenous calcium blockader*").tw,kw. | 3325 |
| 18 | Anticonvulsants/ | 2440 |
| 19 | ("anti convulsant agent*" or "anti convulsive agent*" or "anti convulsive drug*" or "anti epileptic*" or "anticonvulsant*" or "anticonvulsive agent*" or "anticonvulsive drug*" or "anticonvulsivum" or "antiepileptic" or "antiepileptics" or "antiepileptiform drug*").tw,kw. | 4495 |
| 20 | acetazolamide/ or bromides/ or cannabidiol/ or carbamazepine/ or chlormethiazole/ or clobazam/ or clonazepam/ or clorazepate dipotassium/ or diazepam/ or dimethadione/ or estazolam/ or ethosuximide/ or felbamate/ or flunarizine/ or gabapentin/ or lacosamide/ or lamotrigine/ or levetiracetam/ or lorazepam/ or magnesium sulfate/ or medazepam/ or mephenytoin/ or mephobarbital/ or meprobamate/ or nitrazepam/ or oxcarbazepine/ or paraldehyde/ or phenobarbital/ or phenytoin/ or pregabalin/ or primidone/ or riluzole/ or thiopental/ or tiagabine/ or tiletamine/ or topiramate/ or trimethadione/ or valproic acid/ or vigabatrin/ or zonisamide/ | 11619 |
| 21 | (acetazolamide or albutoin or alprazolam or ascorbate magnesium or belnacasan or brexanolone or brivaracetam or bromides or cannabidiol or cannabidivarin or carabersat or carbamazepine or carisbamate or cenobamate or chlormethiazole or clobazam or clomethiazole or clonazepam or clorazepate dipotassium or darigabat or dextromethorphan or dezinamide or diazepam or diclofenamide or dimethadione or elpetrigine or estazolam or eterobarb or ethosuximide or ethotoin or etiracetam or felbamate or fenfluramine or flunarizine or gabapentin or ganaxolone or guaifenesin or "ica 105665" or imepitoin or imidazenil or lacosamide or lamotrigine or lanicemine or levetiracetam or licarbazepine or lorazepam or loreclezole or losigamone or magnesium sulfate or medazepam or mephenytoin or mephobarbital or meprobamate or mesuximide or metharbital or midazolam or nitrazepam or oxcarbazepine or padsevonil or paraldehyde or paramethadione or pentoxyverine or perampanel or phenobarbital or phenytoin or pregabalin or primidone or progabide or remacemide or retigabine or riluzole or ropizine or rufinamide or safinamide or seletracetam or sodium bromide or stiripentol or sultiame or talampanel or thiopental or tiagabine or tiletamine or topiramate or trimethadione or valproic acid or valpromide or valrocemide or vigabatrin or vixotrigine or zaleplon or zonisamide).tw,kw. | 34246 |
| 22 | exp Anti-Inflammatory Agents, Non-Steroidal/ | 21883 |
| 23 | ("anti inflammatory analgesic*" or "aspirin like agent*" or "non steroid antiinflammatory agent*" or "non steroid antiinflammatory drug*" or "non steroidal anti inflammatory agent*" or "non steroidal anti inflammatory drug*" or "non steroidal antiinflammatory agent*" or "non steroidal antiinflammatory drug*" or "nonsteroid antiinflammatory agent*" or "nonsteroid antiinflammatory drug" or "nonsteroid antirheumatic agent" or "nonsteroidal anti inflammatory agent*" or "nonsteroidal anti inflammatory drug*" or "nonsteroidal antiinflammatory agent*" or "nonsteroidal antiinflammatory drug*" or "nsaid" or "nsaids").tw,kw. | 11038 |
| 24 | Acetaminophen/ or Antipyrine/ or Aspirin/ or Celecoxib/ or Clonixin/ or Curcumin/ or Diclofenac/ or dipyrone/ or Ibuprofen/ or Indomethacin/ or Ketoprofen/ or Ketorolac/ or Ketorolac Tromethamine/ or Mesalamine/ or Naproxen/ or salicylates/ or Sulfasalazine/ or Tolmetin/ | 19158 |
| 25 | (acalabrutinib or aceclofenac or acemetacin or acetaminophen or acetaminosalol or acetylsalicylic acid or actarit or adalimumab or alemtuzumab or antipyrine or apremilast or ascriptin or aspirin or azathioprine or azelaic acid or balsalazide or belimumab or brimonidine or celecoxib or clonixin lysine or clonixin or curcumin or dexibuprofen or dexketoprofen or diclofenac or dipyrone or etodolac or etoricoxib or fenoprofen or flurbiprofen or ibuprofen or icosapentaenoic acid or indometacin or indomethacin or ketoprofen or ketorolac or leflunomide or lornoxicam or loxoprofen or lumiracoxib or meclofenamic acid or mefenamic acid or meloxicam or mesalamine or mesalazine or methotrexate or nabumetone or naproxen or natalizumab or nimesulide or parecoxib or phenylbutazone or piroxicam or pirprofen or rasagiline or rituximab or rofecoxib or ruxolitinib or salazosulfapyridine or salicylates or salicylic acid or satralizumab or sulfasalazine or sulindac or tenoxicam or teriflunomide or tofacitinib or tolmetin or valdecoxib).tw,kw. | 70589 |
| 26 | exp angiotensin receptor antagonists/ | 4178 |
| 27 | ("angiotensin ii receptor antagonist*" or "angiotensin ii receptor blocker*" or "angiotensin ii receptor blocking agent*" or "angiotensin receptor antagonist" or "angiotensin receptor blocker*" or "angiotensin receptor blocking agent*").tw,kw. | 3582 |
| 28 | irbesartan/ or olmesartan medoxomil/ or telmisartan/ or valsartan/ | 2080 |
| 29 | ("angiotensin 1 receptor antagonist" or "angiotensin 2 receptor antagonist" or azilsartan or candesartan or eprosartan or fimasartan or irbesartan or losartan or olmesartan or sartan derivative or tasosartan or telmisartan or trv027 or valsartan).tw,kw. | 8620 |
| 30 | exp antidepressive agents/ | 15881 |
| 31 | ("anti depressant agent*" or "antidepressant*" or "antidepression drug*" or "antidepressive agent*" or "antidepressive drug*" or "neurothymoleptic agent*" or "psychoenergizer" or "thymoanaleptic" or "thymoanaleptics" or thymoleptic or thymoleptics or "thymolytic agent").tw,kw. | 13906 |
| 32 | Aripiprazole/ or Duloxetine Hydrochloride/ or Lithium Carbonate/ or Lithium Compounds/ or Mirtazapine/ or Moclobemide/ or Phenelzine/ or Pizotyline/ or Quetiapine Fumarate/ or Sertraline/ or Tranylcypromine/ | 4101 |
| 33 | Bupropion/ or Citalopram/ or Fluoxetine/ or Mianserin/ or Paroxetine/ or Sulpiride/ or Trazodone/ or Tryptophan/ or Venlafaxine Hydrochloride/ | 6608 |
| 34 | Amitriptyline/ or Clomipramine/ or Desipramine/ or Dothiepin/ or Doxepin/ or Imipramine/ or Nortriptyline/ or Opipramol/ | 3410 |
| 35 | (agomelatine or amitriptyline or aprepitant or aripiprazole or asenapine or "botulinum toxin a" or bupropion or citalopram or clomipramine or desipramine or dothiepin or doxepin or duloxetine hydrochloride or esketamine or fluoxetine or gepirone or imipramine or indalpine or ipsapirone or lithium acetate or lithium carbonate or lithium chloride or lithium compounds or lithium salt or mianserin or mifepristone or minaprine or mirtazapine or moclobemide or monoamine oxidase inhibitor* or noradrenalin uptake inhibitor* or nortriptyline or opipramol or paroxetine or phenelzine or pizotyline or quetiapine fumarate or serotonin uptake inhibitor* or sertraline or sulpiride or tetracyclic antidepressant* or tranylcypromine or trazodone or tricyclic antidepressant* or tryptophan or venlafaxine hydrochloride).tw,kw. | 25811 |
| 36 | Botulinum Toxins, Type A/ | 1835 |
| 37 | ("abobotulinum toxin a" or "abobotulinumtoxin a" or "abobotulinumtoxina" or "agn 151607" or "agn151607" or "alluzience" or "ant 1207" or "ant 1401" or "ant 1403" or "ant1207" or "ant1401" or "ant1403" or "azzalure" or "bocouture" or "boe−tox" or "bont a" or "bont serotype a" or "botox" or "botulin a" or "botulin toxin a" or "botulinium a toxin" or "botulinum a exotoxin" or "botulinum a toxin" or "botulinum neurotoxin a" or "botulinum toxin a" or "btxa" or "clostridium botulinum a toxin" or "clostridium botulinum endotoxin" or "clostridium botulinum neurotoxin a" or "clostridium botulinum toxin type a" or "clostridium botulinum type a neurotoxin" or "cnt 52120" or "cnt52120" or "daxibotulinum toxin a" or "daxibotulinumtoxin a" or "daxibotulinumtoxina" or "dwp 450" or "dwp450" or "dyslor" or "dysport" or "evabotulinum toxin a" or "evabotulinumtoxin a" or "evabotulinumtoxina" or "evosyal" or "gemibotulinum toxin a" or "gemibotulinumtoxin a" or "gemibotulinumtoxina" or "gsk 1358820" or "gsk1358820" or "incobotulinum toxin a" or "incobotulinumtoxin a" or "incobotulinumtoxina" or "ipn 59011" or "ipn59011" or "jeuveau" or "letibotulinum toxin a" or "letibotulinumtoxin a" or "letibotulinumtoxina" or "meditoxin" or "mt 10109" or "mt10109" or "nabota" or "neuronox" or "nivobotulinum toxin a" or "nivobotulinumtoxin a" or "nivobotulinumtoxina" or "nt 201" or "nt201" or "nuceiva" or "oculinum" or "onabotulinum toxin a" or "onabotulinumtoxin a" or "onabotulinumtoxina" or "onaclostox" or "pm 12759" or "pm12759" or "prabotulinum toxin a" or "prabotulinumtoxin a" or "prabotulinumtoxina" or "prosigne" or "purtox" or "qm 1114" or "qm1114" or "relabotulinum toxin a" or "relabotulinumtoxin a" or "relabotulinumtoxina" or "reloxin" or "rtt 150" or "rtt150" or "vistabel" or "vistabex" or "xeomeen" or "xeomin").tw,kw. | 3672 |
| 38 | (caomet or "coenzyme 910" or "coenzyme q 10" or "coenzyme Q10" or decorenone or mitocor or neuquinone or "quinone q 10" or ubidecarenone or ubimaior or "ubiquinone (10)" or "ubiquinone 10" or "ubiquinone 50" or ubiten).tw,kw. | 1080 |
| 39 | Magnesium/ | 1229 |
| 40 | (magnesium or romag).tw,kw. | 7739 |
| 41 | Melatonin/ | 1328 |
| 42 | ("apl 510" or "apl510" or ceyesto or circadin or "jan 13004" or "jan13004" or "ki 1001" or "ki1001" or melatonin or melatonina or melovine or orlogin or slenyto or "sp 13004" or "sp13004" or waferest).tw,kw. | 3246 |
| 43 | or/4-5,8-42 | 197886 |
| 44 | 3 and 43 | 3267 |

# Supplement 2 – Risk of bias criteria

| **Bias from the randomization process** | |
| --- | --- |
| Issues to consider:  Random sequence generation  Allocation concealment | |
| **Definitely low risk of bias** | Trials that assign participants to alternative interventions using a randomly generated sequence and maintain allocation concealment.  Examples of methods for developing a randomly generated allocation sequence include a random number generator, random number table, coin tossing, shuffling cards or envelopes, and throwing dice. If a trial is described as 'randomized' without any additional details related to how the allocation sequence was developed, we will assume that the allocation sequence was appropriately developed.  Examples of methods for maintaining allocation concealment include using central allocation via a computer or phone system, pharmacy-controlled allocation, opaque sealed envelopes, and sequentially numbered drug containers.  *Note that an explicit description of random sequence generation is not necessary for a rating of low risk of bias.* |
| **Probably low risk of bias** | Trials in which healthcare providers were blind to the intervention but which provide no information on allocation concealment.  *Note that an explicit description of random sequence generation is not necessary for a rating of probably low risk of bias.* |
| **Probably high risk of bias** | Trials in which healthcare providers were not blind to the intervention and which provide no information on allocation concealment.  Trials in which there are substantial baseline differences between trial arms that suggest a problem with the randomization process but there are no other limitations related to randomization. |
| **Definitely high risk of bias** | Trials in which allocation is by judgment of the clinician, by preference of the participant, by availability of the intervention, based on the results of a laboratory test, or other non-random rules (e.g., birthdate, etc.).  Trials in which investigators enrolling participants could possibly foresee the arm to which each subsequent patient would be randomized, such as allocation using an open allocation schedule (e.g. a list of random numbers), assignment envelopes used without appropriate safeguards (e.g. use of unsealed, non-opaque or not sequentially numbered envelopes), alternation between arms, case record number, or any other explicitly unconcealed procedure, rate as high risk. |
| **Bias due to deviations from the intended intervention** | |
| Issues to consider:  Blinding of healthcare providers/clinicians and participants  Imbalances in cointerventions or behaviors | |
| **Definitely low risk of bias** | Trials in which healthcare providers are blind to the intervention administered and in which there are no significant differences in administered co-interventions.  Trials that are described as double or triple blind. |
| **Probably low risk of bias** |  |
| **Probably high risk of bias** | Trials in which healthcare providers are not blind to the intervention administered.  Trials in which healthcare providers are blind to the intervention administered but there are significant differences in administered co-interventions that suggests that blinding may have been compromised.  Trials in which healthcare providers are described as being blind to the intervention but allocation concealment was inadequate. |
| **Definitely high risk of bias** | Trials in which healthcare providers are not blind to the intervention and in which there are significant differences in administered co-interventions. |
| **Bias due to missing data** | |
| Issues to consider:  Missing outcome measures  Loss to follow-up | |
| **Definitely low risk of bias** | Trials in which missing outcome data (including outcome data that has been imputed) < 10%. |
| **Probably low risk of bias** | Trials in which missing outcome data (including outcome data that has been imputed) is between 10% to 15% and missing outcome data is unlikely to be related to the true outcome and there is no imbalance in numbers of or reasons for missing data across intervention groups. |
| **Probably high risk of bias** | Trials in which missing outcome data (including outcome data that has been imputed) is between 10% to 15% and missing outcome data is likely to be related to the true outcome or there are imbalances in numbers of or reasons for missing data across intervention groups. |
| **Definitely high risk of bias** | Trials in which missing outcome data (including outcome data that has been imputed) > 15%. |
| **Bias due to measurement of the outcome** | |
| Issues to consider:  Blinding of outcome adjudicators  Objectivity of outcome  *Note that the judgments may differ across outcomes.* | |
| **Definitely low risk of bias** | Trials in which patients are blind to the intervention and in which outcomes are patient-reported.  Trials in which outcomes are measured by a third-party (investigator or clinician) and in which the third-party is blind to the intervention.  Trials in which the outcomes are objective (e.g., mortality, hospitalization).  Trials that are described as double or triple blind. |
| **Probably low risk of bias** |  |
| **Probably high risk of bias** |  |
| **Definitely high risk of bias** | Trials in which patients are not blind and in which outcomes are patient-reported (e.g., ACQ).  Trials in which outcome adjudicators are not blind and the outcomes are not objective (e.g., adverse events leading to discontinuation). |
| **Bias in selection of the reported results** | |
| Issues to consider:  Selective reporting of timepoints  Selective reporting of outcome measures  *Note that we are only interested in selective reporting for the outcomes for which we are extracting data.*  *Note that the judgments may differ across outcomes.* | |
| **Definitely low risk of bias** | Results for outcomes that were analyzed and reported according to a pre-specified statistical analysis plan or protocol (including the timepoint for the measurement of the outcome). |
| **Probably low risk of bias** | Results for outcomes that were analyzed and reported but that were not prespecified in a statistical analysis plan or protocol but the timepoint at which results are reported is consistent with the timepoint for other outcomes in the trial report or there is little reason to believe the outcome was selectively reported.  Please note that outcomes that were not prespecified in a protocol or statistical analysis plan and that are reported in the trial preprint or publication should be rated at probably low risk of bias unless there are other important reasons to suspect that results for those outcomes were selectively reported (e.g., results are presented at timepoints that don’t match the timepoints reported for other outcomes). |
| **Probably high risk of bias** | Results for outcomes that were analyzed and reported but that were not prespecified in a statistical analysis plan or protocol but the timepoint at which results are reported is not consistent with the timepoint for other outcomes in the trial report or there are other reasons to believe that the outcome is selectively reported. |
| **Definitely high risk of bias** | Results for outcomes that were analyzed and reported for which there are inconsistencies with the statistical analysis plan or protocol. These inconsistencies may include outcome measures of interest or the timepoints for the measurement of outcomes. |

# Supplement 3 – Sensitivity analysis restricted to recommended therapeutic doses of drugs

We performed a sensitivity analysis in which we restricted trial arms to only those that tested recommended therapeutic doses of drugs.

| **Drugs** | **Minimum dose** |
| --- | --- |
| Amitriptyline | 10 mg/day |
| Beta-blocker | Bisoprolol: 5 mg/day  Propranolol: 40 mg/day |
| Calcium channel blocker | Flunarizine: 5 mg/day  Cinnarizine: 15 mg/day |
| Carisbamate | 100 mg/day |
| Eptinezumab | 100 mg/month |
| Erenumab | 70 mg/month |
| Fremanezumab | 225 mg/month or 675 mg/3 months |
| Gabapentin | 100 mg/day |
| Galcanezumab | 120 mg/month |
| Gepant | Atogepant: 10 mg/day  Rimegepant: 75 mg/day  Telcagepant: 280 mg/day |
| Oxcarbezapine | 300 mg/day |
| Topiramate | 50 mg/day |
| Valproate | 500 mg/day |

Ducros A, de Gaalon S, Roos C, Donnet A, Giraud P, Guégan-Massardier E, Lantéri-Minet M, Lucas C, Mawet J, Moisset X, Valade D, Demarquay G. Revised guidelines of the French headache society for the diagnosis and management of migraine in adults. Part 2: Pharmacological treatment. Rev Neurol (Paris). 2021 Sep;177(7):734-752. doi: 10.1016/j.neurol.2021.07.006. Epub 2021 Jul 30. PMID: 34340810.

# Supplement 4 – Table of trial characteristics

| Study | Trial Name | Registration | Funding | Country | Age | % Male | Duration of Migraine (years) | % Aura | % With Previous Prophylaxis | Migraine/ Headache Days Per Month | Interventions |
| --- | --- | --- | --- | --- | --- | --- | --- | --- | --- | --- | --- |
| Ailani, 2021 (1) | ADVANCE | NCT03777059 | Allergan | United States | 41.6 | 11.2 | NR | NR | 70.3 | 7.4 | Atogepant (10mg): 10 mg/day for 12 weeks, oral Atogepant (30mg): 30 mg/day for 12 weeks, oral Atogepant (60mg): 60 mg/day for 12 weeks, oral Placebo |
| Ashina, 2020 (2) | PROMISE-1 | NCT02559895 | H. Lundbeck A/S | United States | 39.8 | 15.7 | 17.4 | NR | NR | 8.6 | Eptinezumab (30mg): 30 mg, q12w, IV Eptinezumab (100mg): 100 mg, q12w, IV Eptinezumab (300mg): 300 mg, q12w, IV Placebo |
| Ashina, 2022 (3) | DELIVER | NCT04418765 | H. Lundbeck A/S | Belgium, Bulgaria, Czechia, Denmark, Finland, France, Georgia, Germany, Hungary, Italy, Poland, Russia, Slovakia, Spain, Sweden, United Kingdom, United States | 43.8 | 10.1 | 14.6 | 29.6 | 100.0 | 13.8 | Eptinezumab (100mg): 100 mg, day 0 and week 12, IV Eptinezumab (300mg): 300 mg, day 0 and week 12, IV  Placebo |
| Bigal, 2015 (4) | NR | NCT02025556 | Teva Pharmaceuticals | United States | 41.2 | 12.1 | 19.0 | NR | 29.0 | 11.4 | Fremanezumab (225mg):225 mg/day q28d for 12 weeks, SC Fremanezumab (675mg): 675mg q28d for 12 weeks, SC Placebo |
| Bigal, 2016 (5) | NR | NCT02021773 | Teva Pharmaceuticals | United States | 40.7 | 14.1 | 18.3 | NR | 40.3 | 16.8 | Fremanezumab (675/225mg): 675 mg loading dose, 225 mg/day q28d for 12 weeks, SC Fremanezumab (900mg): 900 mg, q28d for 12 weeks, SC Placebo |
| Bostani, 2013 (6) | NR | IRCT201102055729N2 | Kermanshah University of Medical Sciences | Iran | 32.1 | 31.7 | 6.4 | 17.3 | NR | 6.8 | Valproate: 200 mg/day for 12 weeks, oral Cinnarizine: 25 mg/day for 12 weeks, oral |
| Brandes, 2004 (7) | MIGR-002 | NCT00231595 | Johnson and Johnson Pharmaceutical Research and Development | Canada, United States | 38.9 | 13.2 | NR | NR | NR | 6.5 | Topiramate (50mg): 50 mg titrated over 8 weeks, maintained for 18 weeks, oral Topiramate (100mg): 100 mg titrated over 8 weeks, maintained for 18 weeks, oral Topiramate (200mg): 200 mg titrated over 8 weeks, maintained for 18 weeks, oral Placebo |
| Cady, 2009 (8) | NR | NCT00109083 | Johnson and Johnson Pharmaceutical Research and Development | Germany, Spain, United States | 41.3 | 14.6 | 19.2 | 46.8 | NR | 8.1 | Carisbamate (100mg): 100 mg/day titrated over 2 weeks, maintained for 12 weeks, oral  Carisbamate (300mg): 300 mg/day titrated over 2 weeks, maintained for 12 weeks, oral  Carisbamate (600mg): 600 mg/day titrated over 2 weeks, maintained for 12 weeks, oral Placebo |
| Camporeale, 2018 (9) | NR | NCT02614287 | Eli Lilly and Company | Belgium, Canada, France, Hungary, United States | 42.0 | 17.4 | 20.8 | NR | 62.6 | 10.6 | Galcanezumab (120mg): 120 mg/month for 52 weeks, SC  Galcanezumab (240 mg): 240 mg/month for 52 weeks, SC |
| Chowdhury, 2021 (10) | TOP-PRO | CTRI/2019/05/018997 | None | India | 33.0 | 4.6 | 6.0 | 4.6 | NR | 17.4 | Topiramate: 100 mg/day titrated over 4 weeks, maintained for 20 weeks, oral Propranolol: 80 mg/day titrated over 4 weeks, maintained for 20 weeks, oral |
| Couch, 1979 (11) | NR | NR | Merck Laboratories | United States | NR | 16.0 | NR | NR | NR | NR | Amitriptyline: 100 mg/day or MTD, titrated over 4 weeks, maintained for 4 weeks, oral Placebo |
| Couch, 2011 (12) | NR | NR | Merck, Sharp, and Dohme Research Laboratories | United States | 34.9 | 19.0 | NR | NR | NR | NR | Amitriptyline: 100 mg/day or MTD, titrated over 4 weeks, maintained for 12 weeks, oral Placebo |
| Croop, 2021 (13) | NR | NCT03732638 | Biohaven Pharmaceuticals | United States | 41.2 | 17.0 | NR | 40.0 | NR | 10.1 | Rimegepant: 75 mg every other day for 12 weeks, oral Placebo |
| Detke, 2018 (14) | REGAIN | NCT02614261 | Eli Lilly and Company | Argentina, Canada, Czech Republic, Germany, Israel, Italy, Mexico, Netherlands, Spain, Taiwan, United Kingdom, United States | 41.0 | 15.0 | 21.1 | 54.3 | 77.8 | 19.5 | Galcanezumab (120mg): 120 mg, q4w for 12 weeks, SC Galcanezumab (240mg): 240 mg, q4w for 12 weeks, SC Placebo |
| Diener, 1996 (15) | NR | NR | NR | NR | 39.0 | 22.0 | 19.0 | 26.2 | NR | 4.0 | Propranolol: 120 mg/day for 12 weeks, oral Placebo |
| Diener, 2002 (16) | NR | NR | Janssen | Belgium, Denmark, France, Germany, Italy, Portugal, Spain, Switzerland | 37.0* | 18.6 | 10.0* | 28.2 | NR | NR | Flunarizine (5mg): 5 mg/day for 16 weeks, oral Flunarizine (10mg): 10 mg/day for 16 weeks (placebo administered on 2/7 days), oral Propranolol: 160 mg/day titrated over 1 weeks and maintained for 8 weeks, oral |
| Diener, 2004 (17) | MIGR-003 | NCT00236561 | Johnson & Johnson Pharmaceutical Research and Development | Australia, Denmark, Finland, France, Germany, Italy, Korea, Netherlands, South African, Spain, Sweden, Taiwan, United Kingdom | 40.9 | 20.3 | NR | NR | NR | 6.1 | Topiramate (100mg): 100 mg/day titrated over 8 weeks and maintained for 18 weeks, oral Topiramate (200mg): 200 mg/day titrated over 8 weeks and maintained for 18 weeks, oral Propranolol (160mg): 160 mg/day titrated over 8 weeks and maintained for 18 weeks, oral Placebo |
| Diener, 2007 (18) | PROMPT | NR | Janssen-Cilag EMEA | Austria, Belgium, Bulgaria, Czech Republic, Denmark, France, Germany, Greece, Hungary, Ireland, Italy, Norway, Poland, Portugal, Russia, Saudi Arabia, Slovenia, Spain, Switzerland, Turkey, UK | 39.8 | 13.0 | NR | NR | NR | 8.9 | Topiramate:100mg/day or MTD, titrated over 4 weeks and maintained for 12 weeks, oral Placebo |
| Dodick, 2007(19) | NR | NR | Ortho-McNeil Janssen | United States | 38.2 | 14.7 | 9.2 | NR | NR | 17.1 | Topiramate: 100 mg/day or MTD, titrated over 4 weeks maintained for 12 weeks, oral Placebo |
| Dodick, 2009 (20) | NR | NR | Ortho-McNeil Janssen | United States | 38.8 | 15.1 | NR | NR | NR | 7.3 | Topiramate: 100 mg/day or MTD, titrated over 4 weeks and maintained for 22 weeks, oral Amitriptyline: 100 mg/day or MTD titrated over 4 weeks and maintained for 22 weeks, oral |
| Dodick, 2014 (21) | NR | NCT01625988 | Arteaus Therapeutics | United States | 41.4 | 15.2 | NR | 41.5 | NR | 6.9 | Galcanezumab: 150 mg, q2w for 12 weeks, SC Placebo |
| Dodick, 2014 (22) | NR | NCT01772524 | Alder Biopharmaceuticals | United States | 38.8 | 18.4 | NR | NR | NR | 8.6 | Eptinezumab: 1000 mg once for 12 weeks, IV Placebo |
| Dodick, 2018 (23) | NR | NCT02629861 | Teva Pharmaceuticals | Canada, Czech Republic, Finland, Israel, Japan, Poland, Russia, Spain, United States | 41.8 | 15.2 | 20.2 | NR | 20.8 | 9.1 | Fremanezumab (225mg): 225 mg, q4w for 12 weeks, SC Fremanezumab (675mg): 675 mg once for 12 weeks, SC Placebo |
| Dodick, 2018 (24) | ARISE | NCT02483585 | Amgen | Denmark, France, Greece, Portugal, Russia, Spain, Switzerland, United States | 42.0 | 14.7 | 21.0 | 50.3 | 46.1 | 8.3 | Erenumab: 70 mg, q4w for 12 weeks, SC Placebo |
| Dodick, 2019 (25) | NR | NCT02275117 | Alder Biopharmaceuticals | Australia, Georgia, New Zealand, United States | 36.6 | 13.2 | 17.9 | NR | 45.6 | 16.5 | Eptinezumab (10mg): 10 mg, once for 12 weeks, IV Eptinezumab (30mg): 30 mg, once for 12 weeks, IV Epitinezumab (100mg): 100 mg, once for 12 weeks, IV Eptinezumab (300mg): 300 mg, once for 12 weeks, IV Placebo |
| Ferrari, 2019 (26) | FOCUS | NCT03308968 | Teva Pharmaceuticals | Belgium, Czechia, Denmark, Finland, France, Germany, Italy, Netherlands, Poland, Spain, Sweden, Switzerland, United Kingdom, United States | 46.2 | 16.5 | 24.9 | NR | 100.0 | 14.2 | Fremanezumab (monthly): 225 mg q4w, SC Fremanezumab (quarterly): 675 mg once, SC Placebo |
| Freitag, 2002 (27) | NR | NR | Abbott Laboratories | United States | 40.5 | 21.1 | 20.2 | 36.3 | 20.6 | 4.3 | Valproate: 500-1000 mg/day, for 12 weeks, oral Placebo |
| Ghasami, 2009 (28) | NR | NR | Arak University of Medical Sciences | Iran | NR | 31.8 | NR | NR | NR | NR | Valproate (15 mg/kg): 1-3 pills/day for 24 weeks, oral Propranolol/antidepressant: 40-80mg/day propranolol with 50mg amitriptyline or 25-50mg/day nortriptyline |
| Ghobadi, 2013 (29) | NR | NR | NR | NR | 47.0 | 16.7 | NR | NR | NR | 15.0 | Propranolol: 40 mg/day for 24 weeks, oral Nimodipine: 30 mg/day for 24 weeks, oral |
| Goadsby, 2017 (30) | STRIVE | NR | Novartis, Amgen | United States | 40.5 | 21.1 | 20.2 | 36.3 | 20.6 | 4.3 | Erenumab (70mg): 70 mg, q4w for 24 weeks, SC Erenumab (140mg): 140 mg, q4w for 24 weeks, SC Placebo |
| Goadsby, 2020 (31) | NR | NCT02638103 | Teva Pharmaceuticals | Canada, Czechia, Finland, Israel, Japan, Poland, Russian Confederation, Spain, United States | 43.5 | 13.0 | 21.6 | NR | 23.9 | 13.4 | Fremanezumab (q12w): 675mg q12w for 52 weeks, SC  Fremanezumab (675/225 q4w chronic): 675 mg loading dose, 225 q4w for 52 weeks, SC Fremanezumab (225mg q4w episodic): 225 mg q4w for 52 weeks, SC |
| Goadsby, 2020 (32) | NR | NCT02848326 | Allergan | United States | 40.1 | 13.5 | 19.4 | 22.3 | 28.1 | 7.7 | Atogepant (10mg): 10 mg/day for 12 weeks, oral Atogepant (30mg): 30 mg/day for 12 weeks, oral Atogepant (60mg): 60 mg/day for 12 weeks, oral  Atogepant (30mg B.I.D): 30mg twice/day for 12 weeks, oral Atogepant (60mg B.I.D): 60 mg twice/day for 12 weeks, oral Placebo |
| Goncalves, 2016(33) | NR | NCT01357031 | Fundação de Amparo a Pesquisa de São Paulo | Brazil | 36.9 | 24.6 | 22.2 | 16.1 | NR | 7.3 | Amitriptyline: 25 mg/day for 12 weeks Placebo |
| Hesami, 2017(34) | NR | IRCT2012070310178N1 | NR | Iran | 36.0 | 10.2 | NR | NR | NR | 10.3 | Pregabalin (100mg): 100mg/day for 12 weeks, oral Valproate (400mg): 400 mg/day for 12 weeks, oral |
| Ho, 2014(35) | NR | NCT00797667 | Merck & Co. | United States | 41.3 | 16.0 | NR | 46.6 | 29.1 | 8.2 | Telcagepant (280 mg): 280mg/day for 12 weeks, oral Telcagepant (560 mg): 560mg/day for 12 weeks, oral Placebo |
| Holroyd, 2010 (36) | NR | NCT00910689 | National Institutes of Health | United States | 38.2 | 21.0 | 15.0 | 22.0 | NR | 8.5 | Propranolol: 180 mg titrated over 12 weeks or MTD, offered nadolol if propranolol was not tolerated, maintained for 12 weeks, oral Placebo |
| Hu, 2022 (37) | PERSIST | NCT03963232 | Eli Lilly and Company | China, India, Russia | 37.0 | 26.2 | 12.6 | NR | 44.6 | 6.3 | Galcanezumab: 240 mg loading dose, 120 mg q4w for 12 weeks, SC Placebo |
| Kalita, 2013 (38) | NR | NR | NR | India | 31.9 | 19.7 | 7.3 | 6.3 | NR | 10.8 | Valproate: 500-1000mg/day or MTD for 22 weeks, oral Amitriptyline: 25-50mg/day or MTD for 22 weeks, oral |
| Klapper, 1997 (39) | NR | NR | Abbott Laboratories | United States | 40.8 | 11.0 | 21.6 | 40.3 | 53.0 | NR | Valproate (500mg): 500 mg/day, titrated over 4 weeks, maintained for 8 weeks, oral Valproate (1000mg): 1000 mg/day, titrated over 4 weeks, maintained for 8 weeks, oral Valproate (1500mg): 1500 mg/day, titrated over 4 weeks, maintained for 8 weeks, oral Placebo |
| Lipton, 2011 (40) | INTREPID | NCT00212810 | Ortho-Mcneil Janssen | NR | 40.3 | 10.9 | NR | NR | NR | 11.7 | Topiramate: 100 mg/day titrated over 6 weeks, maintained for 20 weeks, oral  Placebo |
| Lipton, 2020 (41) | PROMISE-2 | NCT02974153 | H. Lundbeck A/S | Belgium, Czechia, Denmark, Georgia, Germany, Hungary, Italy, Russia, Slovakia, Ukraine, United Kingdom, United States | 40.5 | 11.8 | 18.1 | NR | NR | 16.1 | Eptinezumab (100mg): 100 mg, day 0 and week 12, IV Eptinezumab (300mg): 300 mg, day 0 and week 12, IV Placebo |
| Lucking, 1988 (42) | Trial 1 | NR | NR | NR | 42.1 | 19.5 | NR | NR | NR | NR | Flunarizine: 10 mg/day for 16 weeks, oral Propranolol: 120mg/day titrated over 2 weeks, maintained for 14 weeks, oral |
| Lucking, 1988 (42) | Trial 2 | NR | NR | NR | 42.1 | 19.5 | NR | NR | NR | NR | Flunarizine: 10 mg/day for 16 weeks, oral Propranolol: 120mg/day titrated over 2 weeks, maintained for 14 weeks, oral |
| Luo, 2012 (43) | NR | NR | National Natural Science Foundation of China | China | 43.0 | 28.6 | 4.5 | NR | NR | 4.5 | Flunarizine (5mg): 5 mg/day for 52 weeks, oral Topiramate: 100mg/day or MTD for 52 weeks Flunarizine and Topiramate: 5 mg/day of flunarizine with 25-100 mg/day of topiramate for 52 weeks |
| Mansoureh, 2008 (44) | NR | NR | NR | NR | 34.1 | 19.2 | NR | NR | NR | 7.2 | Cinnarizine: 75 mg/day for 12 weeks, oral Valproate: 600 mg/day for 12 weeks, oral |
| Mathew, 1981 (45) | NR | NR | NR | NR | 34.0 | 9.5 | NR | NR | NR | NR | Propranolol: 160mg/day or MTD for 28 weeks, oral Amitriptyline: 50-75mg or MTD for 28 weeks, oral |
| Mathew, 1995 (46) | NR | NR | Abbott Laboratories | United States | 45.6 | 22.4 | 25.0 | 27.0 | NR | 6.1 | Valproate: 750 mg/day titrated over 4 weeks, maintained for 8 weeks, oral Placebo |
| Mathew, 2001 (47) | NR | NR | NR | NR | 39.6 | 17.2 | 20.8 | 43.7 | NR | 4.9 | Gabapentin: 2400 mg/day titrated over 4 weeks, maintained for 8, oral Placebo |
| Mei  2004 (48) | NR | NR | NR | Italy | 39.2 | 45.8 | NR | 19.4 | NR | 5.5 | Topiramate: 100mg/day titrated over 4 weeks maintained for 12, oral  Placebo |
| Misra, 2013 (49) | NR | NR | NR | NR | 32.0* | 22.8 | 5.0* | 4.0 | NR | 6.1 | Valproate: 500-750 mg/day for 24 weeks, oral Amitriptyline: 25-50 mg/day for 24 weeks, oral |
| Mulleners, 2020 (50) | CONQUER | NR | Eli Lilly and Company | Belgium, Canada, Czechia, France, Germany, Hungary, Japan, Korea Republic, Netherlands, Spain, United Kingdom, United States | 45.8 | 14.1 | 23.3 | 44.2 | 100.0 | 13.2 | Galcanezumab: 240 mg loading dose, 120 mg q4w for 12 weeks, SC Placebo |
| Reuter, 2018 (51) | LIBERTY | NCT03096834 | Novartis | Australia, Austria, Belgium, Czech Republic, Denmark, Finland, France, Germany, Greece, Italy, Norway, Netherlands, Spain, Sweden, Switzerland, United Kingdom | 44.4 | 18.7 | NR | 35.4 | 100.0 | 9.3 | Erenumab: 70 mg q4w for 12 weeks, SC Placebo |
| Reuter, 2022 (52) | HER-MES | NCT03828539 | Novartis | Germany | 40.7 | 14.2 | 21.9 | 34.27 | 40.7 | 10.4 | Erenumab: 140mg q4w for 24 weeks, SC Topiramate: 50–100 mg/day for 24 weeks, oral |
| Sakai, 2019 (53) | NR | NCT02630459 | Amgen, Novartis | Japan | NR | 15.8 | NR | 26.1 | 66.3 | 7.8 | Erenumab (28mg): 28 mg, q4w for 24 weeks, SC Erenumab (70mg): 70 mg, q4w for 24 weeks, SC Erenumab (140mg): 140 mg, q4w for 24 weeks, SC Placebo |
| Sakai, 2020 (54) | NR | NCT02959177 | Eli Lilly and Company | Japan | 44.1 | 15.7 | 21.4 | NR | 60.6 | 8.7 | Galcanezumab (120mg): 120 mg, q4w for 24 weeks, SC Galcanezumab (240mg): 240 mg, q4w for 24 weeks, SC Placebo |
| Sakai, 2021 (55) | NR | NCT03303079 | Otsuka Pharmaceutical | Japan, Korea | 42.8 | 13.7 | 18.7 | NR | 21.0 | 15.7 | Fremanezumab (monthly) 675mg loading dose, 225mg q4w for 12 weeks, SC Fremanezumab (quarterly): 675mg once for 12 weeks, SC Placebo |
| Sakai, 2021 (56) | NR | NCT03303092 | Otsuka Pharmaceutical | Japan, Korea | 43.5 | 15.4 | 19.9 | NR | 19.3 | 8.8 | Fremanezumab (monthly): 225 mg q4w, SC Fremanezumab (quarterly): 3 doses of 225 mg at baseline, SC Placebo |
| Sargent, 1985 (57) | NR | NR | NR | NR | 30.0 | 21.0 | 20.0 | NR | NR | NR | Propranolol: 120mg/day for 12 weeks  Valproate: 1100mg/day for 12 weeks Placebo |
| Silberstein, 2004 (58) | NR | NR | Johnson & Johnson Pharmaceutical Research and Development | NR | 40.4 | 11.3 | NR | NR | NR | 6.5 | Topiramate (50mg): 50 mg/day titrated over 8 weeks, maintained for 18 weeks, oral Topiramate (100mg): 100 mg/day, titrated over 8 weeks, maintained for 18 weeks, oral Topiramate (200mg): 200 mg/day titrated over 8 weeks, maintained for 18 weeks, oral Placebo |
| Silberstein, 2006 (59) | NR | NR | Ortho-Mcneil Neurologics | United States | 40.5 | 14.2 | NR | 35.6 | NR | 4.9 | Topiramate: 200 mg/day, titrated over 8 weeks, maintained for 12 weeks, oral Placebo |
| Silberstein, 2008 (60) | NR | NR | Novartis | United States | 40.5 | 15.3 | NR | NR | NR | NR | Oxcarbazepine: 1200 mg/day titrated over 6 weeks, maintained for 8 weeks, oral Placebo |
| Silberstein, 2009 (61) | NR | NCT00210912 | Ortho-McNeil Janssen | United States | 38.2 | NR | NR | NR | NR | NR | Topiramate: 100 mg/day titrated over 4 weeks, maintained for 12 weeks, oral Placebo |
| Silberstein, 2012 (62) | NR | NCT00772031 | National Institute of Neurological Disorders and Stroke | United States | 42.0* | 10.0 | NR | NR | NR | 18.0 | Propranolol: total dose of 240 mg/day for 6 months, oral Placebo |
| Silberstein, 2013 (63) | NR | NCT00742209 | GlaxoSmithKline | Canada, United States | 39.2 | 18.0 | NR | NR | NR | 9.2 | Gabapentin (1200mg): 1200 mg/day for 20 weeks, oral Gabapentin (1800mg): 1800 mg/day for 20 weeks, oral Gabapentin (2400mg): 2400 mg/day for 20 weeks, oral Gabapentin (3000mg): 3000 mg/day for 20 weeks, oral Placebo |
| Silberstein, 2017 (64) | NR | NCT02621931 | Teva Pharmaceuticals | Canada, Czechia, Finland, Israel, Poland, Russia, Spain, United States, | 40.7 | 12.3 | 19.9 | NR | 21.2 | 16.2 | Fremanezumab (quarterly): 675 mg q8w for 12 weeks, SC Fremanezumab (monthly): 675 mg loading dose, 225 q4w for 12 weeks, SC Placebo |
| Skljarevski, 2018 (65) | EVOLVE-2 | NCT02614196 | Eli Lilly and Company | Argentina, Czechia, Germany, Israel, Korea, Mexico, Netherlands, Spain, Taiwan, United Kingdom, United States | 41.9 | 14.6 | 20.6 | NR | 66.2 | 9.1 | Galcanezumab (120mg): 120 mg q4w 24 weeks, SC Galcanezumab (240mg): 240 mg, q4w for 24 weeks, SC Placebo |
| Skljarevski, 2018 (66) | NR | NCT02163993 | Eli Lilly and Company | United States | 40.2 | 17.1 | NR | NR | NR | 6.7 | Galcanezumab (5mg): 5 mg, q4w for 12 weeks, SC Galcenezumab (50mg): 50 mg, q4w for 12 weeks SC Galcanezumab (120mg): 120 mg, q4w for 12 weeks, SC Galcanezumab (300mg): 300 mg, q4w for 12 weeks, SC Placebo |
| Sorensen, 1991 (67) | NR | NR | Janssen Pharmaceuticals | Denmark | 42.0 | 20.8 | 17.0* | 26.2 | 54.4 | NR | Flunarizine: 10 mg/day for 16 weeks, oral Metoprolol: 200 mg/day for 16 weeks, oral |
| Sudilovsky, 1987(68) | NR | NR | NR | United States | 39.3 | 24.5 | 20.7 | NR | NR | 5.3 | Nadalol (80mg): 80 mg/day for 12 weeks, oral Nadolol (160mg): 160 mg/day for 12 weeks, oral Propranolol: 160 mg/day for 12 weeks, oral |
| Sun, 2016 (69) | NR | NCT01952574 | Amgen | Canada, United States, Denmark, Finland, Germany, Norway, Sweden, Portugal | 41.1 | 19.5 | 20.4 | NR | 57.6 | 8.7 | Erenumab (7mg): 7 mg, q4w for 12 weeks, SC Erenumab (21mg): 21 mg, q4w for 12 weeks, SC Erenumab (70mg): 70 mg, q4w for 12 weeks, SC Placebo |
| Takeshima, 2021 (70) | NR | NCT03812224 | Amgen | Japan | 44.4 | 13.0 | NR | NR | 77.4 | 12.1 | Erenumab: 70 mg q4w for 24 weeks, SC Placebo |
| Tepper, 2017 (71) | NR | NCT02066415 | Amgen | Canada, Czech Republic, Denmark, Finland, Germany, Norway, Poland, Sweden, UK, United States | 42.1 | 17.2 | 21.7 | 41.4 | NR | 18 | Erenumab (70mg): 70mg q4w for 12 weeks, SC  Erenumab (140mg): 140mg q4w for 12 weeks, SC  Placebo |
| van de Ven, 1997 (72) | NR | NR | Merck KGaA | Belgium, France, The Netherlands, Spain | 38.7 | 17.7 | NR | 22.6 | NR | 5.5 | Bisoprolol (5mg): 5 mg/day for 12 weeks, oral Bisoprolol (10mg): 10 mg/day for 12 weeks, oral Placebo |
| Wang, 2021 (73) | EMPOwER | NCT03333109 | Novartis | NR | 37.5 | 18.1 | 11.7 | 69.9 | 53.2 | 9.3 | Erenumab (70mg): 70 mg, q4w for 12 weeks, SC Erenumab (140mg): 140 mg, q4w for 12 weeks, SC Placebo |

#

# Supplement 5 – Risk of bias judgements for mean monthly migraine days


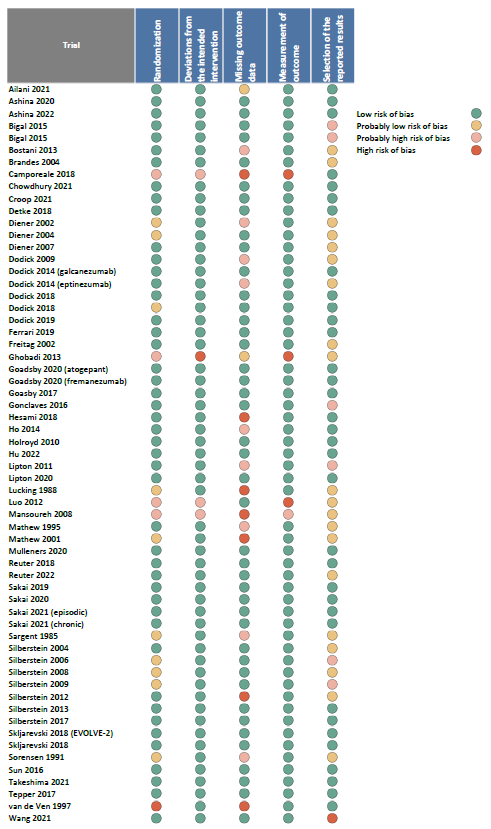


# Supplement 6 – Risk of bias judgements for adverse events leading to discontinuation


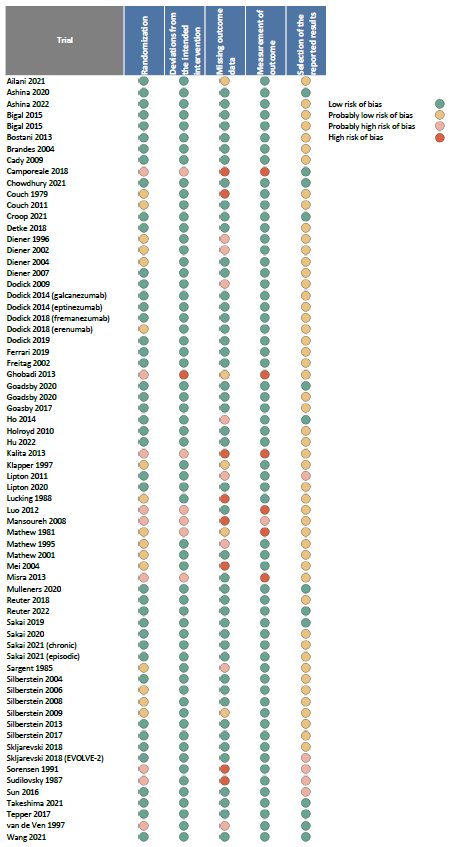


# Supplement 7 – Comparisons and GRADE ratings for network meta-analysis of 50% or more reduction in monthly migraine days


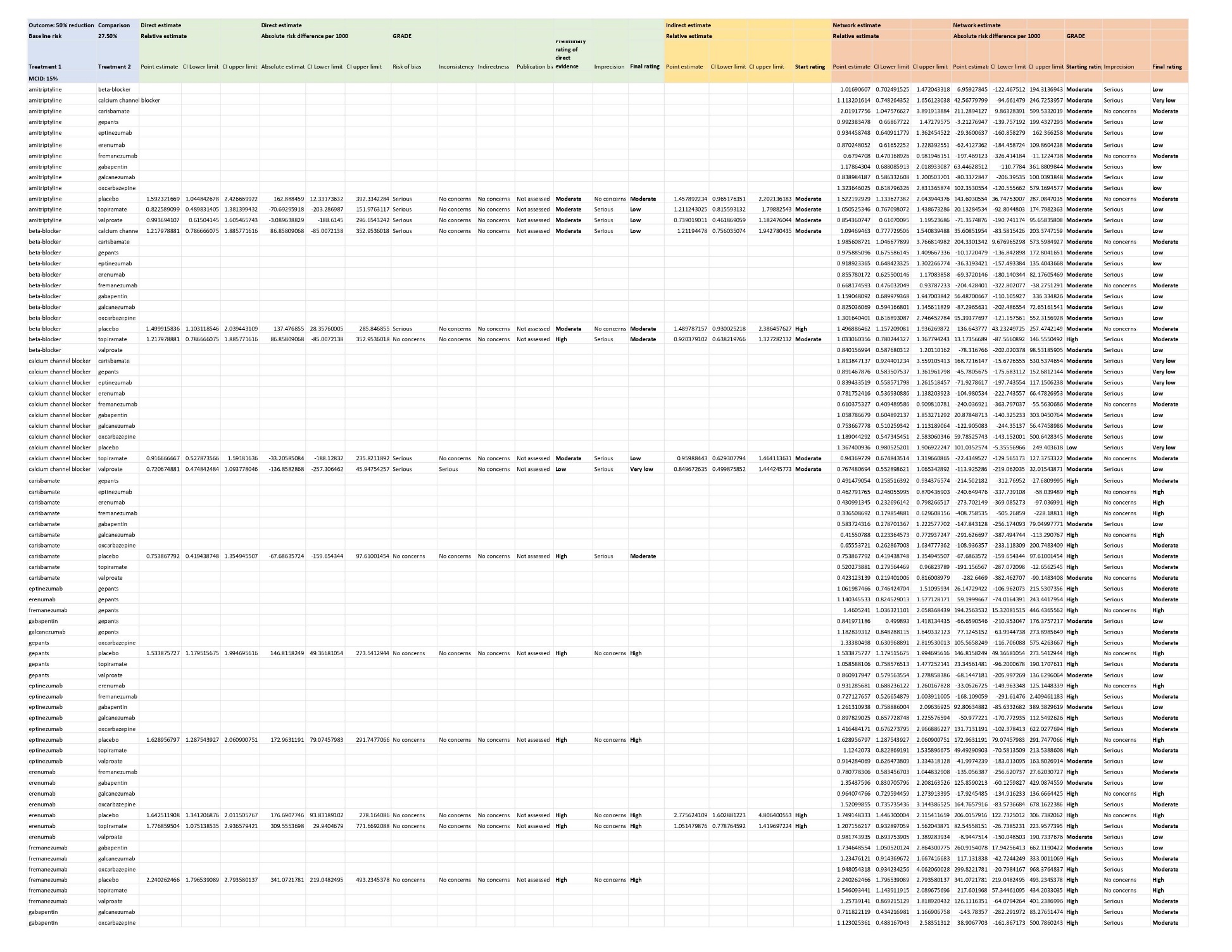


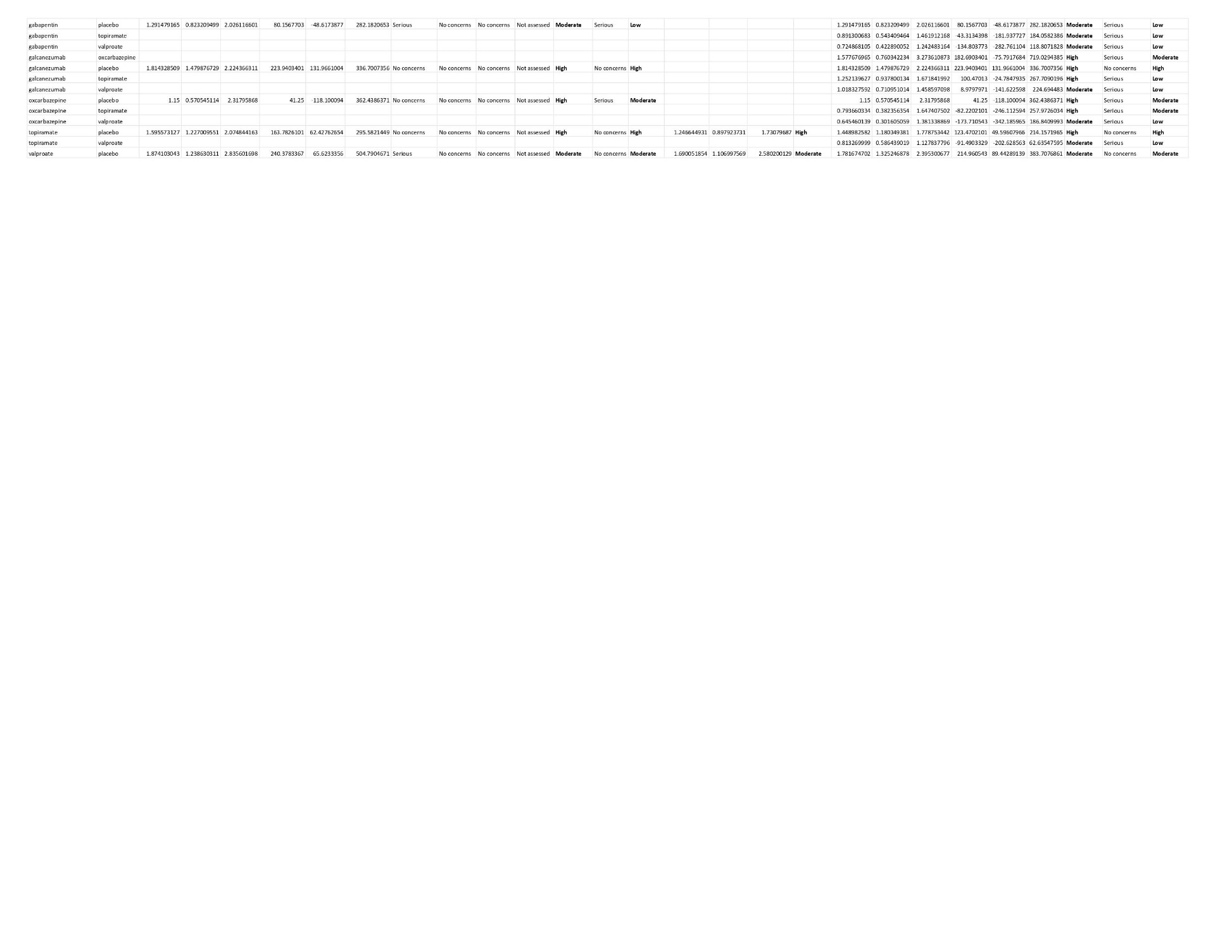


Full table also available at: <https://osf.io/g3u26>

# Supplement 8 – Pairwise meta-analyses for 50% or more reduction in monthly migraine days


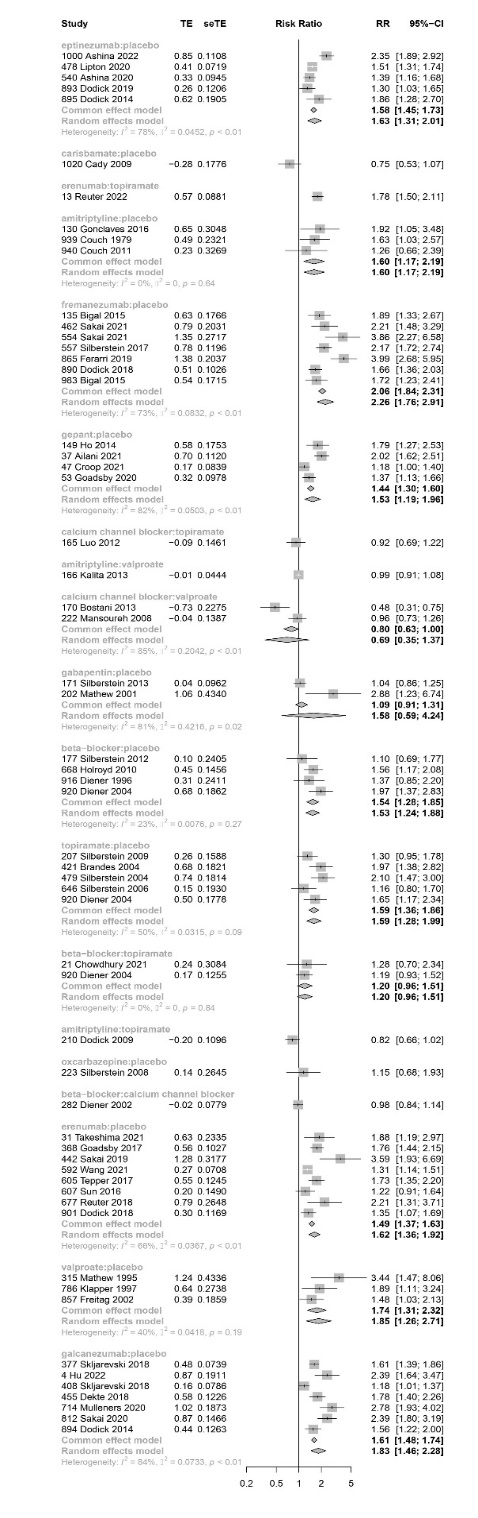


# Supplement 9 – Node split plots for 50% or more reduction in monthly migraine days


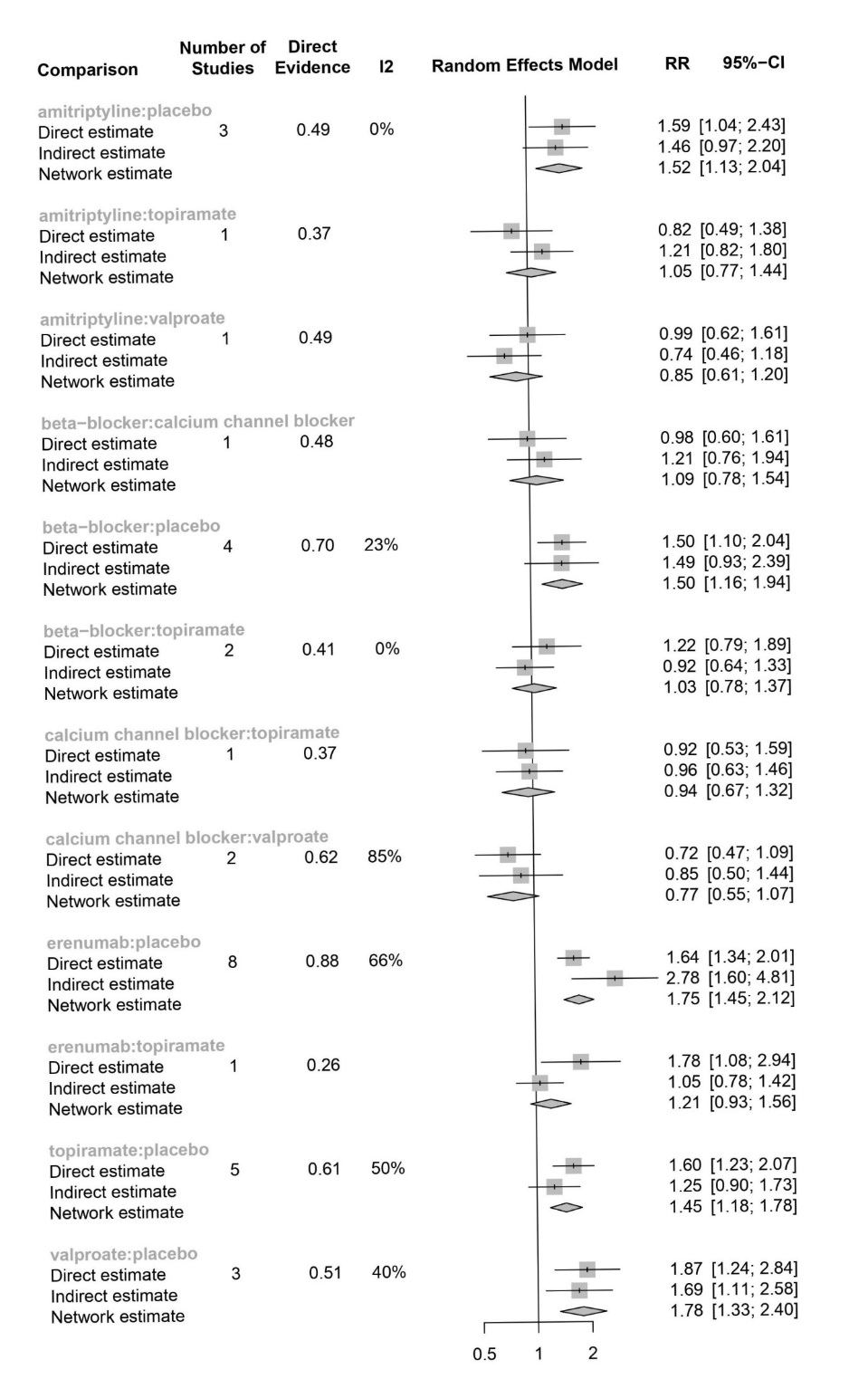


# Supplement 10 – Secondary analysis for 50% or more reduction in monthly migraine days (all monoclonal antibodies are grouped in one node)


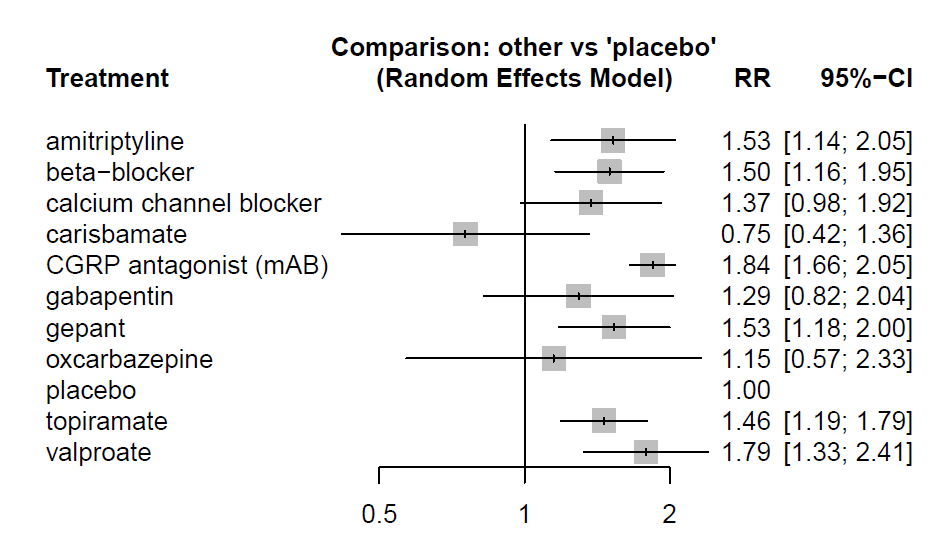


# Supplement 11 – Secondary analysis for 50% or more reduction in monthly migraine days (restricted trials to those that investigated recommended therapeutic doses of drugs)


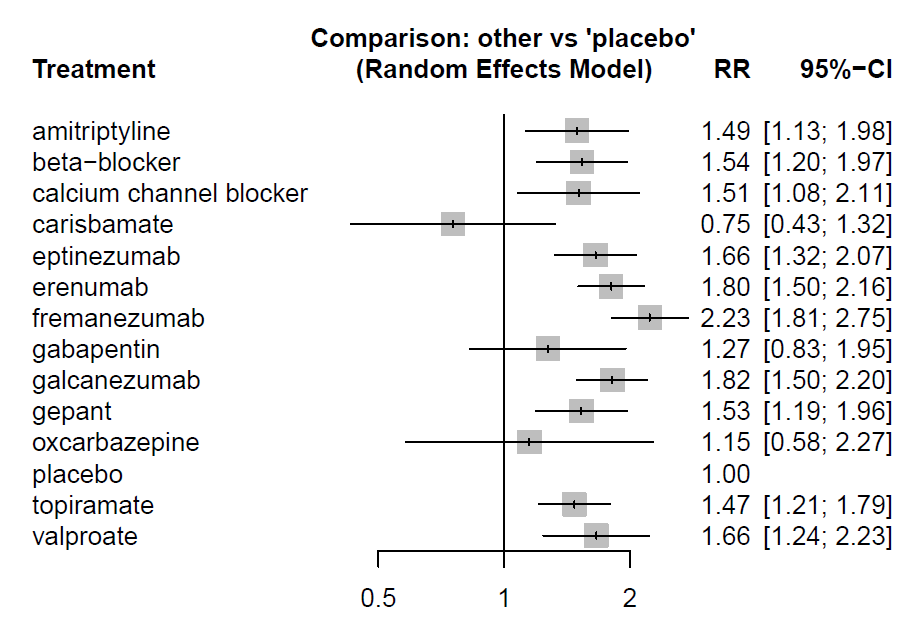


# Supplement 12 – Secondary analysis for 50% or more reduction in monthly migraine days (gepants grouped in separate nodes)


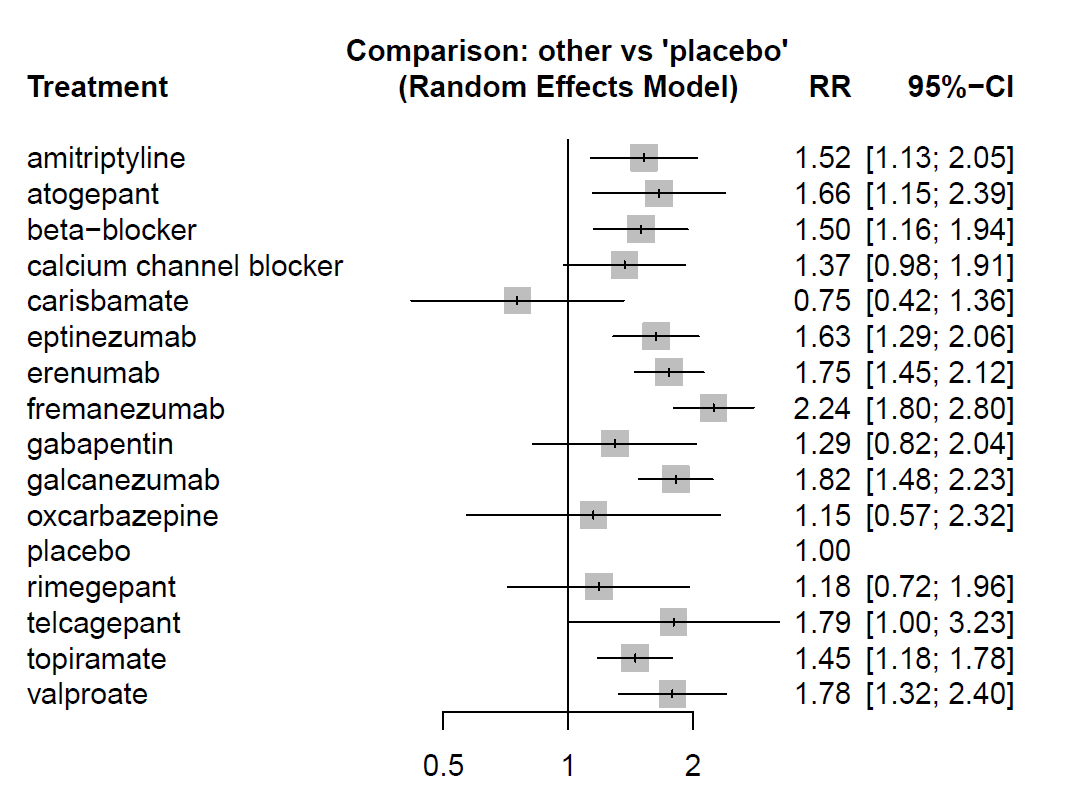


# Supplement 13 – Secondary analysis comparing the effects of telcagepant with other gepants


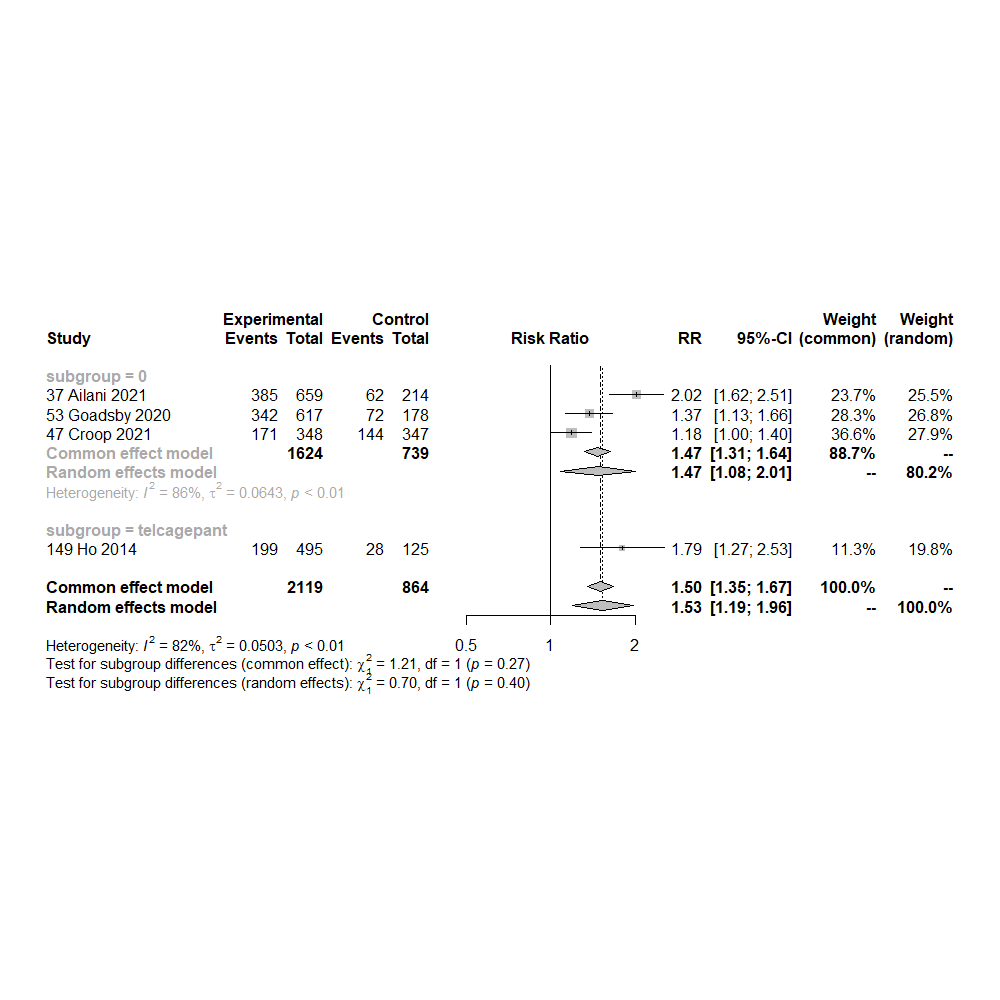


# Supplement 14 – Subgroup analyses for 50% or more reduction in monthly migraine days

For subgroup analyses based on baseline monthly migraine days and proportion of patients that had previously used prophylactic drugs, for each comparison against placebo, we calculated the median monthly migraine days and median proportion of patients who had previously used prophylactic drugs across trials, and performed subgroup analyses comparing trials with mean baseline monthly migraine days or proportion of patients who had previously used prophylactic drugs above or equal to/below the median.

| Drug | Risk of bias | | | | | |
| --- | --- | --- | --- | --- | --- | --- |
|  | **Low** | | | **High** | | |
|  | **Number of trials (participants)** | **RR (95% CI)** | **I^2^ (%)** | **Number of trials (participants)** | **RR (95% CI)** | **I^2^ (%)** |
| Amitriptyline | NA |  |  | 3 (389) | 1.60 (1.17, 2.19) | 0 |
| Beta-blocker | 1 (286) | 1.97 (1.37, 2.83) | NA | 2 (299) | 1.23 (0.88, 1.72) | 0 |
| Calcium channel blockers | NA |  |  | NA |  |  |
| Eptinezumab | 4 (3416) | 1.59 (1.24, 2.05) | 83 | 1 (143) | 1.86 (1.28, 2.70) | NA |
| Erenumab | 8 (4467) | 1.62 (1.36, 1.92) | 66 | NA |  |  |
| Fremanezumab | 6 (3973) | 2.38 (1.79, 3.16) | 76 | 1 (261) | 1.72 (1.23, 2.41) | NA |
| Gabapentin | 1 (474) | 1.04 (0.86, 1.25) | NA | 1 (87) | 2.88 (1.23, 6.74) | NA |
| Galcanezumab | 7 (4043) | 1.83 (1.46, 2.28) |  | NA |  |  |
| Gepant | 3 (2363) | 1.47 (1.08, 2.01) | 86 | 1 (625) | 1.79 (1.27, 2.53) | NA |
| Topiramate | 3 (1361) | 1.9 (1.55, 2.32) | 0 | 2 (539) | 1.24 (0.98, 1.58) | 0 |
| Valproate | 1 (234) | 1.48 (1.03, 2.13) | 0 | 2 (276) | 2.32 (1.33, 4.05) | 26 |
|  | **Monthly migraine days** | | | | | |
|  | **≤ median** | | | **> median** | | |
|  | **Number of trials (participants)** | **RR (95% CI)** | **I^2^ (%)** | **Number of trials (participants)** | **RR (95% CI)** | **I^2^ (%)** |
| Amitriptyline | 1 (118) | 1.92 (1.05, 3.48) | 28.1 | NA |  |  |
| Beta-blocker | 2 (419) | 1.7 (1.19, 2.41) | 30 | 1 (166) | 1.1 (0.69, 1.77) | NA |
| Calcium channel blockers | NA |  |  | NA |  |  |
| Eptinezumab | 3 (1899) | 1.81 (1.31, 2.51) | 85 | 2 (1660) | 1.45 (1.27, 1.65) | 12 |
| Erenumab | 4 (2429) | 1.67 (1.15, 2.44) | 76 | 4 (2038) | 1.62 (1.29, 2.05) | 61 |
| Fremanezumab | 4 (2287) | 2.55 (1.61, 4.03) | 85 | 3 (1947) | 2.05 (1.72, 2.43) | 0 |
| Gabapentin | 1 (87) | 2.88 (1.23, 6.74) | NA | 1 (474) | 1.04 (0.86, 1.25) | NA |
| Galcanezumab | 4 (1581) | 1.76 (1.24, 2.49) | 88 | 3 (2462) | 1.92 (1.43, 2.59) | 73 |
| Gepant | 2 (1668) | 1.66 (1.13, 2.42) | 85 | 2 (1315) | 1.42 (0.95, 2.12) | 78 |
| Topiramate | 2 (636) | 1.4 (0.99, 1.97) | 44 | 2 (936) | 2.04 (1.58, 2.62) | 0 |
| Valproate | 1 (234) | 1.48 (1.03, 2.13) | NA | 1 (105) | 3.44 (1.47, 8.06) | NA |
|  | **Previous prophylactic drugs** | | | | | |
|  | **≤ median** | | | **> median** | | |
|  | **Number of trials (participants)** | **RR (95% CI)** | **I^2^ (%)** | **Number of trials (participants)** | **RR (95% CI)** | **I^2^ (%)** |
| Amitriptyline | NA |  |  | NA |  |  |
| Beta-blocker | NA |  |  | NA |  |  |
| Calcium channel blockers | NA |  |  | NA |  |  |
| Eptinezumab | 1 (588) | 1.3 (1.03, 1.65) | NA | 1 (868) | 2.35 (1.89, 2.92) | NA |
| Erenumab | 4 (3050) | 1.5 (1.28, 1.76) | 62 | 4 (1417) | 1.96 (1.26, 3.03) | 74 |
| Fremanezumab | 4 (2840) | 2.21 (1.64, 2.97) | 70 | 3 (1394) | 2.33 (1.39, 3.89) | 82 |
| Gabapentin | NA |  |  | NA |  |  |
| Galcanezumab | 3 (1892) | 2.02 (1.52, 2.7) | 76 | 2 (1547) | 2.17 (1.4, 3.37) | 75 |
| Gepant | 2 (1415) | 1.51 (1.17, 1.94) | 45 | 1 (873) | 2.02 (1.62, 2.51) | NA |
| Topiramate | NA |  |  | NA |  |  |
| Valproate | 1 (234) | 1.48 (1.03, 2.13) | NA | 1 (171) | 1.89 (1.11, 3.24) | NA |

# Supplement 15 – Network diagram for monthly migraine days

Each node represents a drug that has been tested in trials. The edges represent direct comparisons of the drugs in trials. The size of the nodes is proportional to the number of patients that have received that drug, and the thickness of the edges is proportional to the number of trials.


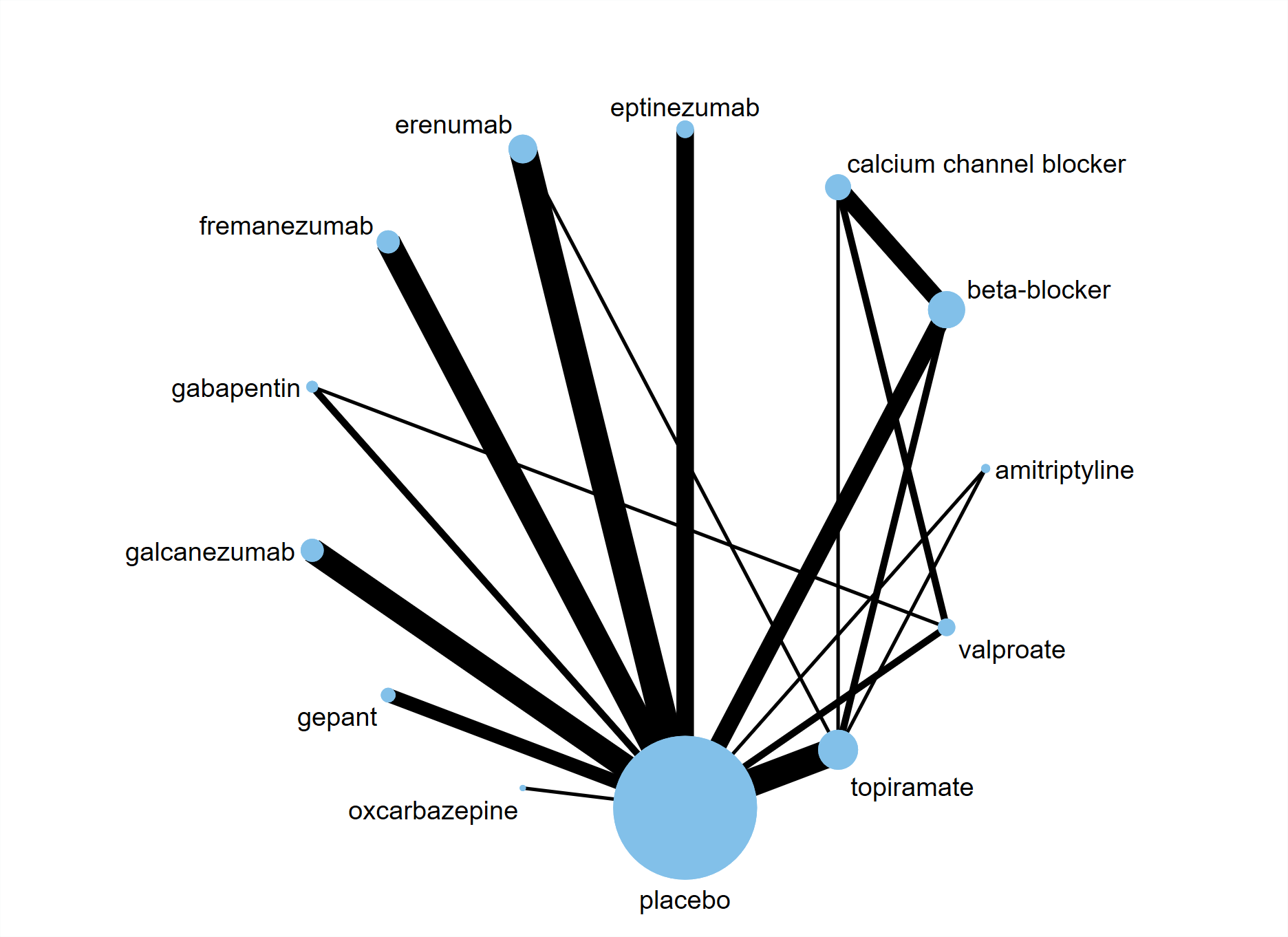


# Supplement 16 – Comparisons and GRADE ratings for network meta-analysis of monthly migraine days
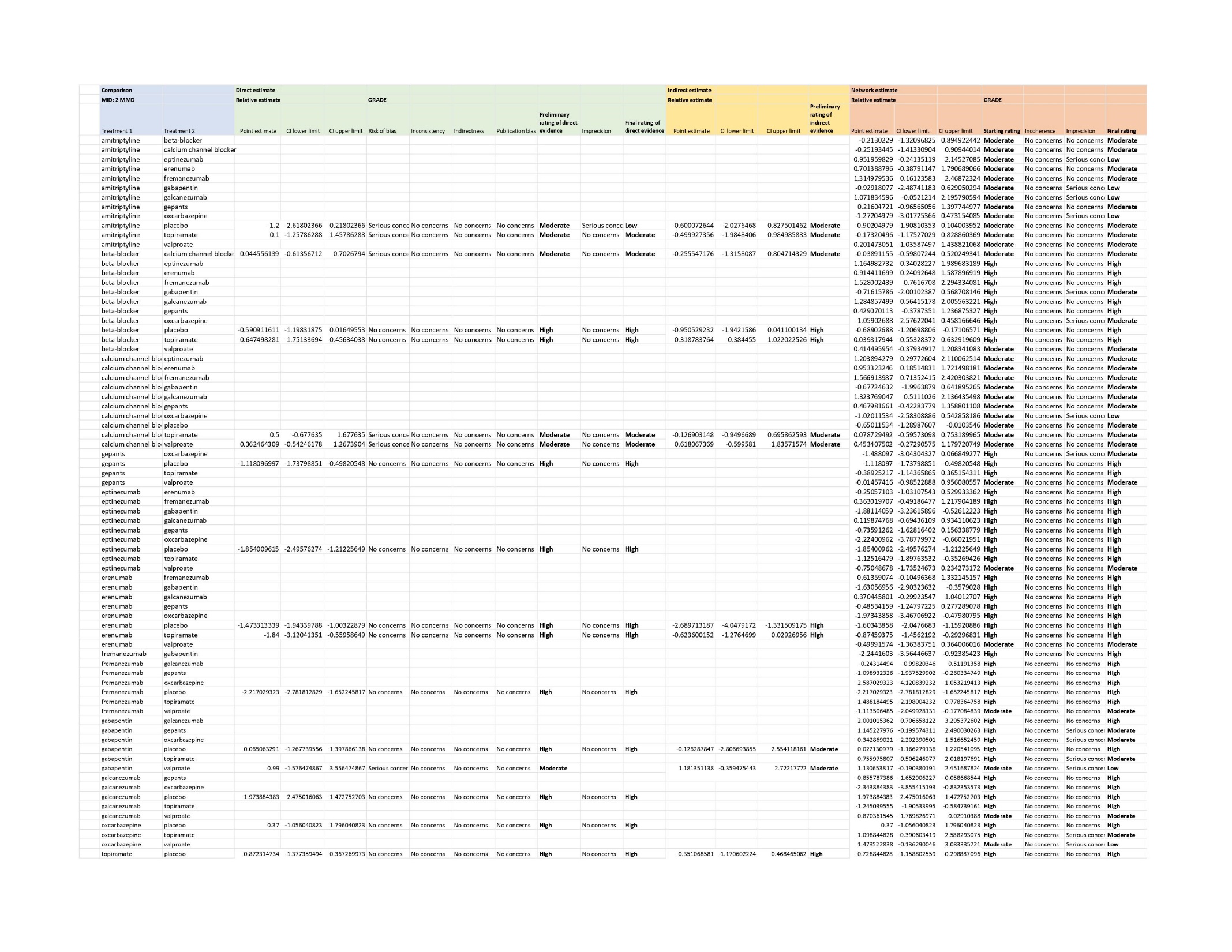

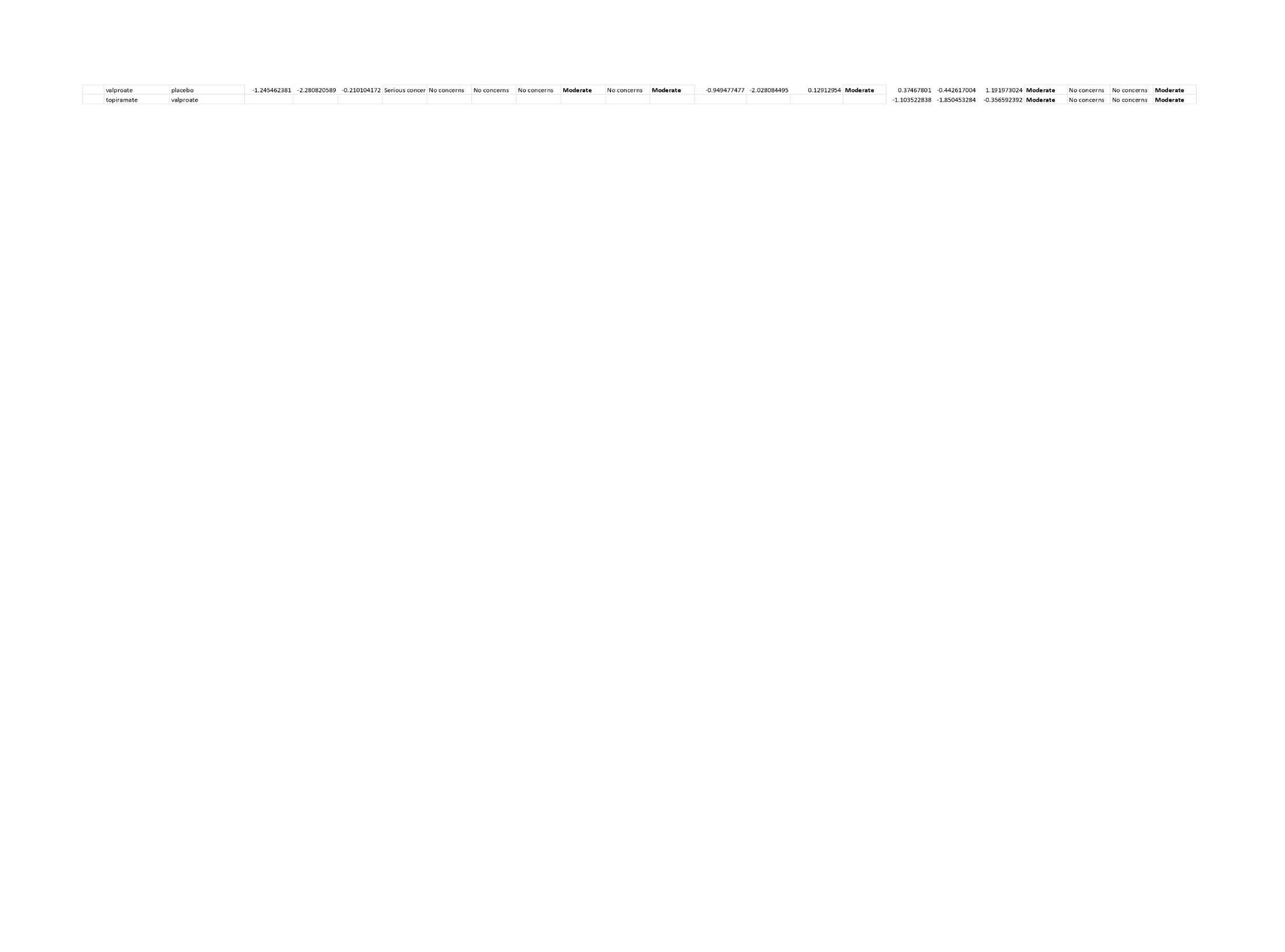


Full table also available at: <https://osf.io/g3u26>

# Supplement 17 – Pairwise meta-analyses for monthly migraine days


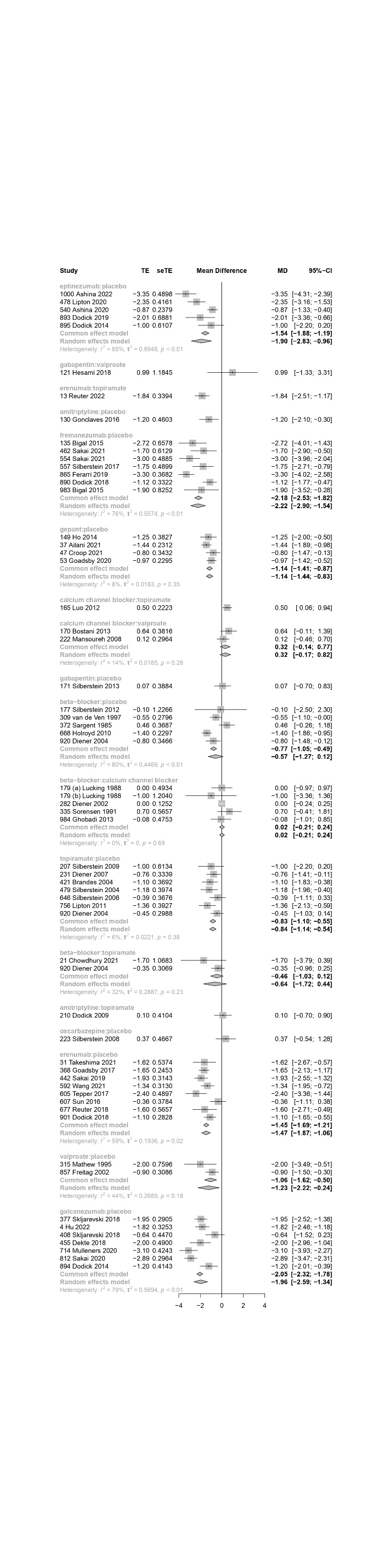


# Supplement 18 – Node split plots for monthly migraine days


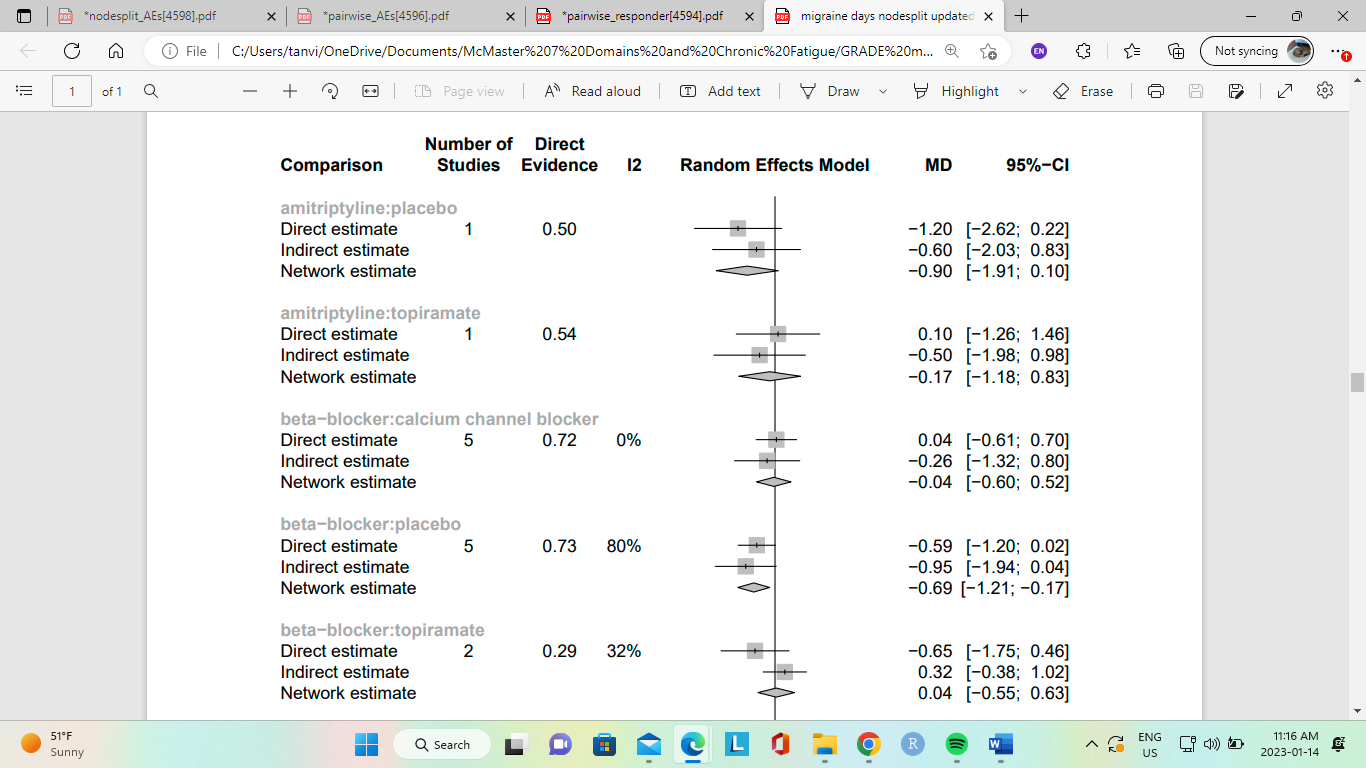

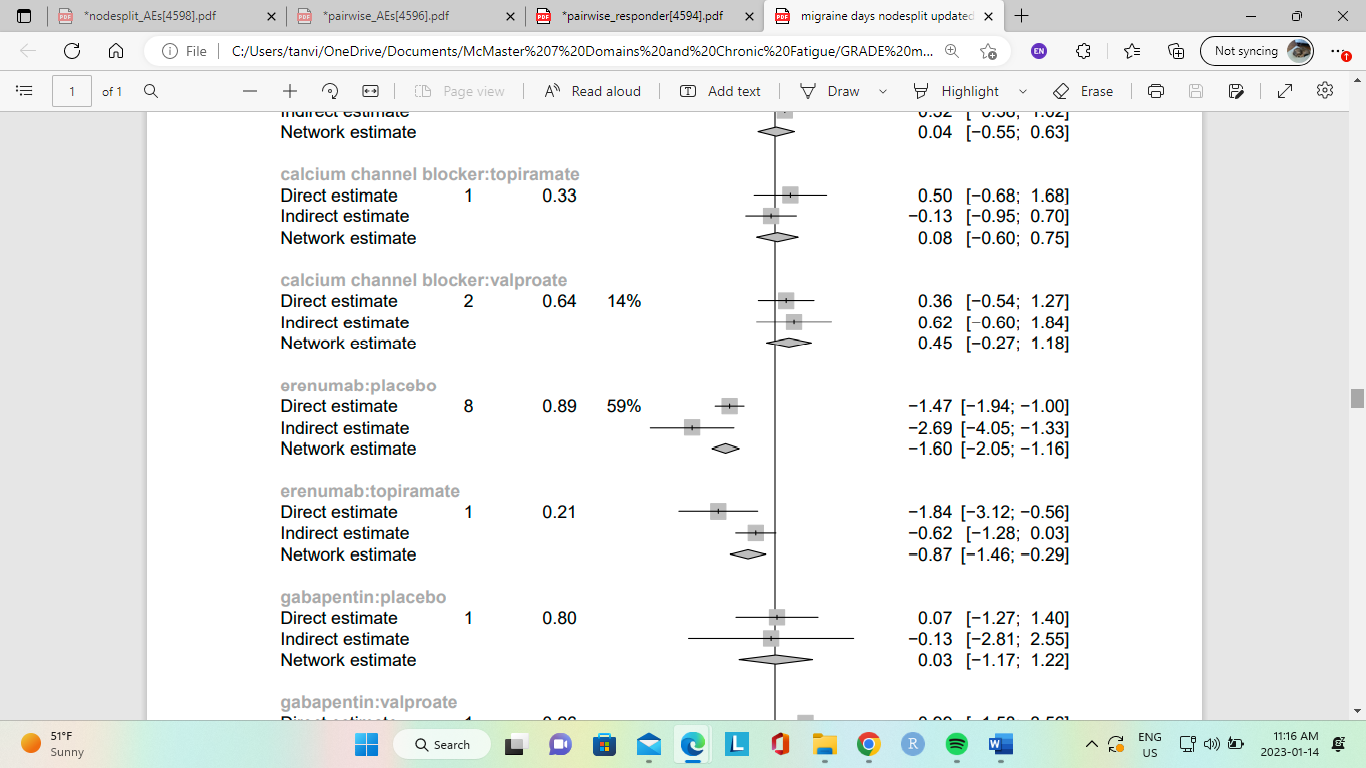

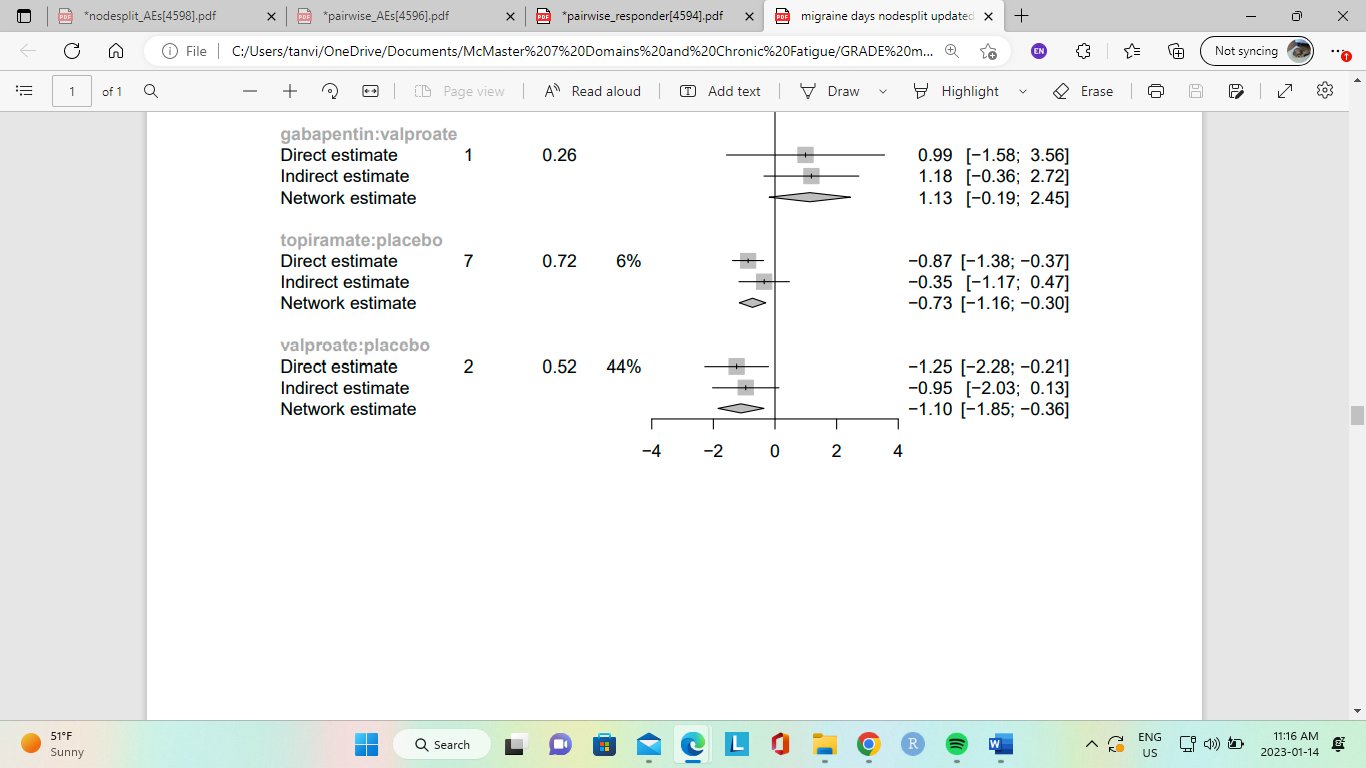


# Supplement 19 – Network diagram for adverse events leading to discontinuation

Each node represents a drug that has been tested in trials. The edges represent direct comparisons of the drugs in trials. The size of the nodes is proportional to the number of patients that have received that drug, and the thickness of the edges is proportional to the number of trials.


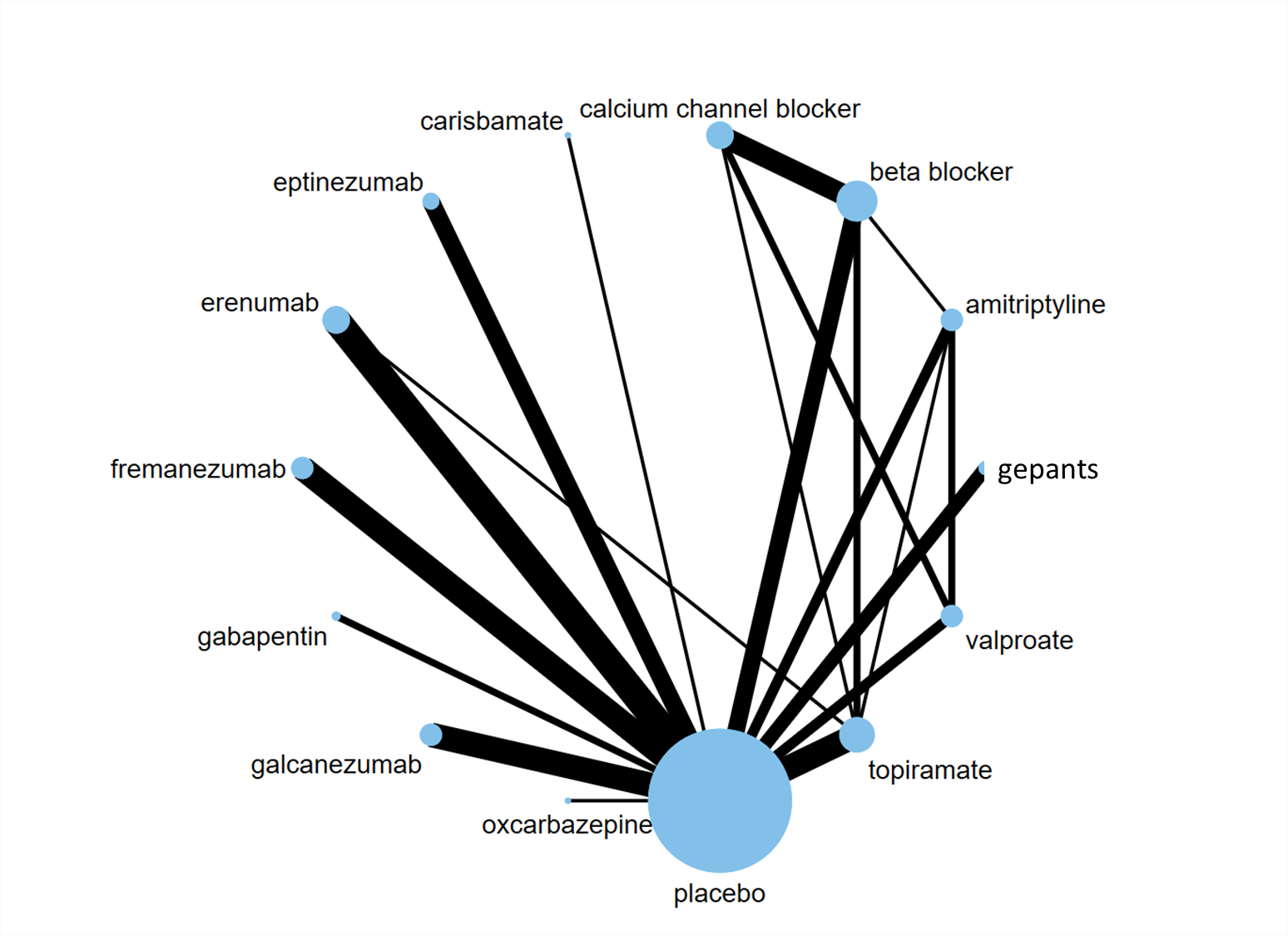


#
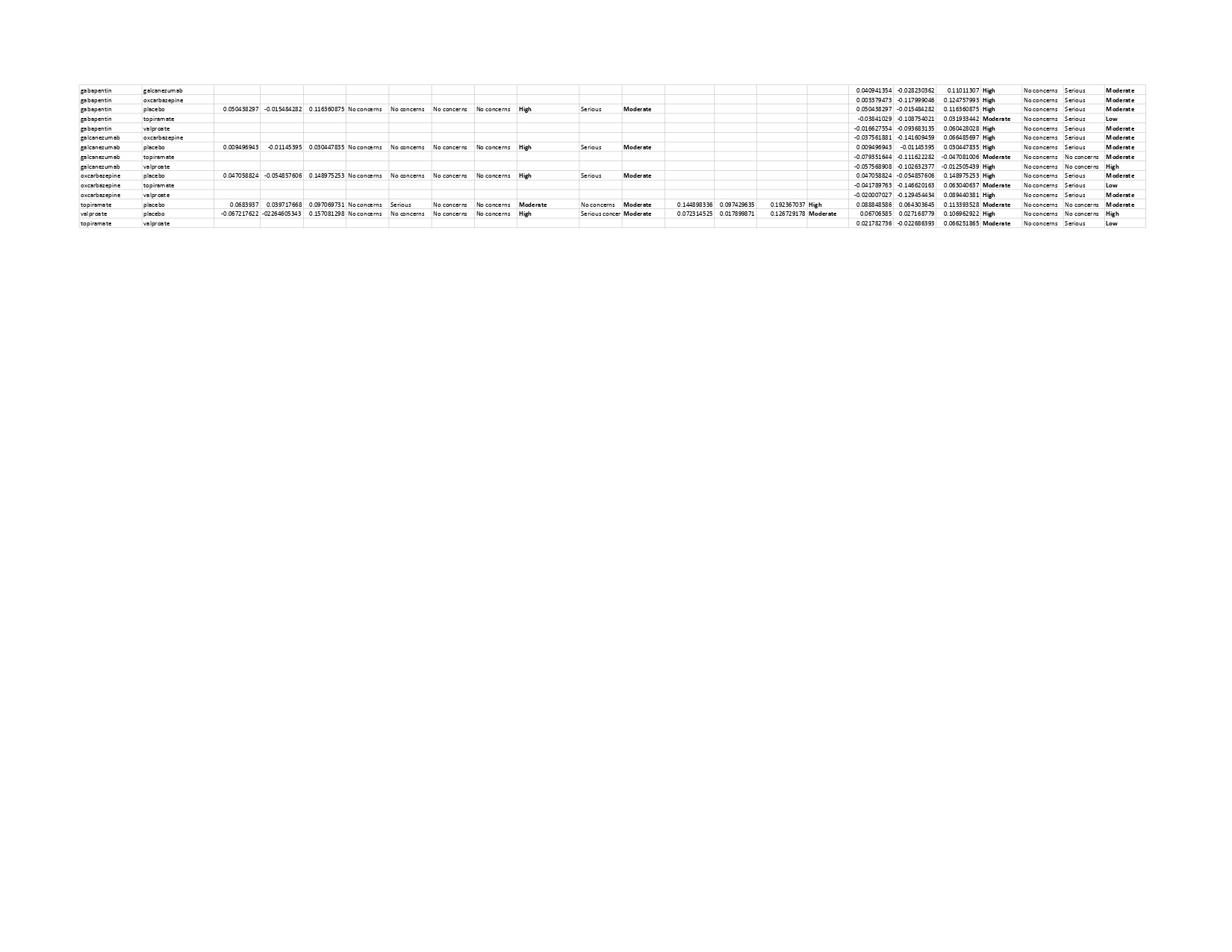
Supplement 20 – Comparisons and GRADE ratings for network meta-analysis of adverse events leading to discontinuation
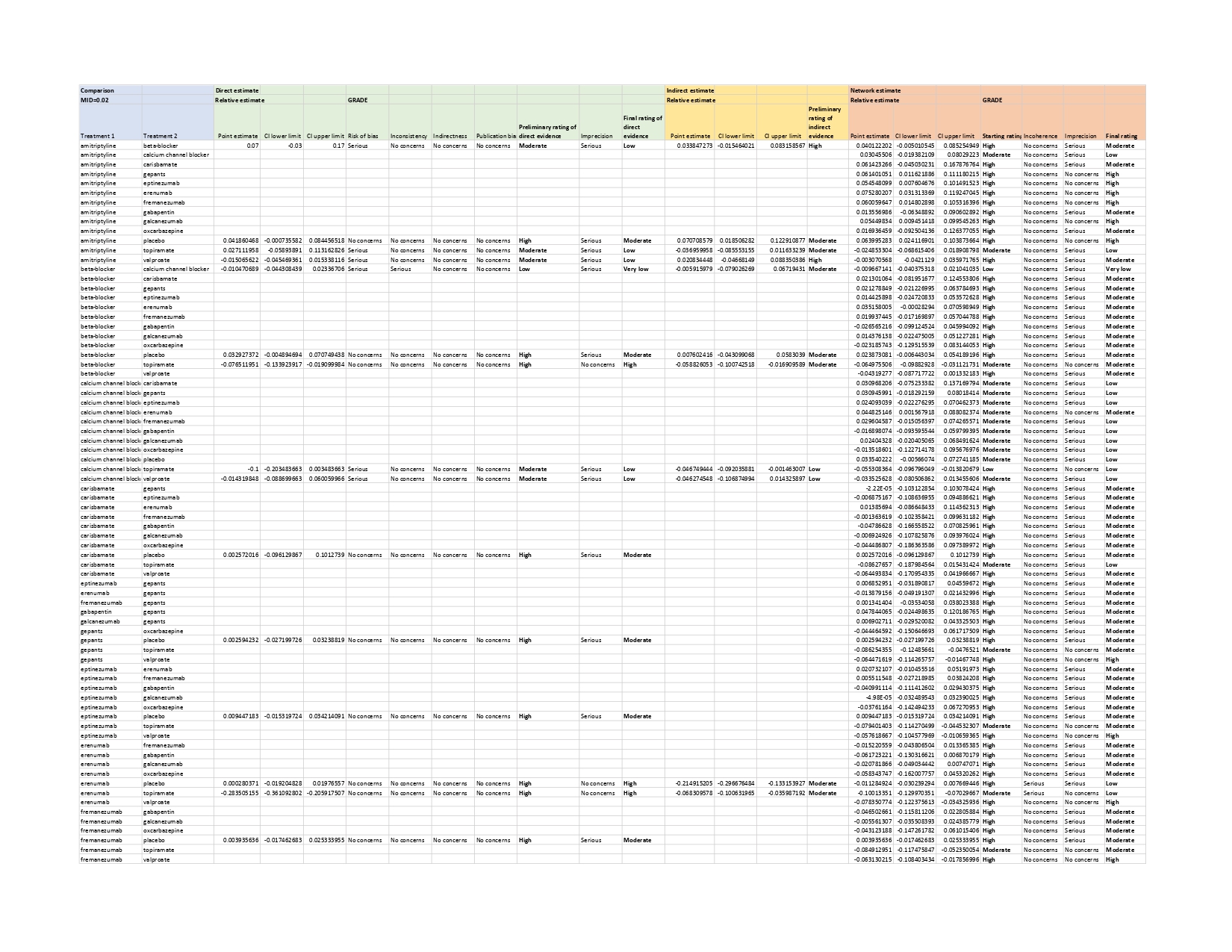


Full table also available at: <https://osf.io/g3u26>

# Supplement 21 – Pairwise meta-analyses for adverse events leading to discontinuation


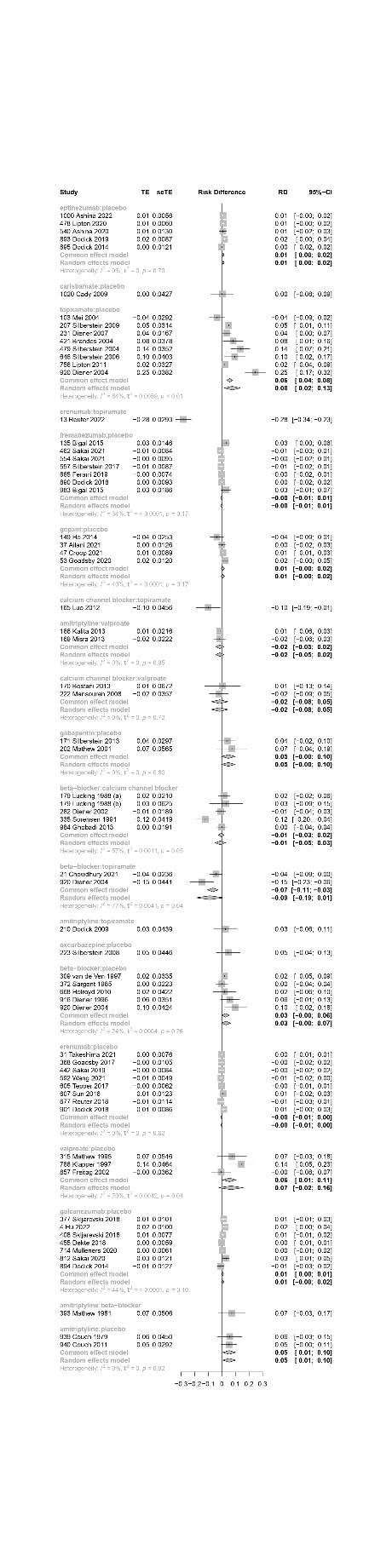


# Supplement 22 – Node split plots for adverse events leading to discontinuation


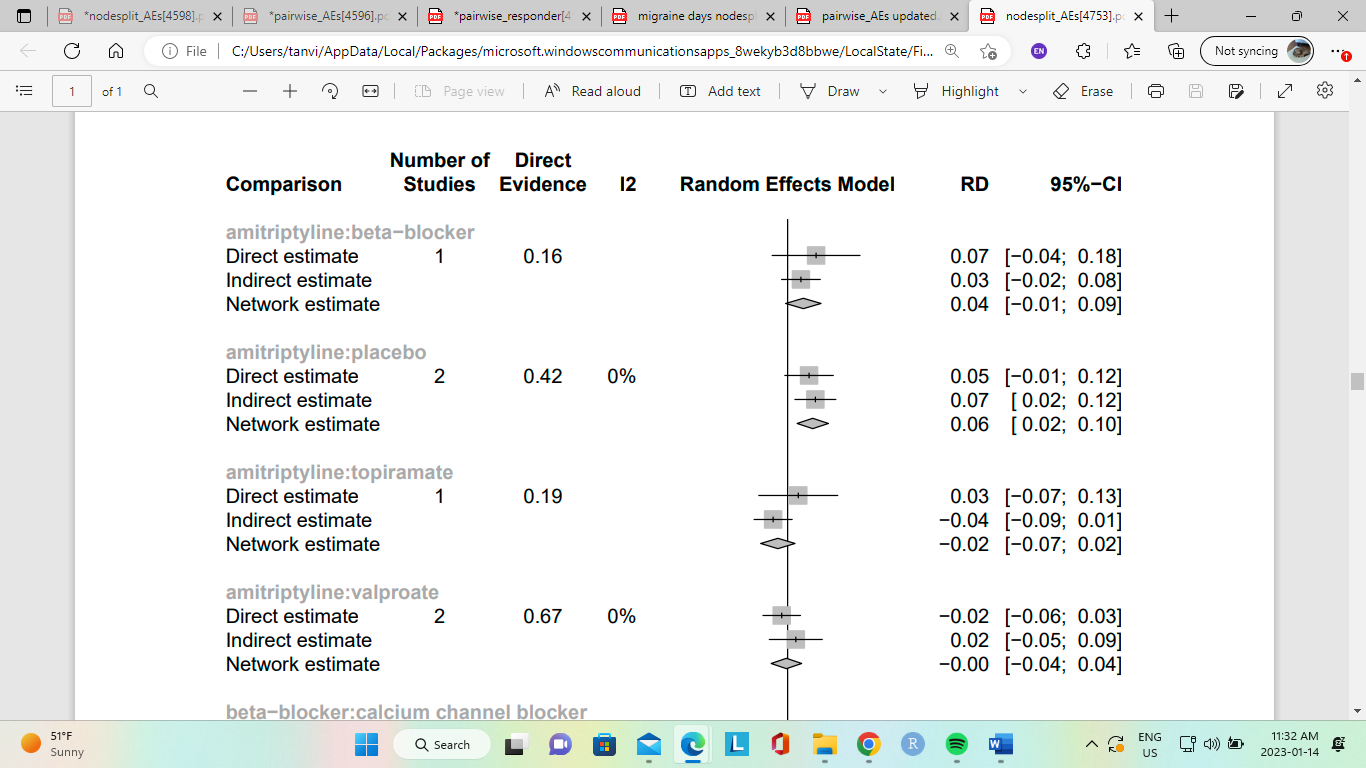

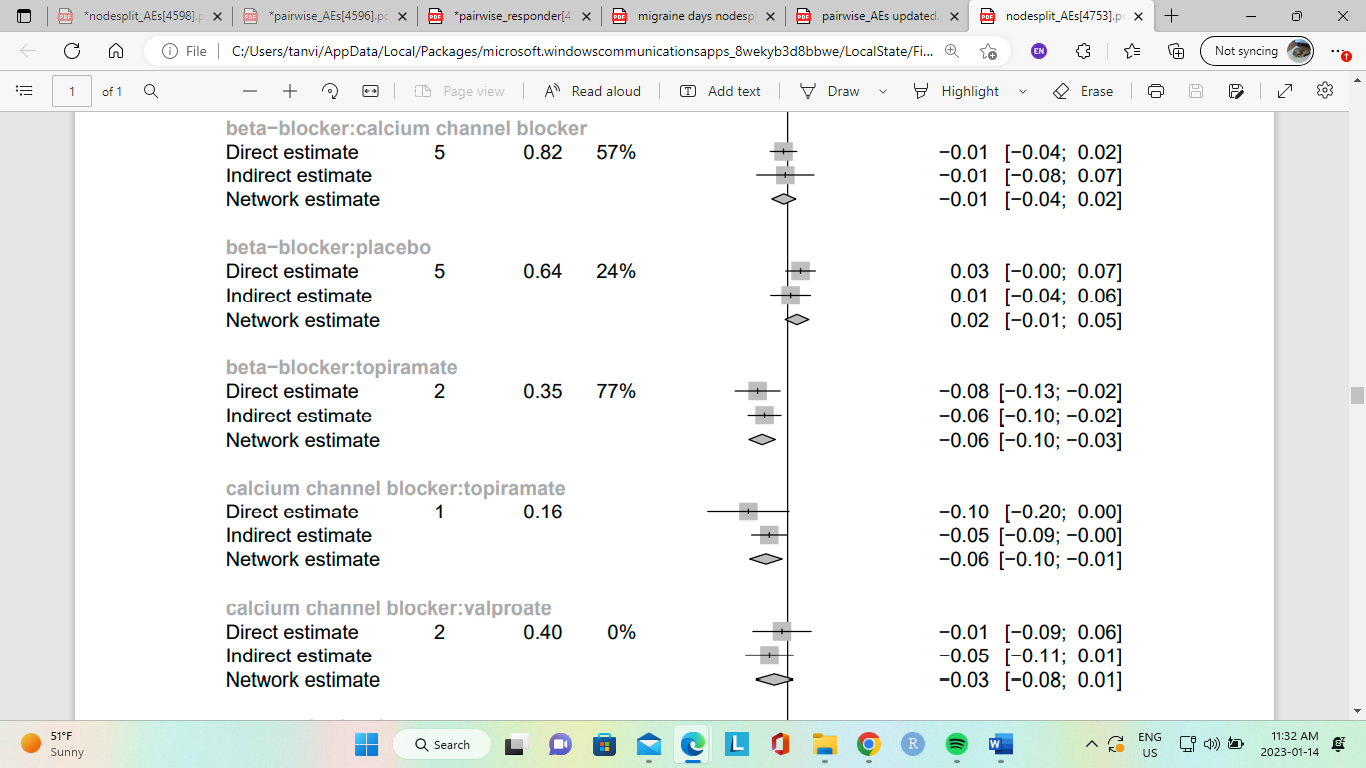


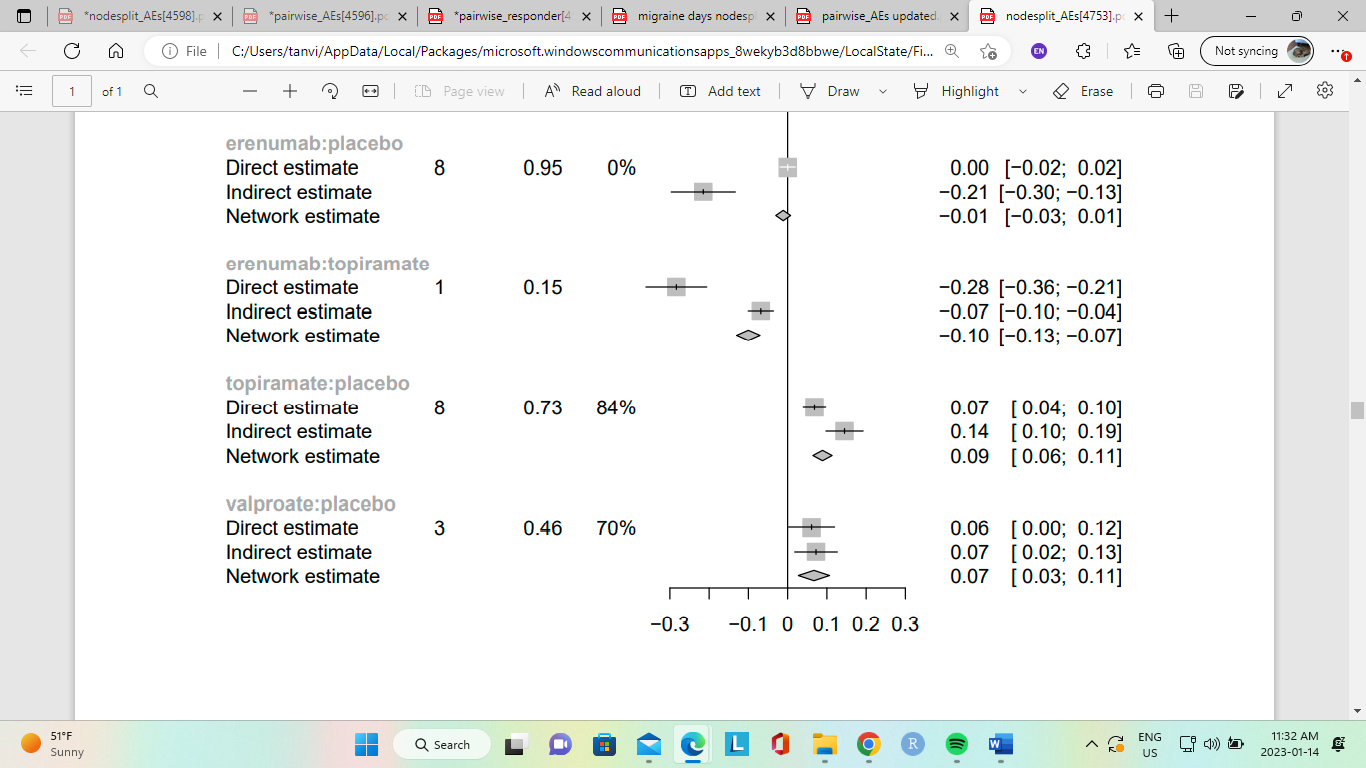


# Supplement 23 – Secondary analysis for adverse events leading to discontinuation (all monoclonal antibodies are grouped in one node)


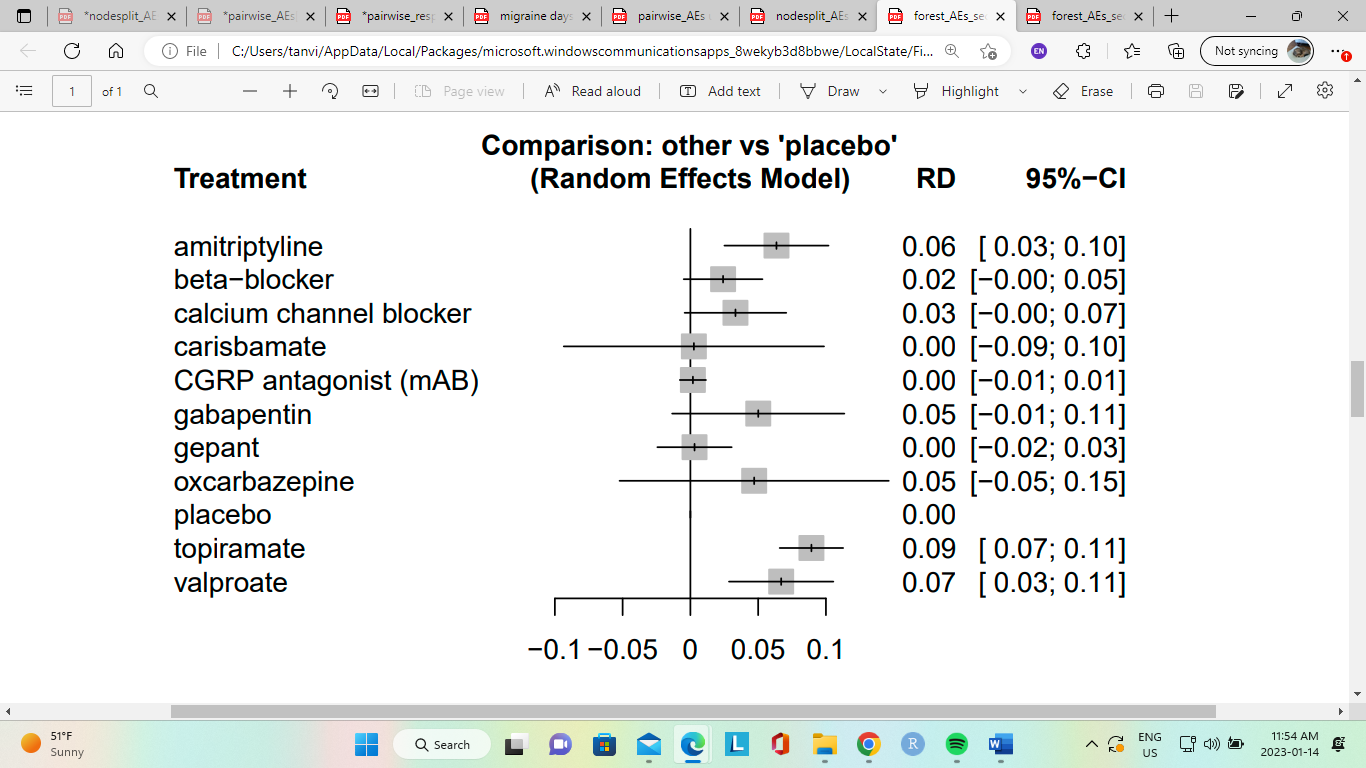


# Supplement 24 – Secondary analysis for adverse events leading to discontinuation (restricted trials to those that investigated recommended therapeutic doses of drugs)


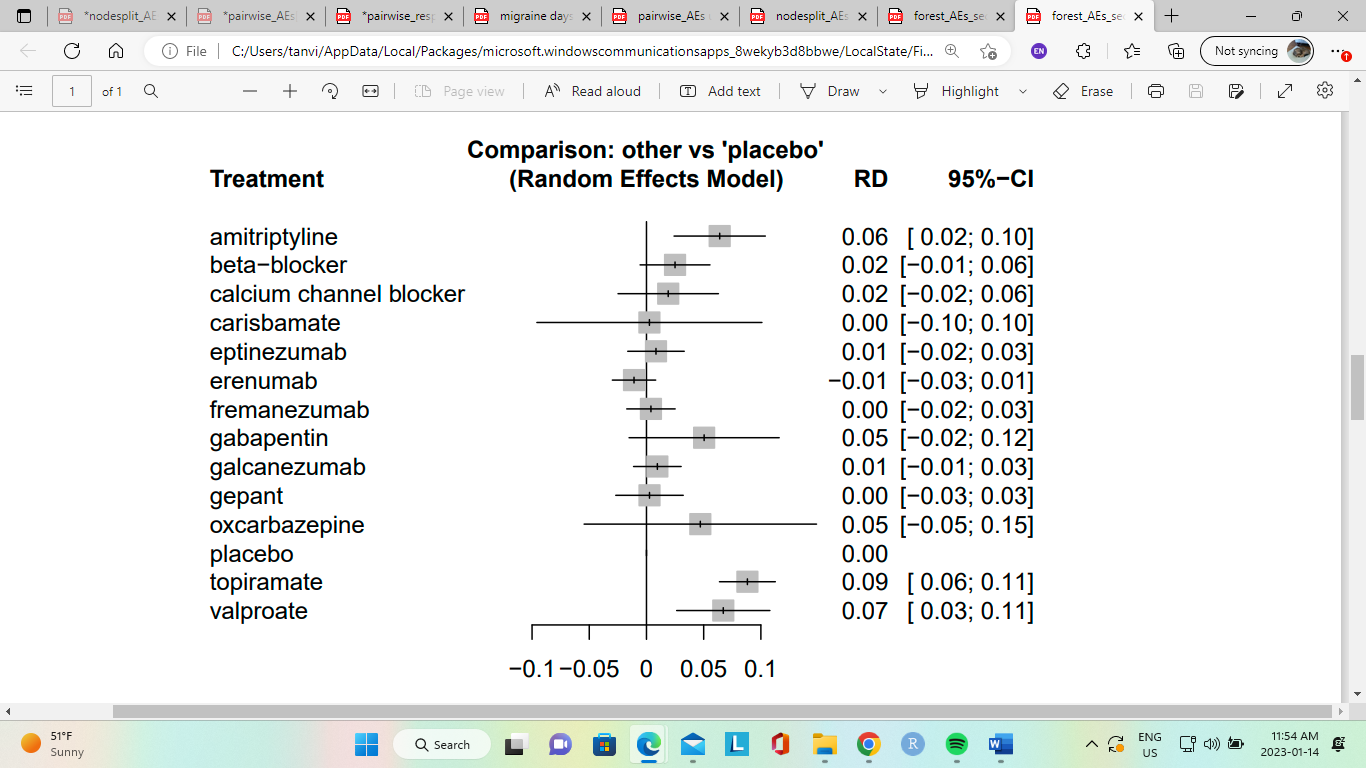


# Supplement 25 – Secondary analysis for adverse events leading to discontinuation (each gepant grouped in a separate node)


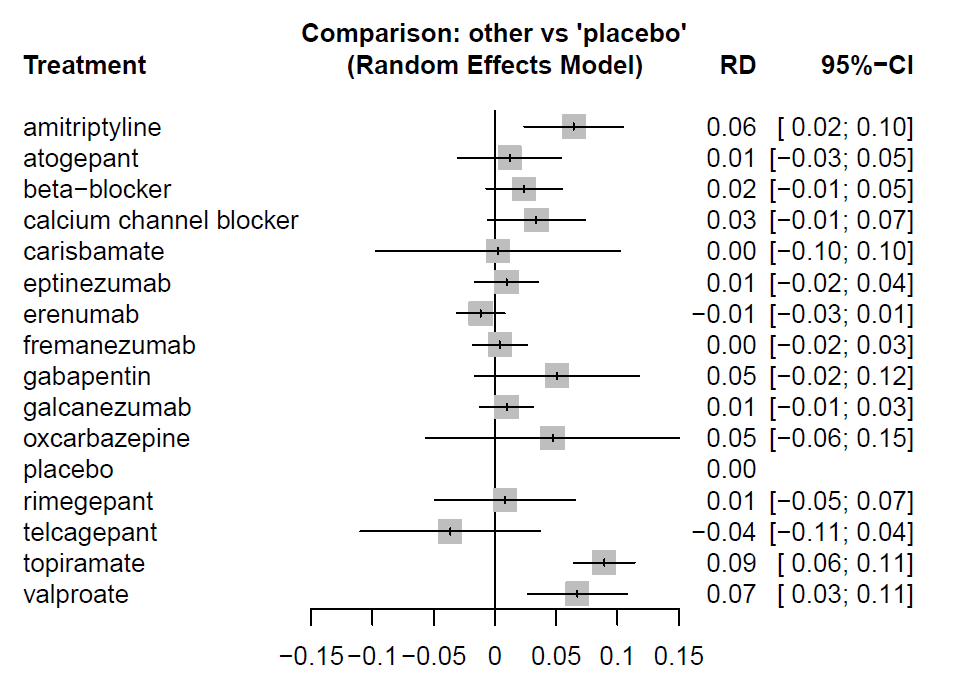


# Supplement 26 – Secondary analysis comparing the effects of telcagepant with other gepnts


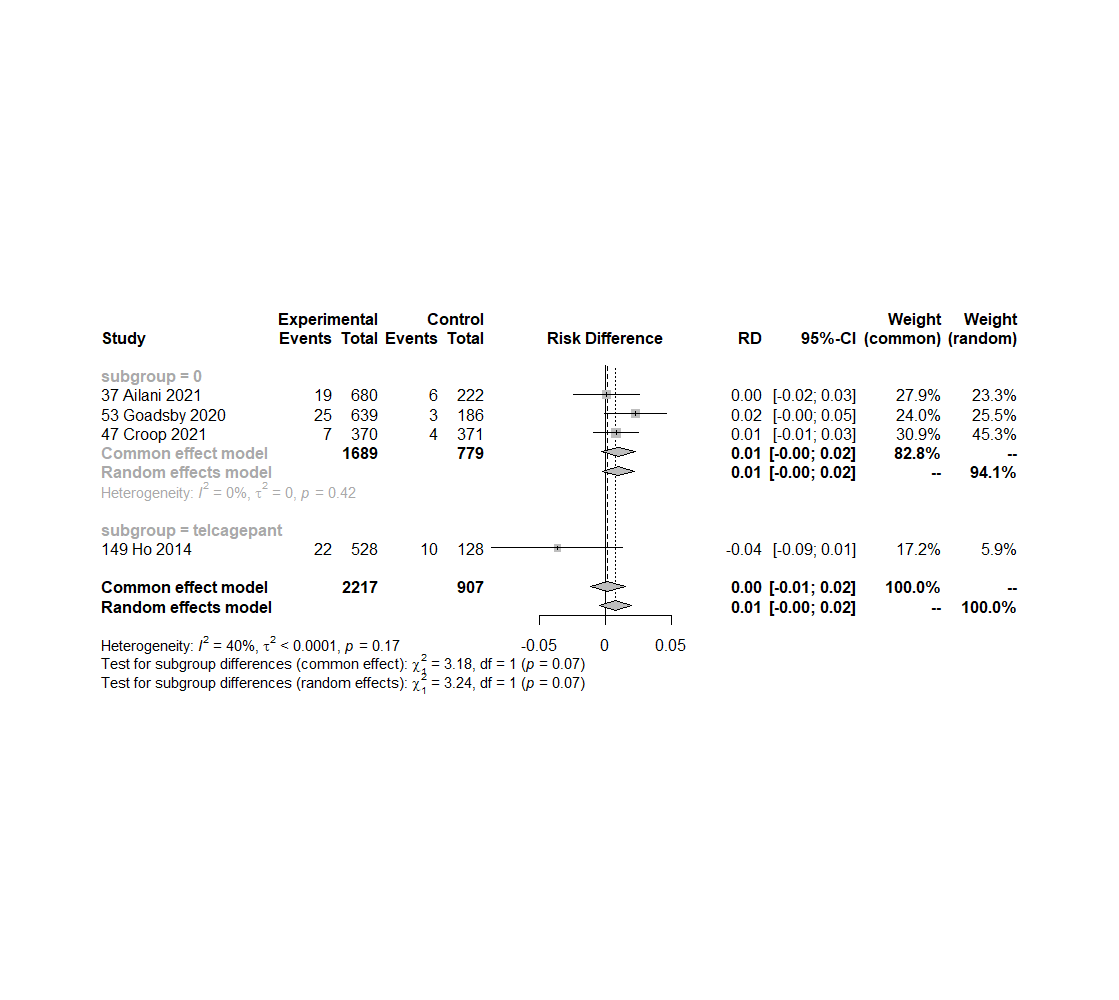


# Supplement 27 – Subgroup analyses for adverse events leading to discontinuation

For subgroup analyses based on baseline monthly migraine days and proportion of patients that had previously used prophylactic drugs, for each comparison against placebo, we calculated the median monthly migraine days and median proportion of patients who had previously used prophylactic drugs across trials, and performed subgroup analyses comparing trials with mean baseline monthly migraine days or proportion of patients who had previously used prophylactic drugs above or equal to/below the median.

| Drug | Risk of bias | | | | | |
| --- | --- | --- | --- | --- | --- | --- |
|  | **Low** | | | **High** | | |
|  | **Number of trials (participants)** | **RD (95% CI)** | **I^2^ (%)** | **Number of trials (participants)** | **RD (95% CI)** | **I^2^ (%)** |
| Amitriptyline | 1 (391) | 0.05 (0.00, 0.11) | NA | 1 (116) | 0.06 (-0.03, 0.15) | NA |
| Beta-blocker | 2 (511) | 0.06 (-0.02, 0.13) | 54 | 2 (220) | 0.02 (-0.03, 0.08) | 50 |
| Calcium channel blockers | NA |  |  | NA |  |  |
| Eptinezumab | 5 (3630) | 0.01 (0.00, 0.02) | 0 | NA |  |  |
| Erenumab | 8 (4528) | 0.00 (-0.01, 0.00) | 0 | NA |  |  |
| Fremanezumab | 7 (4327) | 0.00 (-0.01, 0.01) | 34 | NA |  |  |
| Gabapentin | 2 (669) | 0.05 (0.00, 0.10) | 0 | NA |  |  |
| Galcanezumab | 7 (4097) | 0.01 (0.00, 0.02) | 44 | NA |  |  |
| Gepant | 3 (2468) | 0.01 (0.00, 0.02) | 0 | 1 (656) | -0.04 (-0.09, 0.01) | NA |
| Topiramate | 5 (2092) | 0.12(0.05, 0.19) | 86 | 3 (797) | 0.01 (-0.04, 0.06) | 52 |
| Valproate | 2 (413) | 0.07 (-0.08, 0.21) | 84 | 1 (107) | 0.07 (-0.03, 0.18) | NA |
|  | **Monthly migraine days** | | | | | |
|  | **≤ median** | | | **> median** | | |
|  | **Number of trials (participants)** | **RD (95% CI)** | **I^2^ (%)** | **Number of trials (participants)** | **RD (95% CI)** | **I^2^ (%)** |
| Amitriptyline | NA |  |  | NA |  |  |
| Beta-blocker | 2 (359) | 0.04 (-0.01, 0.09) | 0 | 1 (285) | 0.1 (0.02, 0.18) | NA |
| Calcium channel blockers | NA |  |  | NA |  |  |
| Eptinezumab | 3 (1942) | 0.01 (0.00, 0.02) | 0 | 2 (1688) | 0.01 (0.00, 0.02) | 0 |
| Erenumab | 4 (2470) | 0.00 (-0.00, 0.01) | 0 | 4 (2058) | 0.00 (-0.01, 0.00) | 0 |
| Fremanezumab | 4 (2365) | 0.00 (-0.01, 0.01) | 37 | 3 (1962) | 0.00 (-0.02, 0.01) | 47 |
| Gabapentin | 1 (143) | 0.07 (-0.04, 0.19) | NA | 1 (526) | 0.04 (-0.02, 0.10) | NA |
| Galcanezumab | 4 (1607) | 0.01 (0.00, 0.03) | 56 | 3 (2490) | 0.00 (0.00, 0.01) | 0 |
| Gepant | 2 (1727) | 0.01 (-0.01, 0.03) | 38 | 2 (1397) | -0.01 (-0.05, 0.03) | 64 |
| Topiramate | 4 (1229) | 0.11 (-0.11, 0.22) | 92 | 3 (1339) | 0.04 (0.01, 0.07) | 0 |
| Valproate | 1 (237) | 0.00 (-0.08, 0.07) | NA | 1 (107) | 0.07 (-0.03, 0.18) | NA |
|  | **Previous prophylactic drugs** | | | | | |
|  | **≤ median** | | | **> median** | | |
|  | **Number of trials (participants)** | **RD (95% CI)** | **I^2^ (%)** | **Number of trials (participants)** | **RD (95% CI)** | **I^2^ (%)** |
| Amitriptyline | NA |  |  | NA |  |  |
| Beta-blocker | NA |  |  | NA |  |  |
| Calcium channel blockers | NA |  |  | NA |  |  |
| Eptinezumab | 1 (616) | 0.02 (0.00, 0.04) | NA | 1 (891) | 0.01 (0.00, 0.02) | NA |
| Erenumab | 4 (3078) | 0.00 (-0.01, 0.01) | 29 | 4 (1450) | 0.00 (-0.01, 0.01) | 0 |
| Fremanezumab | 3 (2929) | -0.01 (-0.01, 0.00) | 0 | 3 (1398) | 0.02 (-0.01, 0.04) | 64 |
| Gabapentin | NA |  |  | NA |  |  |
| Galcanezumab | 3 (1894) | 0.02 (0.01, 0.03) | 0 | 2 (1575) | 0.00 (-0.01, 0.01) | 0 |
| Gepant | 2 (1481) | 0.00 (-0.06, 0.06) | 78 | 1 (902) | 0.00 (-0.02, 0.03) | NA |
| Topiramate | NA |  |  | NA |  |  |
| Valproate | 1 (237) | 0.00 (-0.08, 0.07) | NA | 1 (176) | 0.14 (0.05, 0.23) | NA |

**References**

1. Ailani J, Lipton RB, Goadsby PJ, Guo H, Miceli R, Severt L, et al. Atogepant for the Preventive Treatment of Migraine. N Engl J Med. 2021;385(8):695-706.

2. Ashina M, Saper J, Cady R, Schaeffler BA, Biondi DM, Hirman J, et al. Eptinezumab in episodic migraine: A randomized, double-blind, placebo-controlled study (PROMISE-1). Cephalalgia. 2020;40(3):241-54.

3. Ashina M, Lanteri-Minet M, Pozo-Rosich P, Ettrup A, Christoffersen CL, Josiassen MK, et al. Safety and efficacy of eptinezumab for migraine prevention in patients with two-to-four previous preventive treatment failures (DELIVER): a multi-arm, randomised, double-blind, placebo-controlled, phase 3b trial. The Lancet Neurology. 2022;21(7):597-607.

4. Bigal ME, Dodick DW, Rapoport AM, Silberstein SD, Ma Y, Yang R, et al. Safety, tolerability, and efficacy of TEV-48125 for preventive treatment of high-frequency episodic migraine: a multicentre, randomised, double-blind, placebo-controlled, phase 2b study. The Lancet Neurology. 2015;14(11):1081-90.

5. Bigal ME, Dodick DW, Krymchantowski AV, VanderPluym JH, Tepper SJ, Aycardi E, et al. TEV-48125 for the preventive treatment of chronic migraine: Efficacy at early time points. Neurology. 2016;87(1):41-8.

6. Bostani A, Rajabi A, Moradian N, Razazian N, Rezaei M. The effects of cinnarizine versus sodium valproate in migraine prophylaxis. Int J Neurosci. 2013;123(7):487-93.

7. Brandes JL, Saper JR, Diamond M, Couch JR, Lewis DW, Schmitt J, et al. Topiramate for migraine prevention: a randomized controlled trial. JAMA. 2004;291(8):965-73.

8. Cady RK, Mathew N, Diener HC, Hu P, Haas M, Novak GP, et al. Evaluation of carisbamate for the treatment of migraine in a randomized, double-blind trial. Headache. 2009;49(2):216-26.

9. Camporeale A, Kudrow D, Sides R, Wang S, Van Dycke A, Selzler KJ, et al. A phase 3, long-term, open-label safety study of Galcanezumab in patients with migraine. BMC Neurol. 2018;18(1):188.

10. Chowdhury D, Bansal L, Duggal A, Datta D, Koul A, Mundra A, et al. Topiramate versus propranolol for prevention of chronic migraine: A randomized double-blind controlled trial (TOP-PRO study). Journal of the Neurological Sciences. 2021;429(Supplement):117722.

11. Couch JR, Hassanein RS. Amitriptyline in migraine prophylaxis. Archives of neurology. 1979;36(11):695-9.

12. Couch JR. Amitriptyline in the prophylactic treatment of migraine and chronic daily headache. Headache. 2011;51(1):33-51.

13. Croop R, Lipton RB, Kudrow D, Stock DA, Kamen L, Conway CM, et al. Oral rimegepant for preventive treatment of migraine: a phase 2/3, randomised, double-blind, placebo-controlled trial. The Lancet. 2021;397(10268):51-60.

14. Detke HC, Goadsby PJ, Wang S, Friedman DI, Selzler KJ, Aurora SK. Galcanezumab in chronic migraine: The randomized, double-blind, placebo-controlled REGAIN study. Neurology. 2018;91(24):e2211-e21.

15. Diener HC, Foh M, Iaccarino C, Wessely P, Isler H, Strenge H, et al. Cyclandelate in the prophylaxis of migraine: A randomized, parallel, double-blind study in comparison with placebo and propranolol. Cephalalgia. 1996;16(6):441-7.

16. Diener HC, Matias-Guiu J, Hartung E, Pfaffenrath V, Ludin HP, Nappi G, et al. Efficacy and tolerability in migraine prophylaxis of flunarizine in reduced doses: a comparison with propranolol 160 mg daily. Cephalalgia : an international journal of headache. 2002;22(3):209-21.

17. Diener H-C, Tfelt-Hansen P, Dahlof C, Lainez MJA, Sandrini G, Wang S-J, et al. Topiramate in migraine prophylaxis--results from a placebo-controlled trial with propranolol as an active control. Journal of neurology. 2004;251(8):943-50.

18. Diener H-C, Agosti R, Allais G, Bergmans P, Bussone G, Davies B, et al. Cessation versus continuation of 6-month migraine preventive therapy with topiramate (PROMPT): a randomised, double-blind, placebo-controlled trial. The Lancet Neurology. 2007;6(12):1054-62.

19. Dodick DW, Silberstein S, Saper J, Freitag FG, Cady RK, Rapoport AM, et al. The impact of topiramate on health-related quality of life indicators in chronic migraine. Headache. 2007;47(10):1398-408.

20. Dodick DW, Freitag F, Banks J, Saper J, Xiang J, Rupnow M, et al. Topiramate versus amitriptyline in migraine prevention: a 26-week, multicenter, randomized, double-blind, double-dummy, parallel-group noninferiority trial in adult migraineurs. Clin Ther. 2009;31(3):542-59.

21. Dodick DW, Goadsby PJ, Spierings ELH, Scherer JC, Sweeney SP, Grayzel DS. Safety and efficacy of LY2951742, a monoclonal antibody to calcitonin gene-related peptide, for the prevention of migraine: a phase 2, randomised, double-blind, placebo-controlled study. The Lancet Neurology. 2014;13(9):885-92.

22. Dodick DW, Goadsby PJ, Silberstein SD, Lipton RB, Olesen J, Ashina M, et al. Safety and efficacy of ALD403, an antibody to calcitonin gene-related peptide, for the prevention of frequent episodic migraine: a randomised, double-blind, placebo-controlled, exploratory phase 2 trial. The Lancet Neurology. 2014;13(11):1100-7.

23. Dodick DW, Silberstein SD, Bigal ME, Yeung PP, Goadsby PJ, Blankenbiller T, et al. Effect of Fremanezumab Compared With Placebo for Prevention of Episodic Migraine: A Randomized Clinical Trial. JAMA. 2018;319(19):1999-2008.

24. Dodick DW, Ashina M, Brandes JL, Kudrow D, Lanteri-Minet M, Osipova V, et al. ARISE: A Phase 3 randomized trial of erenumab for episodic migraine. Cephalalgia. 2018;38(6):1026-37.

25. Dodick DW, Lipton RB, Silberstein S, Goadsby PJ, Biondi D, Hirman J, et al. Eptinezumab for prevention of chronic migraine: A randomized phase 2b clinical trial. Cephalalgia. 2019;39(9):1075-85.

26. Ferrari MD, Diener HC, Ning X, Galic M, Cohen JM, Yang R, et al. Fremanezumab versus placebo for migraine prevention in patients with documented failure to up to four migraine preventive medication classes (FOCUS): a randomised, double-blind, placebo-controlled, phase 3b trial. Lancet (London, England). 2019;394(10203):1030-40.

27. Freitag FG, Collins SD, Carlson HA, Goldstein J, Saper J, Silberstein S, et al. A randomized trial of divalproex sodium extended-release tablets in migraine prophylaxis. Neurology. 2002;58(11):1652-9.

28. Ghasami K, Mohammad-Beigi A. Comparison of treatment effect of sodium valprovate, propranolol and tricyclic antidepressants in migraine. Pakistan Journal of Biological Sciences. 2009;12(15):1098-101.

29. Ghobadi SH, Jivad N. The prophylactic activity of propranol and nimodipineon migraine headache. World Journal of Medical Sciences. 2013;8(2):144-6.

30. Goadsby PJ, Paemeleire K, Broessner G, Brandes J, Klatt J, Zhang F, et al. Efficacy of erenumab in subjects with episodic migraine with prior preventive treatment failure(s). 2017;37(1):13.

31. Goadsby PJ, Silberstein SD, Yeung PP, Cohen JM, Ning X, Yang R, et al. Long-term safety, tolerability, and efficacy of fremanezumab in migraine: A randomized study. Neurology. 2020;95(18):e2487-e99.

32. Goadsby PJ, Dodick DW, Ailani J, Trugman JM, Finnegan M, Lu K, et al. Safety, tolerability, and efficacy of orally administered atogepant for the prevention of episodic migraine in adults: a double-blind, randomised phase 2b/3 trial. The Lancet Neurology. 2020;19(9):727-37.

33. Goncalves AL, Martini Ferreira A, Ribeiro RT, Zukerman E, Cipolla-Neto J, Peres MFP. Randomised clinical trial comparing melatonin 3 mg, amitriptyline 25 mg and placebo for migraine prevention. Journal of neurology, neurosurgery, and psychiatry. 2016;87(10):1127-32.

34. Hesami O, Shams MR, Ayazkhoo L, Assarzadegan F, Safarpour Lima B, Delavar Kasmaei H, et al. Comparison of Pregabalin and Sodium Valproate in Migraine Prophylaxis: A Randomized Double-Blinded Study. Iranian journal of pharmaceutical research : IJPR. 2018;17(2):783-9.

35. Ho TW, Connor KM, Zhang Y, Pearlman E, Koppenhaver J, Fan X, et al. Randomized controlled trial of the CGRP receptor antagonist telcagepant for migraine prevention. Neurology. 2014;83(11):958-66.

36. Holroyd KA, Cottrell CK, O'Donnell FJ, Cordingley GE, Drew JB, Carlson BW, et al. Effect of preventive (beta blocker) treatment, behavioural migraine management, or their combination on outcomes of optimised acute treatment in frequent migraine: randomised controlled trial. Bmj. 2010;341:c4871.

37. Hu B, Li G, Li X, Wu S, Yu T, Li X, et al. Galcanezumab in episodic migraine: the phase 3, randomized, double-blind, placebo-controlled PERSIST study. The journal of headache and pain. 2022;23(1):90.

38. Kalita J, Bhoi SK, Misra UK. Amitriptyline vs divalproate in migraine prophylaxis: a randomized controlled trial. Acta Neurol Scand. 2013;128(1):65-72.

39. Klapper J. Divalproex sodium in migraine prophylaxis: a dose-controlled study. Cephalalgia : an international journal of headache. 1997;17(2):103-8.

40. Lipton RB, Silberstein S, Dodick D, Cady R, Freitag F, Mathew N, et al. Topiramate intervention to prevent transformation of episodic migraine: the topiramate INTREPID study. Cephalalgia. 2011;31(1):18-30.

41. Lipton RB, Goadsby PJ, Smith J, Schaeffler BA, Biondi DM, Hirman J, et al. Efficacy and safety of eptinezumab in patients with chronic migraine: PROMISE-2. Neurology. 2020;94(13):e1365-e77.

42. Lucking CH, Oestreich W, Schmidt R, Soyka D. Flunarizine vs. propranolol in the prophylaxis of migraine: two double-blind comparative studies in more than 400 patients. Cephalalgia. 1988;8(SUPPL. 8):21-6.

43. Luo N, Di W, Zhang A, Wang Y, Ding M, Qi W, et al. A randomized, one-year clinical trial comparing the efficacy of topiramate, flunarizine, and a combination of flunarizine and topiramate in migraine prophylaxis. Pain medicine (Malden, Mass). 2012;13(1):80-6.

44. Togha M, Rahmat Jirde M, Nilavari K, Ashrafian H, Razeghi S, Kohan L. Cinnarizine in refractory migraine prophylaxis: efficacy and tolerability. A comparison with sodium valproate. The journal of headache and pain. 2008;9(2):77-82.

45. Mathew NT. Prophylaxis of migraine and mixed headache. A randomized controlled study. Headache. 1981;21(3):105-9.

46. Mathew NT, Saper JR, Silberstein SD, Rankin L, Markley HG, Solomon S, et al. Migraine prophylaxis with divalproex. Arch Neurol. 1995;52(3):281-6.

47. Mathew NT, Rapoport A, Saper J, Magnus L, Klapper J, Ramadan N, et al. Efficacy of gabapentin in migraine prophylaxis. Headache. 2001;41(2):119-28.

48. Mei D, Capuano A, Vollono C, Evangelista M, Ferraro D, Tonali P, et al. Topiramate in migraine prophylaxis: a randomised double-blind versus placebo study. Neurological sciences : official journal of the Italian Neurological Society and of the Italian Society of Clinical Neurophysiology. 2004;25(5):245-50.

49. Misra UK, Kalita J, Bhoi SK. Allodynia in migraine: clinical observation and role of prophylactic therapy. The Clinical journal of pain. 2013;29(7):577-82.

50. Mulleners WM, Kim B-K, Láinez MJA, Lanteri-Minet M, Pozo-Rosich P, Wang S, et al. Safety and efficacy of galcanezumab in patients for whom previous migraine preventive medication from two to four categories had failed (CONQUER): a multicentre, randomised, double-blind, placebo-controlled, phase 3b trial. The Lancet Neurology. 2020;19(10):814-25.

51. Reuter U, Goadsby P, Lanteri-Minet M, Ferrari M, Wen S, Klatt J. Efficacy and safety of erenumab in episodic migraine patients with 2-4 prior preventive treatment failures: results from the phase 3b LIBERTY study. 2018;90(24):e2186.

52. Reuter U, Ehrlich M, Gendolla A, Heinze A, Klatt J, Wen S, et al. Erenumab versus topiramate for the prevention of migraine - a randomised, double-blind, active-controlled phase 4 trial. Cephalalgia. 2022;42(2):108-18.

53. Sakai F, Takeshima T, Tatsuoka Y, Hirata K, Lenz R, Wang Y, et al. A Randomized Phase 2 Study of Erenumab for the Prevention of Episodic Migraine in Japanese Adults. Headache. 2019;59(10):1731-42.

54. Sakai F, Ozeki A, Skljarevski V. Efficacy and safety of galcanezumab for prevention of migraine headache in Japanese patients with episodic migraine: A phase 2 randomized controlled clinical trial. Cephalalgia Reports. 2020;3((Sakai) Saitama Neuropsychiatric Institute, Saitama, Japan(Ozeki) Eli Lilly Japan K.K, Kobe, Japan(Skljarevski) Eli Lilly and Company, Indianapolis, IN, United States).

55. Sakai F, Suzuki N, Kim BK, Igarashi H, Hirata K, Takeshima T, et al. Efficacy and safety of fremanezumab for chronic migraine prevention: Multicenter, randomized, double-blind, placebo-controlled, parallel-group trial in Japanese and Korean patients. Headache. 2021;61(7):1092-101.

56. Sakai F, Suzuki N, Kim BK, Tatsuoka Y, Imai N, Ning X, et al. Efficacy and safety of fremanezumab for episodic migraine prevention: Multicenter, randomized, double-blind, placebo-controlled, parallel-group trial in Japanese and Korean patients. Headache. 2021;61(7):1102-11.

57. Sargent J, Solbach P, Damasio H, Baumel B, Corbett J, Eisner L, et al. A comparison of naproxen sodium to propranolol hydrochloride and a placebo control for the prophylaxis of migraine headache. Headache. 1985;25(6):320-4.

58. Silberstein SD, Neto W, Schmitt J, Jacobs D, Group M-S. Topiramate in migraine prevention: results of a large controlled trial. Archives of neurology. 2004;61(4):490-5.

59. Silberstein SD, Hulihan J, Rezaul Karim M, Wu S-C, Jordan D, Karvois D, et al. Efficacy and tolerability of topiramate 200 mg/d in the prevention of migraine with/without aura in adults: A randomized, placebo-controlled, double-blind, 12-week pilot study. Clinical Therapeutics. 2006;28(7):1002-11.

60. Silberstein S, Saper J, Berenson F, Somogyi M, McCague K, D'Souza J. Oxcarbazepine in migraine headache: a double-blind, randomized, placebo-controlled study. Neurology. 2008;70(7):548-55.

61. Silberstein S, Lipton R, Dodick D, Freitag F, Mathew N, Brandes J, et al. Topiramate treatment of chronic migraine: a randomized, placebo-controlled trial of quality of life and other efficacy measures. Headache. 2009;49(8):1153-62.

62. Silberstein SD, Dodick DW, Lindblad AS, Holroyd K, Harrington M, Mathew NT, et al. Randomized, placebo-controlled trial of propranolol added to topiramate in chronic migraine. Neurology. 2012;78(13):976-84.

63. Silberstein S, Goode-Sellers S, Twomey C, Saiers J, Ascher J. Randomized, double-blind, placebo-controlled, phase II trial of gabapentin enacarbil for migraine prophylaxis. Cephalalgia. 2013;33(2):101-11.

64. Silberstein SD, Dodick DW, Bigal ME, Yeung PP, Goadsby PJ, Blankenbiller T, et al. Fremanezumab for the Preventive Treatment of Chronic Migraine. N Engl J Med. 2017;377(22):2113-22.

65. Skljarevski V, Matharu M, Millen BA, Ossipov MH, Kim BK, Yang JY. Efficacy and safety of galcanezumab for the prevention of episodic migraine: Results of the EVOLVE-2 Phase 3 randomized controlled clinical trial. Cephalalgia. 2018;38(8):1442-54.

66. Skljarevski V, Oakes TM, Zhang Q, Ferguson MB, Martinez J, Camporeale A, et al. Effect of Different Doses of Galcanezumab vs Placebo for Episodic Migraine Prevention: A Randomized Clinical Trial. JAMA Neurol. 2018;75(2):187-93.

67. Sorensen PS, Larsen BH, Rasmussen MJ, Kinge E, Iversen H, Alslev T, et al. Flunarizine versus metoprolol in migraine prophylaxis: a double-blind, randomized parallel group study of efficacy and tolerability. Headache. 1991;31(10):650-7.

68. Sudilovsky A, Elkind AH, Ryan RE, Sr., Saper JR, Stern MA, Meyer JH. Comparative efficacy of nadolol and propranolol in the management of migraine. Headache. 1987;27(8):421-6.

69. Sun H, Dodick DW, Silberstein S, Goadsby PJ, Reuter U, Ashina M, et al. Safety and efficacy of AMG 334 for prevention of episodic migraine: a randomised, double-blind, placebo-controlled, phase 2 trial. The Lancet Neurology. 2016;15(4):382-90.

70. Takeshima T, Sakai F, Hirata K, Imai N, Matsumori Y, Yoshida R, et al. Erenumab treatment for migraine prevention in Japanese patients: Efficacy and safety results from a Phase 3, randomized, double-blind, placebo-controlled study. Headache. 2021;61(6):927-35.

71. Tepper S, Ashina M, Reuter U, Brandes JL, Doležil D, Silberstein S, et al. Safety and efficacy of erenumab for preventive treatment of chronic migraine: a randomised, double-blind, placebo-controlled phase 2 trial. The Lancet Neurology. 2017;16(6):425-34.

72. van de Ven LL, Franke CL, Koehler PJ. Prophylactic treatment of migraine with bisoprolol: a placebo-controlled study. Cephalalgia : an international journal of headache. 1997;17(5):596-9.

73. Wang SJ, Roxas AA, Jr., Saravia B, Kim BK, Chowdhury D, Riachi N, et al. Randomised, controlled trial of erenumab for the prevention of episodic migraine in patients from Asia, the Middle East, and Latin America: The EMPOwER study. Cephalalgia. 2021;41(13):1285-97.
